# Supplementary material for: Identifying research priorities for road safety in Nepal: a Delphi study
Source: BMJ Open. 2022 Apr 13;12(4):e059312. doi: 10.1136/bmjopen-2021-059312 (PMC9014064; doi:10.1136/bmjopen-2021-059312)
Supplement: Supplementary data [file bmjopen-2021-059312supp001.pdf]

Interview guide

Road Safety Research Prioritisation study

**Pillar 1: Road safety management**

(To be read to each participant for this pillar) *This pillar focuses on strengthening multi-agency capacity for road safety. It includes activities such as putting into practice major UN road safety conventions, establishing a multi-sectoral national agency to lead road safety activities, developing a national road safety strategy and setting realistic and long-term targets for related activities with sufficient funding for implementation. It also calls for the development of data systems to effectively monitor and evaluate activities.*

Questions and prompts

- What is your job title and what is the focus of your responsibilities for this position?
- How long have you been in this role?
  - Prompt: How these responsibilities are determined?
- From the description of Pillar 1, what is the status of activities for this pillar in Nepal?
- From your experience, what is going well?
  - *Prompt: Why do you think it is going well?*
- From you experience what has not yet happened or is not working well?
  - *Prompt: Why do you think it is not working well?*
- What challenges are you facing to achieve your desired objectives for road safety?
  - *Prompts:*
    - *challenges regarding to have a lead agency?*
    - *challenges regarding national strategy?*
    - *challenges regarding data generation?*
    - *challenges regarding monitoring and evaluation?*
- What information or evidence do you think would help you achieve these objectives?
- Can you think of any gaps in the research available to you, related to these activities in pillar 1?
- Would you like to add anything which we have not covered during this conversation?

At the end of the interview

- Explain that this is the end of the interview.
- Thank the participant for their time.
- Explain that the information they have given will be used to create a list of possible research ideas to improve road safety in Nepal.
- Explain that they will be invited to the next stage of the study where they will hear all of the research questions we have identified, and they will be invited to tell us which ones they think are the most important.
- Ask if they have any questions before you go.

Pant PR, *et al. BMJ Open* 2022; 12:e059312. doi: 10.1136/bmjopen-2021-059312

Pillar 2. Safer roads and mobility

(To be read to each participant for this pillar) *This pillar highlights the need to improve the safety of road networks and infrastructure for the benefit of all road users, including the pedestrians, bicyclists and motorcyclists. Activities include considering safety during the planning, design, construction and operation of roads; making sure that roads are regularly assessed for safety; and encouraging the relevant authorities to consider all forms of transport and types of safe infrastructure when they respond to the mobility needs of road users.*

Suggested questions and prompts

- What is your job title and what is the focus of your responsibilities for this position?
- How long have you been in this role?
    - Prompt: How these responsibilities are determined?
- From the description of Pillar 2, what is the status of activities for this pillar in Nepal?
- From your experience, what is going well?
    - *Prompt: Why do you think it is going well?*
- From you experience what has not yet happened or is not working well?
    - *Prompt: Why do you think it is not working well?*
- What challenges you are facing to achieve your desired objectives for road safety?
    - *Prompts*
        - *challenges to promote road safety ownership and accountability?*
        - *challenges promoting (addressing) the needs of all road users?*
        - *challenges relating to designing, building or maintaining roads?*
- What information or evidence do you think would help you achieve these objectives?
- Can you think of any gaps in the research available to you, related to these activities?
- Would you like to add anything which we have not covered during this conversation?

At the end of the interview

- Explain that this is the end of the interview.
- Thank the participant for their time.
- Explain that the information they have given will be used to create a list of possible research ideas to improve road safety in Nepal.
- Explain that they will be invited to the next stage of the study where they will hear all of the research questions we have identified and they will be invited to tell us which ones they think are the most important.
- Ask if they have any questions before you go.

Pillar 3. Safer vehicles

(To be read to each participant for this pillar) *Poor vehicle standards contribute to a significant number of crashes and casualties. This pillar encourages use of best practice vehicle safety standards and technology to promote safety. Activities may include implementing new car assessment programmes (such as NCAP safety ratings) and vehicle safety checks on existing vehicles to ensure they are equipped with minimum safety features, such as seat-belts to minimise the impact of crashes to occupants, and working lights and brakes.*

Suggested questions and prompts

- What is your job title and what is the focus of your responsibilities for this position?

Pant PR, *et al. BMJ Open* 2022; 12:e059312. doi: 10.1136/bmjopen-2021-059312

- How long have you been in this role?
    - Prompt: How these responsibilities are determined?
- From the description of Pillar 3, what is the status of activities for this pillar in Nepal?
- From your experience, what is going well?
    - *Prompt: Why do you think it is going well?*
- From you experience what has not happened or is not working well?
    - *Prompts*
        - *Why do you think it is not working well?*
        - *What are your views on the New Car Assessment Programme (NCAP)?*
        - *do we have good vehicle-related laws that could promote the import of safer vehicles or the maintenance of existing vehicles?*
- What challenges you are facing to achieve desired objectives for road safety?
        - *challenges to harmonise international motor vehicle safety regulations with national laws?*
        - *research about safety technologies designed to reduce risk to vulnerable road users.*
- What information or evidence do you think would help you achieve these objectives?
- Can you think of any gaps in the research available to you, related to these activities?
- Would you like to add something else which we have not covered during this conversation?

At the end of the interview

- Explain that this is the end of the interview.
- Thank the participant for their time.
- Explain that the information they have given will be used to create a list of possible research ideas to improve road safety in Nepal.
- Explain that they will be invited to the next stage of the study where they will hear all of the research questions we have identified and they will be invited to tell us which ones they think are the most important.
- Ask if they have any questions before you go.

Pillar 4. Safer road users

(To be read to each participant for this pillar) *Pillar 4 focuses on developing comprehensive programmes to improve the behaviour of all road users. Activities include the adoption of model road safety legislation and sustained or increased enforcement or road safety laws and standards. These efforts are combined with public awareness and education to increase uptake of behaviours that keep people safe (e.g. seat-belt and helmet wearing) and to reduce behaviours that cause harm (e.g. speeding, taking alcohol or drugs when driving) and other risks. It also calls for activities to reduce work-related road traffic injuries and promoted the establishment of graduated driver licensing programmes for novice drivers.*

<u>Suggested questions and prompts</u>

- What is your job title and what is the focus of your responsibilities for this position?
- How long have you been in this role?
    - Prompt: How these responsibilities are determined?
- From the description of Pillar 4, what is the status of activities for this pillar in Nepal?
- From your experience, what is going well?

  - *Prompt: Why do you think it is going well?*
- From you experience what has not happened or is not working well?
  - *Prompt: Why do you think it is not working well?*
- What challenges you are facing to achieve desired objectives for road safety?
  - *Prompts*
    - *what is the status of law enforcement?*
    - *what could be done to strengthen road safety law enforcement?*
    - *How good is the uptake of safe driver / passenger behaviours (e.g. seatbelt / helmet use)?*
    - *What role do driving licences play in road safety?*
    - *Are there any gaps in what we know about road user behaviours and how to change them?*
    - *what about gaps in legislation or how it is enforced relating to road users behaviour?*
- What new information or evidence do you think would help you to improve the uptake of safe road user behaviours?
- Can you think of any gaps in the research available to you, related to these activities in Pillar 4?
- Would you like to add something else which we have not covered during this conversation?

At the end of the interview
- Explain that this is the end of the interview.
- Thank the participant for their time.
- Explain that the information they have given will be used to create a list of possible research ideas to improve road safety in Nepal.
- Explain that they will be invited to the next stage of the study where they will hear all of the research questions we have identified and they will be invited to tell us which ones they think are the most important.
- Ask if they have any questions before you go.

Pillar 5. Post-crash response

(To be read to each participant for this pillar) *Pillar 5 addresses the need to improve the response to post-crash emergencies and the ability of health and other systems to provide appropriate emergency treatment and long-term rehabilitation for crash victims. The development and improvement of pre-hospital care systems, hospital trauma care systems, and rehabilitation along with long-term medical support to victims and a single emergency response number, are the main elements of post-impact care.*

Suggested questions and prompts
- What is your job title and what is the focus of your responsibilities for this position?
- How long have you been in this role?
  - Prompt: How these responsibilities are determined?
- From the description of Pillar 5, what is the status of activities for this pillar in Nepal?
- From your experience, what is going well?
  - *Prompt: Why do you think it is going well?*
- From you experience what is left behind or not working well?
  - *Prompt: Why do you think it is not working well?*

Pant PR, *et al. BMJ Open* 2022; 12:e059312. doi: 10.1136/bmjopen-2021-059312

- What challenges you are facing to achieve desired objectives for road safety?
    - *Prompts*
        - *why there is no single nationwide telephone number for emergency services i.e. ambulances?*
        - *How do you see the performance of hospital trauma services in Nepal?*
- What new information or evidence do you think would help improve the provision of good post-crash response and care in Nepal?
- Can you think of any gaps in the research or information available to you in regard to the recommendations in Pillar 5?
- Would you like to add something else which we have not covered during this conversation?

At the end of the interview

- Explain that this is the end of the interview.
- Thank the participant for their time.
- Explain that the information they have given will be used to create a list of possible research ideas to improve road safety in Nepal.
- Explain that they will be invited to the next stage of the study where they will hear all of the research questions we have identified and they will be invited to tell us which ones they think are the most important.
- Ask if they have any questions before you go.

Below is the Nepali translation of the Guide.

UWE Bristol University of the West of England

NIRC नेपाल इन्जरी अनुसन्धान केन्द्र Nepal Injury Research Centre

अन्तरवार्ता निर्देशिका

नेपालमा सडक सुरक्षाका लागि अनुसन्धान प्राथमिकताहरू पहिचान गर्ने अध्ययन

स्तम्भ १. सडक सुरक्षा व्यवस्थापन

(To be read to each participant for this pillar)

सडक सुरक्षाको यो स्तम्भ देशमा सडक सुरक्षाको लागि बहु-संस्थागत क्षमता सुदृढीकरणमा केन्द्रित छ। यस स्तम्भमा देशमा सडक सुरक्षाका लागि संयुक्त राष्ट्रसंघीय महासन्धीहरूलाई अभ्यास गर्ने, सडक सुरक्षा गतिविधिहरूको नेतृत्व गर्ने बहु-पक्षीय राष्ट्रिय निकायको स्थापना गर्ने, राष्ट्रिय सडक सुरक्षा रणनीति विकास गर्ने र सम्बन्धित गतिविधिहरूको कार्यान्वयनको लागि यथार्थवादी र दीर्घकालीन लक्ष्यहरूको निर्धारण गर्नाका साथै तिनको कार्यान्वयनका लागि पर्याप्त रकमको सुनिश्चतता गर्ने कृयाकलापहरू पर्दछन्। यस स्तम्भले उपर्युक्त कृयाकलापहरूको प्रभावकारी रूपमा अनुगमन र मूल्याङ्कन गर्नको लागि तथ्यांक प्रणालीको विकासको लागि पनि आह्वान गर्दछ।

<u>Questions and prompts</u>

- तपाई कुन पदमा कार्यरत हुनुहुन्छ र तपाईंको मुख्य जिम्मेवारीहरु के के हुन्?
- तपाई यस पदमा कति समयदेखि हुनुहुन्छ ?
  - प्रम्ट: यस पदका लागि जिम्मेवारी कसरी निर्धारण भएका हुन्छन् ?
- स्तम्भ १ को वर्णनबाट तपाईं को विचारमा नेपालमा यस स्तम्भ अन्तर्गत गरिएका कृयाकलापहरूको अवस्था कस्तो छ ?
- तपाईको अनुभवबाट यस स्तम्भ अन्तरगतका कुन कुन कृयाकलापहरू राम्रोसँग अघि बढिरहेका छन् ?
  - <u>प्रम्ट</u>: तपाईंको विचारमा किन यो राम्रो हुँदैछ **?**
- तपाईको अनुभवमा यस स्तम्भ अन्तर्गतका कुन कामहरू राम्ररी भइरहेका छैन वा राम्रोसँग गर्न सकिएको छैन ?
  - <u>प्रम्ट</u>: तपाईंको विचारमा किन ती कृयाकलापहरू राम्रोसँग गर्न सकिएको छैन ?
- सडक सुरक्षाको लागि अपेक्षित उद्देश्यहरू प्राप्त गर्न तपाईँले के कस्ता चुनौतीहरू सामना गर्नु परेको छ ?
    - सडक सुरक्षाको नेतृत्व गर्ने संस्था वा निकायको स्थापनाबारे चुनौतिहरू?
    - राष्ट्रिय रणनीति बनाउने सम्बन्धी चुनौतिहरू?
    - तथ्यांक उत्पादन (Data generation) सम्बन्धी चुनौतिहरू?
    - अनुगमन र मूल्यांकन सम्बन्धी चुनौतिहरू?
- कस्ता जानकारी वा तथ्यहरू (Evidence) उपलब्ध भइदिएको भए तपाईंलाई यी उद्देश्यहरू प्राप्त गर्न मद्दत पुग्थ्यो जस्तो लाग्छ ?
- के तपाईँ स्तम्भ १ का यी गतिविधिहरूसँग सम्बन्धित अनुसन्धानमा कुनै कमी वा अन्तरहरू (Research Gaps) बारे सोचेर केही बताउन सक्नुहुन्छ ?
- हाम्रो यस कुराकानीको क्रममा केहि कुरा छुटेको पाउनु भए कृपया थप्नुहोस?

Pant PR, *et al. BMJ Open* 2022; 12:e059312. doi: 10.1136/bmjopen-2021-059312

At the end of the interview

- हामी अन्तर्वार्ताको अन्त्यमा पुग्यौं
- तपाईंको सहभागिताको लागि धन्यवाद
- तपाईंले दिनुभएको जानकारीले नेपालमा सडक सुरक्षाको स्थितिमा सुधार ल्याउन सम्भावित अनुसन्धानको लागि सुची बनाउन मद्दत गर्नेछ।
- हामी तपाईलाई यस अध्ययनको दोस्रो चरणको लागि पनि सम्पर्क गर्नेछौं जसमा हामी अन्तरवार्ताहरूबाट पहिचान गरेका सबै अनुसन्धान प्रश्नहरु प्रस्तुत गर्नेछौं र त्यसमध्ये कुन सबैभन्दा महत्वपुर्ण छ भनेर तपाईंको प्रतिक्रिया लिनेछौं ।
- अन्तिममा तपाईंको केहि प्रश्न छ कि?

स्तम्भ २. सुरक्षित सडक र गतिशिलता

(To be read to each participant for this pillar)
यो स्तम्भले सबै पैदलयात्री, साइकल चालक, मोटरसाइकल चालक लगायत सबै सडक प्रयोगकर्ताको फाइदाको लागि आवश्यक सुरक्षित सडक संजाल एवं पूर्वाधार सुधारलाई जोड दिन्छ । यस अन्तरगतका कृयाकलापहरूमा सडकको योजना, निर्माण तथा संचालनका चरणहरूमा नैं सडक सुरक्षालाई ध्यानमा राख्नु पर्ने; सडकको सुरक्षाको नियमित मुल्यांकन गरिनेछ भनेर सुनिस्चित गर्ने; र सम्बद्ध अधिकारीलाई सडक प्रयोगकर्ताको गतिशिलता (mobility) आवश्यकतालाई सम्बोधन गर्दा सबै किसिम र प्रकारका यातायात तथा पूर्वाधारलाई सुरक्षित पार्न प्रोत्साहन गर्ने जस्ता क्रियाकलापहरु पर्छन् ।

Suggested questions and prompts

- तपाई कुन पदमा कार्यरत हुनुहुन्छ र तपाईंको मुख्य जिम्मेवारीहरु के के हुन्?
- तपाई यस पदमा कति समयदेखि हुनुहुन्छ ?
    - प्रम्प्टः यस पदका लागि जिम्मेवारी कसरी निर्धारण भएका हुन्छन् ?
- स्तम्भ २ को वर्णनबाट तपाईं को विचारमा नेपालमा यस स्तम्भ अन्तरगत गरिएका कृयाकलापहरूको अवस्था कस्तो छ ?
- तपाईंको अनुभवबाट यस स्तम्भ अन्तरगतका कुन कुन कृयाकलापहरू राम्रोसँग अघि बढिरहेका छन् ?
    - प्रम्प्ट: तपाईंको विचारमा किन यो राम्रो हुँदैछ ?
- तपाईंको अनुभवमा यस स्तम्भ अन्तरगतका कुन कामहरू राम्ररी भइरहेका छैन वा राम्रोसँग गर्न सकिएको छैन ?
    - प्रम्प्ट: तपाईंको विचारमा किन ती कृयाकलापहरू राम्रोसँग गर्न सकिएको छैन ?
- सडक सुरक्षाको लागि अपेक्षित उद्देश्यहरू प्राप्त गर्न तपाइँले के कस्ता चुनौतीहरू सामना गर्दै हुनुहुन्छ ?
        - सडक सुरक्षाको स्वामित्व र उत्तरदायित्व प्रवर्धनसम्बन्धी चुनौती?
        - सबै सडक प्रयोगकर्ताहरूको आवश्यकतालाई सम्बोधन गर्नेबारे चुनौतीहरू?
        - सडक डिजाइन, निर्माण वा मर्मतसँग सम्बन्धी चुनौतिहरू?
- कस्ता जानकारी वा तथ्यहरू (Evidence) उपलव्ध भइदिएको भए तपाईंलाई यी उद्देश्यहरू प्राप्त गर्न मद्दत पुग्थ्यो जस्तो लाग्छ ?
- के तपाइँ स्तम्भ २ का यी गतिविधिहरुसंग सम्बन्धित अनुसन्धानमा कुनै कमी वा अन्तरहरू (Gaps) बारे सोचेर केही बताउन सक्नुहुन्छ ?

Pant PR, *et al. BMJ Open* 2022; 12:e059312. doi: 10.1136/bmjopen-2021-059312

- हाम्रो यस कुराकानीको क्रममा केहि कुरा छुटेको पाउनु भए कृपया थप्नुहोस?

At the end of the interview

- हामी अन्तर्वार्ताको अन्त्यमा पुग्यौं
- तपाईंको सहभागिताको लागि धन्यवाद
- तपाईंले दिनुभएको जानकारीले नेपालमा सडक सुरक्षाको स्थितिमा सुधार ल्याउन सम्भावित अनुसन्धानको लागि सुची बनाउन मद्दत गर्नेछ।
- हामी तपाईलाई यस अध्ययनको दोस्रो चरणको लागि पनि सम्पर्क गर्नेछौं जसमा हामी अन्तरवार्ताहरूबाट पहिचान गरेका सबै अनुसन्धान प्रश्नहरु प्रस्तुत गर्नेछौं र त्यसमध्ये कुन सबैभन्दा महत्वपुर्ण छ भनेर तपाईंको प्रतिक्रिया लिनेछौं ।
- अन्तिममा तपाईंको केहि प्रश्न छ कि?

स्तम्भ ३ सुरक्षित वाहनहरू

(To be read to each participant for this pillar)
कमसल मापदण्ड भएका वाहनहरूले उल्लेखनीय संख्यामा सडक दुर्घटना र हताहती गराइरहेका हुन्छन्। यस स्तम्भले सुरक्षा प्रबर्धन गर्न उत्तम अभ्यास, वाहन सुरक्षाका मानकहरू र प्रविधिको प्रयोग गर्न प्रोत्साहन गर्दछ। यस अन्तरगतका गतिविधिहरूमा नयाँ कारको सुरक्षा मुल्यांकन कार्यक्रम (NCAP सुरक्षा रेटिंगहरू) को कार्यान्वयन गर्ने, विद्यमान सवारी साधनहरूमा न्यूनतम सुरक्षा सुविधाहरू भएको सुनिश्चत गर्न वाहन सुरक्षा जाँचहरू समावेश गर्ने, जस्तै दुर्घटनामा परेका व्यक्तिमा दुर्घटनाको प्रभाव कम गर्न सीट बेल्ट जोडिएको र बत्ती एवम् ब्रेक ठीक अवस्थामा छ भन्ने सुनिश्चत गर्दछ ।

<u>Suggested questions and prompts</u>

- तपाई कुन पदमा कार्यरत हुनुहुन्छ र तपाईंको मुख्य जिम्मेवारीहरु के के हुन्?
- तपाई यस पदमा कति समयदेखि हुनुहुन्छ ?
    - प्रम्प्टः यस पदका लागि जिम्मेवारी कसरी निर्धारण भएका हुन्छन् ?
- स्तम्भ ३ को वर्णनबाट तपाईं को विचारमा नेपालमा यस स्तम्भ अन्तरगत गरिएका कृयाकलापहरूको अवस्था कस्तो छ ?
- तपाईको अनुभवबाट यस स्तम्भ अन्तरगतका कुन कुन कृयाकलापहरू राम्रोसँग अघि बढिरहेका छन् ?
    - <u>प्रम्प्ट</u>: तपाईंको विचारमा किन यो राम्रो हुँदैछ **?**
- तपाईको अनुभवमा यस स्तम्भ अन्तरगतका कुन कामहरू राम्ररी भइरहेका छैन वा राम्रोसँग गर्न सकिएको छैन ?
    - तपाईंको विचारमा किन ती कृयाकलापहरू राम्रोसँग गर्न सकिएको छैन ?
    - NCAP सुरक्षा रेटिंगहरू (अथवा नयाँ कारको सुरक्षा मुल्यांकन कार्यक्रम) सम्बन्धमा तपाइँको के विचार छ?
    - के हामीसँग सवारी साधन सम्बन्धी राम्रा कानूनहरू छन् जसले बढी सुरक्षित गाडीहरूको आयात बढाउने वा विद्यमान सवारी साधनहरूमा सुधार वा मर्मत गरी तिनलाइ सुरक्षित पार्न मद्दत गर्दछन्?
- सडक सुरक्षाको लागि अपेक्षित उद्देश्यहरू प्राप्त गर्न तपाइँले के कस्ता चुनौतीहरू सामना गर्दै हुनुहुन्छ ?
    - राष्ट्रिय कानूनहरूलाई अन्तर्राष्ट्रिय मोटर वाहन सुरक्षा नियमहरुसँग सामंजस्यता गर्दाका चुनौतिहरु?

    - जोखिममा रहेका सडक प्रयोगकर्ताहरूका जोखिम कम गर्न डिजाइन गरिएको सुरक्षा प्रविधिहरूका बारे अनुसन्धानसम्बन्धी चुनौतिहरुरू
- कस्ता जानकारी वा तथ्यहरू (Evidence) उपलब्ध भइदिएको भए तपाईंलाई यी उद्देश्यहरू प्राप्त गर्न मद्दत पुग्थ्यो जस्तो लाग्छ ?
- के तपाइँ स्तम्भ ३ का यी गतिविधिहरुसंग सम्बन्धित अनुसन्धानमा कुनै कमी वा अन्तरहरू (Gaps) बारे सोचेर केही बताउन सक्नुहुन्छ ?
- हाम्रो यस कुराकानीको क्रममा केहि कुरा छुटेको पाउनु भए कृपया थप्नुहोस?

At the end of the interview

- हामी अन्तर्वार्ताको अन्त्यमा पुग्यौं
- तपाईंको सहभागिताको लागि धन्यवाद
- तपाईंले दिनुभएको जानकारीले नेपालमा सडक सुरक्षाको स्थितिमा सुधार ल्याउन सम्भावित अनुसन्धानको लागि सुची बनाउन मद्दत गर्नेछ।
- हामी तपाईलाई यस अध्ययनको दोस्रो चरणको लागि पनि सम्पर्क गर्नेछौं जसमा हामी अन्तरवार्ताहरूबाट पहिचान गरेका सबै अनुसन्धान प्रश्नहरु प्रस्तुत गर्नेछौं र त्यसमध्ये कुन सबैभन्दा महत्वपुर्ण छ भनेर तपाईंको प्रतिक्रिया लिनेछौं ।
- अन्तिममा तपाईंको केहि प्रश्न छ कि?

स्तम्भ ४ सुरक्षित सडक प्रयोगकर्ताहरू

(To be read to each participant for this pillar)
स्तम्भ ४ सबै सडक प्रयोगकर्ताहरूको व्यवहार सुधार गर्न व्यापक कार्यक्रमहरू विकासमा केन्द्रित छ। यस अन्तरगत उदाहरणीय सडक सुरक्षा कानून र मापदण्ड अपनाउने र तिनको निरन्तर वा बढ्दो क्रममा पालनामा गराउने गतिविधिहरू समावेश छन। यस्ता प्रयासहरूमा जनचेतना र मानिसहरुलाई सुरक्षित राख्ने उपायहरू जस्तैः सीट बेल्ट र हेलमेट लगाउने बानी प्रवर्द्धन गर्न र तीव्र गतिमा वाहन चलाउने, रक्सी वा मादकपदार्थ सेवन गरी गाडी चलाउने र यस्तै अन्य जोखिमपूर्ण व्यवहारमा कमी ल्याउन व्यवहारिक शिक्षा दिने कृयाकलापहरू पनि समावेश छन्। यस स्तम्भले कामसँग सम्बन्धित (पेशागत) सडक दुर्घटनाहरू कम गर्ने गतिविधिहरूको लागि आह्वान गर्दछ र भरखरै चालक अनुमतिपत्र (लाइसेन्स) लिएका नयाँ चालकहरूको लागि उनीहरूको सवारी चालक अनुमतिपत्र (लाइसेन्स) लाई क्रमैसँग स्तरोन्नति गर्ने (graduated driver licensing) कार्यक्रमहरूलाई बढावा दिन अह्वान गरेको छ।

<u>Suggested questions and prompts</u>

- तपाई कुन पदमा कार्यरत हुनुहुन्छ र तपाईंको मुख्य जिम्मेवारीहरु के के हुन्?
- तपाई यस पदमा कति समयदेखि हुनुहुन्छ ?
    - प्रम्प्टः यस पदका लागि जिम्मेवारी कसरी निर्धारण भएका हुन्छन् ?
- स्तम्भ ४ को वर्णनबाट तपाईं को विचारमा नेपालमा यस स्तम्भ अन्तरगत गरिएका कृयाकलापहरूको अवस्था कस्तो छ ?
- तपाईंको अनुभवबाट यस स्तम्भ अन्तरगतका कुन कुन कृयाकलापहरू राम्रोसँग अघि बढिरहेका छन् ?
    - <u>प्रम्प्ट</u>: तपाईंको विचारमा किन यो राम्रो हुँदैछ **?**

Pant PR, *et al. BMJ Open* 2022; 12:e059312. doi: 10.1136/bmjopen-2021-059312

- तपाईको अनुभवमा यस स्तम्भ अन्तरगतका कुन कामहरू राम्ररी भइरहेका छैन वा राम्रोसँग गर्न सकिएको छैन ?
    - तपाईंको विचारमा किन ती कृयाकलापहरू राम्रोसँग गर्न सकिएको छैन ?
- सडक सुरक्षाको लागि अपेक्षित उद्देश्यहरू प्राप्त गर्न तपाइँले के कस्ता चुनौतीहरू सामना गर्दै हुनुहुन्छ ?
        - कानूनको पालना र कार्यान्वयनको स्थिति कस्तो छ?
        - सडक सुरक्षा कानूनको पालनालाई सुदृढ पार्न के गर्न सकिन्छ?
        - सुरक्षित चालक / यात्री व्यवहारको अवलम्बन गर्ने चलन कस्तो छ (उदाहरणको लागि सीटबेल्ट / हेलमेट प्रयोग, मा.प.से.)
        - सडक सुरक्षाको लागि चालक अनुमतिपत्र (लाइसेन्स) के भूमिका खेल्छ?
        - सडक प्रयोगकर्ताका व्यवहारसँग सम्बन्धित कुनैं कमीकमजोरीहरू हामीलाई थाहा छन् ? तिनलाई कसरी परिवर्तन गर्न सकिन्छ ?
        - सडक सुरक्षा सम्बन्धी कानूनी प्रावधानमा के कस्ता कमी कमजोरी छन् ? तिनको पालनालाई कसरी सडक प्रयोगकर्ताहरूको व्यवहारसँग जोडिएकोछ ?
- कुन नयाँ जानकारी वा तथ्य उपलब्ध भइदिए सडक प्रयोगकर्ताहरूको सुरक्षित व्यवहार अवलम्बनलाई सुधार गर्न मद्दत पुग्नेछ?
- के तपाइँ स्तम्भ ४ का यी गतिविधिहरुसंग सम्बन्धित अनुसन्धानमा कुनै कमी वा अन्तरहरू (Gaps) बारे सोचेर केही बताउन सक्नुहुन्छ ?
- हाम्रो यस कुराकानीको क्रममा केहि कुरा छुटेको पाउनु भए कृपया थप्नुहोस?

At the end of the interview

- हामी अन्तर्वार्ताको अन्त्यमा पुग्यौं
- तपाईंको सहभागिताको लागि धन्यवाद
- तपाईंले दिनुभएको जानकारीले नेपालमा सडक सुरक्षाको स्थितिमा सुधार ल्याउन सम्भावित अनुसन्धानको लागि सुची बनाउन मद्दत गर्नेछ।
- हामी तपाईलाई यस अध्ययनको दोस्रो चरणको लागि पनि सम्पर्क गर्नेछौं जसमा हामी अन्तरवार्ताहरूबाट पहिचान गरेका सबै अनुसन्धान प्रश्नहरु प्रस्तुत गर्नेछौं र त्यसमध्ये कुन सबैभन्दा महत्वपुर्ण छ भनेर तपाईंको प्रतिक्रिया लिनेछौं ।
- अन्तिममा तपाईंको केहि प्रश्न छ कि?

स्तम्भ ५. दुर्घटनापश्चातको स्याहार

(To be read to each participant for this pillar)

स्तम्भ ५ ले दुर्घटना पछिको इमरजेन्सीकोलागि प्रतिक्रियामा सुधारको साथै दुर्घटनाका घाइतेलाई उपयुक्त इमरजेन्सी उपचार सेवा एवम् दीर्घकालीन पुनर्स्थापना सेवा दिने स्वास्थ्य र अन्य प्रणालीको क्षमताको सुधारको आवश्यकतालाई सम्बोधन गर्दछ। घाइतेलाई अस्पताल लैजानु अघि गर्नु पर्ने स्याहार प्रणाली, अस्पताल ट्रामा स्याहार प्रणाली, पीडितलाई पुनर्स्थापनाका साथै दीर्घकालीन चिकित्सा सहायता प्रणालीको विकास एवम् सुधार र इमरजेन्सी अवस्थामा सहयोग लिन प्रयोग गरिने एउटै नम्बरको बिकास र सुधारका कृयाकलापहरू दुर्घटना पश्चातको स्याहारका मुख्य बुँदाहरू हुन् ।

Suggested questions and prompts

- तपाई कुन पदमा कार्यरत हुनुहुन्छ र तपाईंको मुख्य जिम्मेवारीहरु के के हुन्?
- तपाई यस पदमा कति समयदेखि हुनुहुन्छ ?

Pant PR, *et al. BMJ Open* 2022; 12:e059312. doi: 10.1136/bmjopen-2021-059312

    - प्रम्टः यस पदका लागि जिम्मेवारी कसरी निर्धारण भएका हुन्छन् ?
- स्तम्भ १ को वर्णनबाट तपाईं को विचारमा नेपालमा यस स्तम्भ अन्तरगत गरिएका कृयाकलापहरूको अवस्था कस्तो छ ?
- तपाईको अनुभवबाट यस स्तम्भ अन्तरगतका कुन कुन कृयाकलापहरू राम्रोसँग अघि बढिरहेका छन् ?
    - प्रम्ट: तपाईंको विचारमा किन यो राम्रो हुँदैछ ?
- तपाईको अनुभवमा यस स्तम्भ अन्तरगतका कुन कामहरू राम्ररी भइरहेका छैन वा राम्रोसँग गर्न सकिएको छैन ?
    - तपाईंको विचारमा किन ती कृयाकलापहरू राम्रोसँग गर्न सकिएको छैन ?
- सडक सुरक्षाको लागि अपेक्षित उद्देश्यहरू प्राप्त गर्न तपाइँले के कस्ता चुनौतीहरू सामना गर्नु परेको छ ?
        - किन देशभरी नै आपत्कालिन सेवा जस्तै एम्बुलेन्स सेवाको लागी एउटै टेलिफोन नम्बर छैन ?
        - तपाईं नेपालका अस्पतालको ट्रमा सेवाहरूलाई कसरी हेर्नुहुन्छ ?
- कुन नयाँ जानकारी वा तथ्य उपलव्ध भइदिए सडक प्रयोगकर्ताहरूको सुरक्षित व्यवहार अवलम्बनलाई सुधार गर्न मद्दत पुग्नेछ?
- के तपाइँ स्तम्भ ५ का यी गतिविधिहरुसंग सम्बन्धित अनुसन्धानमा कुनै कमी वा अन्तरहरू (Gaps) बारे सोचेर केही बताउन सक्नुहुन्छ ?
- हाम्रो यस कुराकानीको क्रममा केहि कुरा छुटेको पाउनु भए कृपया थप्नुहोस?

At the end of the interview

- हामी अन्तर्वार्ताको अन्त्यमा पुग्यौं
- तपाईंको सहभागिताको लागि धन्यवाद
- तपाईंले दिनुभएको जानकारीले नेपालमा सडक सुरक्षाको स्थितिमा सुधार ल्याउन सम्भावित अनुसन्धानको लागि सुची बनाउन मद्दत गर्नेछ।
- हामी तपाईलाई यस अध्ययनको दोस्रो चरणको लागि पनि सम्पर्क गर्नेछौं जसमा हामी अन्तरवार्ताहरूबाट पहिचान गरेका सबै अनुसन्धान प्रश्नहरु प्रस्तुत गर्नेछौं र त्यसमध्ये कुन सबैभन्दा महत्वपुर्ण छ भनेर तपाईंको प्रतिक्रिया लिनेछौं ।
- अन्तिममा तपाईंको केहि प्रश्न छ कि?

Pant PR, *et al. BMJ Open* 2022; 12:e059312. doi: 10.1136/bmjopen-2021-059312
