# Supplementary material for: Identifying research priorities for road safety in Nepal: a Delphi study
Source: BMJ Open. 2022 Apr 13;12(4):e059312. doi: 10.1136/bmjopen-2021-059312 (PMC9014064; doi:10.1136/bmjopen-2021-059312)
Supplement: Author's manuscript [file bmjopen-2021-059312.draft_revisions.pdf]

BMJ Open is committed to open peer review. As part of this commitment we make the peer review history of every article we publish publicly available.

When an article is published we post the peer reviewers' comments and the authors' responses online. We also post the versions of the paper that were used during peer review. These are the versions that the peer review comments apply to.

The versions of the paper that follow are the versions that were submitted during the peer review process. They are not the versions of record or the final published versions. They should not be cited or distributed as the published version of this manuscript.

BMJ Open is an open access journal and the full, final, typeset and author-corrected version of record of the manuscript is available on our site with no access controls, subscription charges or pay-per-view fees (http://bmjopen.bmj.com).

If you have any questions on BMJ Open's open peer review process please email info.bmjopen@bmj.com

BMJ Open

Identifying research priorities for road safety in Nepal: A Delphi study

Journal:	*BMJ Open*
Manuscript ID	bmjopen-2021-059312
Article Type:	Original research
Date Submitted by the Author:	18-Nov-2021
Complete List of Authors:	Pant, Puspa Raj; University of the West of England, Faculty of Health and Applied Sciences<br>Rana, Pranita; Kathmandu Medical College, Nepal Injury Research Centre<br>Pradhan, Kriti; Kathmandu Medical College, Nepal Injury Research Centre<br>Joshi, Sunil Kumar; Kathmandu Medical College and Teaching Hospital, Community Medicine<br>Mytton, Julie; University of the West of England, Faculty of Health and Applied Sciences
Keywords:	Health policy < HEALTH SERVICES ADMINISTRATION & MANAGEMENT, PUBLIC HEALTH, ACCIDENT & EMERGENCY MEDICINE

SCHOLARONE™
Manuscripts

I, the Submitting Author has the right to grant and does grant on behalf of all authors of the Work (as defined in the below author licence), an exclusive licence and/or a non-exclusive licence for contributions from authors who are: i) UK Crown employees; ii) where BMJ has agreed a CC-BY licence shall apply, and/or iii) in accordance with the terms applicable for US Federal Government officers or employees acting as part of their official duties; on a worldwide, perpetual, irrevocable, royalty-free basis to BMJ Publishing Group Ltd ("BMJ") its licensees and where the relevant Journal is co-owned by BMJ to the co-owners of the Journal, to publish the Work in this journal and any other BMJ products and to exploit all rights, as set out in our licence.

The Submitting Author accepts and understands that any supply made under these terms is made by BMJ to the Submitting Author unless you are acting as an employee on behalf of your employer or a postgraduate student of an affiliated institution which is paying any applicable article publishing charge ("APC") for Open Access articles. Where the Submitting Author wishes to make the Work available on an Open Access basis (and intends to pay the relevant APC), the terms of reuse of such Open Access shall be governed by a Creative Commons licence – details of these licences and which Creative Commons licence will apply to this Work are set out in our licence referred to above.

Other than as permitted in any relevant BMJ Author's Self Archiving Policies, I confirm this Work has not been accepted for publication elsewhere, is not being considered for publication elsewhere and does not duplicate material already published. I confirm all authors consent to publication of this Work and authorise the granting of this licence.

For peer review only

Identifying research priorities for road safety in Nepal: A Delphi study

Corresponding author: Dr Puspa Raj Pant[a*]

[a]Faculty of Health and Applied Sciences, University of the West of England, Bristol, BS16 1QY, United Kingdom, Email: pant.puspa@gmail.com ORCiD: https://orcid.org/0000-0002-8827-0018

Co-authors:

Ms Pranita Rana[b], [b]Nepal Injury Research Centre, Kathmandu Medical College Public Ltd, Kathmandu, Nepal.

Ms Kriti Pradhan[b], [b]Nepal Injury Research Centre, Kathmandu Medical College Public Ltd, Kathmandu, Nepal.

Prof Sunil Kumar Joshi[c], [c]Department of Community Medicine, Kathmandu Medical College Public Ltd, Kathmandu, Nepal. ORCiD: https://orcid.org/0000-0002-2704-5060

Prof Julie Mytton[a], [a]Faculty of Health and Applied Sciences, University of the West of England, Bristol, UK. ORCiD: https://orcid.org/0000-0002-0306-4750

Word count: 3,917 (excluding title page, abstract, references, figures and tables.)

Abstract:

Introduction: In low income countries, road traffic injuries cause significant morbidity and mortality and can generate losses of up to 6.5% of gross domestic product. The Safe Systems Approach recognises the essential contribution of different sectors of society to create a transport system that keeps road users safe. Nepal has been a co-sponsor of these principles, but in-country progress has been limited. This study aimed to identify the research needed to help Nepali agencies develop a safe systems approach to road safety. ***Methods:*** We used Delphi methods to develop consensus on a prioritised list of road safety research questions, engaging five groups of stakeholders in three ranking rounds. A final consensus workshop was organised to select which research questions needed answering most urgently. ***Results:*** Out of 133 potential participants identified and contacted, 93 individuals were recruited and took part in 95 interviews covering all five of the World Health Organisation's 'pillars' of road safety. Participants were from a range of organisational and professional backgrounds, including government institutions, academia, road safety engineers, clinicians, civil society organisations, and all had an interest or remit that addressed one or more of the pillars of road safety. Ninety five interviews in round 1 yielded 1019 research suggestions. The

For peer review only - http://bmjopen.bmj.com/site/about/guidelines.xhtml

ranking rounds and workshop ultimately led to the selection of 6 prioritised and urgent questions.

Conclusions: The application of Delphi approach is useful when there is lack of evidence and participants representing a range of institutions and expertise to road safety pillars provide research priorities based on consensus. Outcomes from this study provide Nepali researchers with a greater understanding of the focus for future road safety research.

Keywords:

Safer Road users, Road Traffic Injuries, Road Safety Pillars, Post-crash Response, Delphi.

Strengths and limitations

- Ninety-three Nepali experts participated; bring perspectives from road construction, vehicle management, transport management and post-crash response.
- The research questions identified were ranked by the participants individually as well as discussed during group meetings to achieve consensus.
- The Delphi approach is at risk of high dropout of participants; we were able to retain a high proportion of participants through the study.

INTRODUCTION

Globally road traffic injuries are increasing, with an estimated 1.35 million deaths and up to 50 million non-fatal injuries in 2016. [1] Despite having only 1% of the world's vehicles, low-income countries have 13% of fatal road traffic injuries. Road traffic injuries are the leading cause of death for children and young adults between 5-29 years globally, and are an important cause of disability and poverty. RTIs have been estimated to generate losses of up to 6.5% of a low-income country's gross domestic product. [2]

The WHO World Report on Road Traffic Injury Prevention, [3] subsequent Road Safety Status Reports [1] and the WHO Save LIVES technical package of 22 evidence-based interventions [4] argue for a "safe systems approach" [5] to reduce road dangers and the numbers of people killed and seriously injured on the roads. This approach recognises the essential contribution of different sectors to create a system that keeps road users safe. The World Health Organization published the Global Plan of Action for Road Safety 2011-2020 [6] alongside the United Nations and this plan of action recommended five 'pillars'; road safety management (Pillar 1), safer roads and mobility (Pillar 2), safer vehicles (Pillar 3), safer road users (Pillar 4) and post-crash response (Pillar 5). Action across all

For peer review only

five pillars can contribute to reduced road traffic injuries. Nepal has been a co-sponsor to these principles, but progress has been limited.

A huge road construction programme in Nepal has seen over 15,000 km of new blacktop, gravel and earthen roads built by federal, provincial and local governments in the last 5 years [7] and there are plans to have a total of 13,500 km blacktopped road by 2023/24. [8] Many new roads do not have proven safety features and are poorly maintained. The roads in the hills are considered to be dangerous because of landslides in addition to frequent road crashes due to poor engineering or poor safety infrastructure. [9] The Department of Transport Management in the Government of Nepal produces vehicle registration statistics that show more than half (53%) of the 3.22 million motorised vehicles in Nepal were registered in the 5 years to July 2018 and about 78% of total registered vehicles were motorcycles. [10]

Nepal lacks a funded road safety implementation plan, a national ambulance service or globally recognised vehicle standards. The national helmet wearing law is not enforced for motorcycle passengers and there is no legislation for passenger seatbelt use, child restraints or mobile phone use whilst driving. Data are limited and of poor quality; WHO estimates of road traffic fatalities in Nepal in 2016 (4,622) are more than double those recorded by the Traffic Police (2,006), and there are no routinely published estimates of deaths by road user category available. [1] Nepal's Health Management Information System recorded over 100,000 hospital visits for the treatment of orthopaedic problems secondary to road traffic events in the year 2017/18 indicating the significant burden of road traffic injuries on health systems. [11] Road traffic crashes and injuries in Nepal are rising despite existing legislation. [12 13] Tackling road traffic injuries is a priority in the government's Health Sector Strategy 2015-2020. [14] A National Road Safety Action Plan 2013-2020 [15] was acknowledged but not ratified by Parliament. Neither document specified the research required to support the delivery of improved road safety.

In order to improve road safety, coordinated efforts are needed across the road transport system. Research is vital to optimise decision-making. Current initiatives in Nepal for the control and prevention of road traffic crashes and their consequences are not based on local evidence. Therefore, this study aimed to identify the research needed to help agencies in Nepal develop a safe systems approach to road safety, and to achieve a consensus about which studies should be prioritised.

For peer review only - http://bmjopen.bmj.com/site/about/guidelines.xhtml

METHODS

This study used the Delphi approach [16-18] to develop a consensus on a prioritised list of road safety research questions. Five groups of stakeholders in Nepal were engaged. The roles and experience of participants were relevant to each of the five WHO pillars of road safety. The study was conducted in two stages: firstly, interviews were conducted with stakeholders to identify a range of possible research questions, and secondly, participants completed two rounds of ranking the research questions in order of importance. Each of the five road safety pillars was studied separately. Five interview topic guides were developed in the Nepali language, based on the activities recommended for each of the five WHO pillars of road safety (Supplementary file 1).

Recruitment of the participants

Potential study participants were identified through existing networks and multi-sector stakeholder groups on road safety and first response. Networks included third sector and advocacy organisations for road safety. Participants helped identify further potential participants through a snowballing approach. We aimed to recruit 20-25 participants for each of the five pillars. Potential participants were contacted by telephone and were provided with information about the study and their interest in our research was confirmed. For participants expressing an interest, written information regarding the study and a consent form were sent to the potential participants via email. All the recruitment took place during the Covid-19 pandemic and therefore most of the interviews were completed remotely, by phone or videocall. For these participants, consent was recorded verbally at the start of the interview, or was collected prior to participation via email. Later in the pandemic it became feasible to engage some participants face-to-face. For these participants consent was collected at this meeting.

Data collection and analysis

In Round 1, we conducted interviews with participants in which we asked what additional data or information would help them in their job and reduce road traffic injuries. We explored the barriers they faced when tackling road safety. Most of the interviews were conducted using online platforms such as MS Teams, Zoom, Google Meet or Viber, and some interviews were conducted over the telephone. Towards the end of the data collection period, and when Covid-19 pandemic restrictions allowed, we conducted a small number of face-to-face interviews where this was the preference of the participants. In these circumstances, mitigations against infection, such as social distancing and the wearing of face masks, helped protect both participants and researchers. Interviews were conducted in Nepali language and audio-recorded. Audio recordings were listened to several times.

For peer review only

For peer review only - http://bmjopen.bmj.com/site/about/guidelines.xhtml

Information relating to perceived gaps in research or evidence was documented as potential research questions on a spreadsheet, in English. For each group of stakeholders, approximately 200 research suggestions were generated from the interviews. Many of the participants raised similar issues, therefore it was possible to cluster the questions into groups, and to formulate a single question to represent that area of research need. The grouping stage was completed collaboratively by the whole research team to ensure that questions were treated equally and the process consistently applied. A reduced list of about 30 questions was achieved, identifying the research and evidence needs relating to each pillar of road safety.

For Round 2, the research questions from the reduced list were uploaded to an online survey tool (Qualtrics) in both English and Nepali languages. The link to the survey was distributed to the participants via email or Viber message. Participants were asked to give their opinion on the importance of each research question using a 5-point Likert scale: *Not Important*, *Low Importance*, *Moderate Importance*, *Important*, and *Most Important*. Reminders to complete the survey were sent via email and individual phone calls after one week and followed up again 2-3 days later. Completed surveys were exported from Qualtrics and analysed in MS Excel. Survey results were collated to identify the number of participants who rated each question as "very important" or "important". Questions where a significant majority of participants had scored them 'important' or 'very important' were retained as prioritised questions. The threshold for retention as a prioritised question was set at 70% for participants representing Pillars 1, 3, 4 and 5 and at 80% for Pillar 2 (where a greater proportion of questions were considered important). We used these threshold values based on published Delphi studies. [19 20]

For Round 3, participants were invited to a real-time online workshop where the prioritised questions were presented and discussed. The workshop was designed to provide the participants the opportunity to share their views and listen to each other's opinions regarding which issues were the most important to research. These workshops were recorded and shared with those who were not able to join. Following the workshop, a Qualtrics survey was sent to all participants again, this time listing only those questions prioritised from Round 2. Participants were again asked to score each question as either *Not Important*, *Low Importance*, *Moderate Importance*, *Important*, or *Most Important*. Reminders were sent to the participants after one week and followed up again after 2-3 days. Completed surveys were exported to MS Excel and collated to identify the number of participants considering each question 'important' or 'very important'. This resulted in the final prioritised list of research questions for each pillar of road safety.

For peer review only - http://bmjopen.bmj.com/site/about/guidelines.xhtml

The research team completed Rounds 1, 2 and 3 for one pillar before moving on to the next pillar. The interviews started on 12 July 2020 and were completed on 14 February 2021. Due to the Covid-19 pandemic, where government officials and clinical staff were not easily available to participate, stakeholders in Pillars 1 and 5 were left until later in the study when the peak of the first wave of Covid-19 in Nepal had passed.

Overarching consensus workshop

A final online consensus workshop was organised where the top ranked research questions from all five pillars were shared with all the participants, stakeholders from our advisory groups and invited key decision makers. A facilitated discussion explored the understanding of what the different research options could provide and how that new evidence could potentially be used. Using online voting software (Mentimeter, https://www.menti.com), participants were encouraged to vote for one research question from each pillar that they considered needed to be addressed the most urgently. The questions considered most urgent were presented back to the group.

Ethical approval for conducting this study was obtained from the Kathmandu Medical College Institutional Review Committee (ref. 040620201) and the University of the West of England Bristol Faculty Research Ethics Committee (ref. HAS. 20.06.192).

Patient and public involvement

Through community engagement and involvement we engaged individuals with diverse views on road safety, ranging from road users through to those with decision making authority for road development, management and traffic regulation.

RESULTS

Description of study participants

Out of 133 potential participants identified and contacted, 93 individuals were recruited and took part in 95 interviews covering all five road safety pillars (two participants had expertise relevant to more than one pillar, and therefore took part in two interviews each). Participants were from a range of organisational and professional backgrounds, including government institutions, academia, road safety engineers, clinicians, civil society organisations, and all had an interest or remit that addressed one or more of the five pillars of road safety. Some of the experts in our list, when contacted, suggested the name of other stakeholders. Out of 93 participants, 83 were from Kathmandu valley,

For peer review only - http://bmjopen.bmj.com/site/about/guidelines.xhtml

and 10 were from outside Kathmandu. The participants' background characteristics are summarised in Table 1.

Table 1. Organisational/professional background of the participants

Organisational / professional background	Total	Male	Female
Government organisation (Secretaries, Govt Officers, Police, Political representatives)	33	30	3
Clinician, Nurse, physiotherapist	10	8	2
Road safety Engineer	9	9	0
Road Safety Advocacy	8	5	3
Academics	7	6	1
First Aid/ emergency/ ambulance provider	6	6	0
Engineers' Association	4	4	0
Transport worker	4	4	0
Automobile dealer	3	3	0
Federation of transport	2	2	0
Schools' organisation	2	2	0
Sustainable Transport	2	2	0
Others (journalist and city planners)	3	3	0
**Total**	**93**	**84**	**9**

Across all five pillars we identified a total of 1019 research suggestions from the 95 interviews completed in Round 1. Collating similar questions reduced this to 141 questions across the five pillars. Seventy-six (80%) participants took part in Round 2, through which the list of questions was reduced to 91 questions. Forty (43%) participants took part in an online workshop prior to further ranking in Round 3 which was completed by 64 (67%) participants and resulted in a total of 30 prioritised questions. Figure 1 shows the stages of the Delphi study and the number of participants in each round. Attrition of participants was greatest for the group discussing Pillar 1 (road safety management), where 10/21 (48% participants) dropped out between Round 1 and Round 3. Attrition was least in the group discussing Pillar 2 (safer roads) where only 3/18 (17%) of participants were lost.

Figure 1. Flow chart of the Delphi process

For peer review only - http://bmjopen.bmj.com/site/about/guidelines.xhtml

The high attrition of participants in Pillar 1 was not unexpected since many of these participants worked in government positions and it was difficult for them to prioritise attendance during the Covid-19 pandemic. Figure 2 illustrates participant attrition throughout the study.

Figure 2. Study participants retained in subsequent Delphi rounds, by pillar.

Table 2 describes the number of research questions prioritised in each Round, split by the pillars of road safety. The retention rate in this study was equivalent to that in other published Delphi studies [21] despite the Covid-19 pandemic.

Table 2. Research questions prioritised at each Round, by pillar

Pillar of road safety	Round 1				Round 2	Round 3
	Interview dates	Number of interviews (online or by phone)	Research questions generated ('long list')	Grouped research questions ('reduced list')	Number of 'Important' or 'very important' research questions (above 70% consensus)	Number of questions considered most important (Top 5 ranks)
Pillar 1 (Road safety management)	23 Nov to 22 Jan 2021	21 (21)	183	25	17	5
Pillar 2 (Safer roads and mobility)	13 July to 12 Aug 2020	18 (4)	211	30	19*	5
Pillar 3 (Safer vehicles)	16 Aug to 15 Sep 2020	17 (17)	217	30	20	7
Pillar 4 (Safer road users)	23 Sept to 19 Oct 2020	20 (20)	178	30	20	6

For peer review only - http://bmjopen.bmj.com/site/about/guidelines.xhtml

Pillar 5 (Post-crash response)	05 Jan to 14 Feb 2021	19 (13)	230	26	15	7
Total		95 (75)	1019	141	91	30

*80% consensus

The top ranked research questions for the five pillars of road safety are presented in Table 3. The research questions that were considered the most important cover a wide range of issues, including how to make existing processes more effective, how to assess the training needs of the road safety workforce, understanding the challenges of implementing existing road safety legislation, how to improve accountability for road safety, how to generate and disseminate better information to inform decisions, and how to generate evidence that supports the economic argument for road safety.

Table 3 List of top questions for Pillars 1 to 5 with scores in rounds 2 and 3

	Scores*	
**Pillar 1: Road Safety Management**	**R2**	**R3**
How can implementing agencies be made more accountable for road safety in urban and rural areas?	92	91
What are the barriers to conduct road safety audits at all stages of road construction and implementation of its recommendations?	85	91
How can urban and rural roads construction and management be governed to ensure improved road safety?	92	91
How can the traffic management system be improved to ensure it improves the safety of all road users?	85	91
What are the barriers to implementation of existing laws related to road safety in Nepal?	69	91
**Pillar 2: Safer Roads**		
What is the effectiveness of different safety features installed on roads in terms of crash reduction?	94	100
What are the barriers and facilitators for achieving safer roads in Nepal?	88	100
What kind of institutional setup is needed at central, provincial and local levels for the promotion of road safety ownership and accountability?	94	93
What are the economic benefits of installation of safety features during road construction, regular maintenance and upgrading of roads?	82	93

For peer review only - http://bmjopen.bmj.com/site/about/guidelines.xhtml

How should different types of roads, and roads in different geographical locations, be designed to make them safer for all road users?	82	93
**Pillar 3: Safer vehicles**		
What are the factors affecting fitness condition and road worthiness of vehicles to the extent that it leads to road traffic crash?	86	100
What should be the minimum criteria for the establishment of standard vehicular maintenance workshops?	93	92
What are the needs for the capacity development and training for currently working human resources and additional jobs to improve the safety of vehicles in Nepal?	71	92
What improvements in policies and institutional setup is needed to ensure vehicles safety of all types and routes?	79	92
What is the role of motor parts used for vehicle maintenance for fitness condition of the vehicles and road crashes?	93	83
How does overloading impact safety of the vehicles?	71	83
What are the vehicle related factors causing road crashes in Nepal?	71	83
**Pillar 4: Safer Road users**		
How can the driver licencing system be made more effective in order to ensure safer vehicle drivers?	100	93
What are the main factors increasing the risk of public vehicle crashes? What interventions would improve the safety of travel on public vehicles?	94	93
How can licensing and crash data collection systems be improved?	94	93
What are the major causes of road crashes in Nepal? What percentage of road crash is due to unsafe road user behaviours?	94	87
What content should be included in awareness campaigns for different types of road user, and how are these campaigns best delivered?	83	87
What are the barriers in the implementation of laws regarding safer road user behaviour? Review of existing policies related to safer road users.	78	87
**Pillar 5: Post-crash response**		
What standards should be applied to ambulance services? (includes standards for personnel and training, equipment carried, and the vehicles)	100	100
What is the standard of care at health centres and hospitals for road traffic injury patients across the country, and how can they be improved?	79	92

For peer review only

For peer review only - http://bmjopen.bmj.com/site/about/guidelines.xhtml

What is the current average time taken for a road traffic injury patient to receive first response at the scene and the average time taken to arrive at a healthcare setting able to meet their care needs? How can any delays be reduced?	93	92
What factors influence the ability of the post-crash emergency response service to get to the patient and then get them to the right hospital in the best possible time?	86	92
What should be included in the training curriculum for the different levels of post-crash responders?	93	85
How should policies and legislation be further developed to support the post-crash response for road traffic injury victims?	71	85
What is the optimal model of insurance to minimise death & disability following a road traffic crash? What are the barriers & facilitators to implementing such an insurance system?	71	85

Note: Percent of participants ranked "very important" or "important"; R2= Round 2; R3= Round 3.

A total of 56 people attended the workshop conducted at the end of the study where the list of the top ranked research questions for each of the five pillars were presented. Using electronic voting software to identify the question within each Pillar considered to be the most urgent, 6 questions were prioritised. Two questions in Pillar 4 were scored equally (Table 4).

Table 4. Top 6 most urgent research questions

Pillars	Research Questions
Pillar 1	How can implementing agencies be made more accountable for road safety in urban and rural areas?
Pillar 2	How should different types of roads, and roads in different geographical locations, be designed to make them safer for all road users?
Pillar 3	What are the factors affecting fitness condition and road worthiness of vehicles to the extent that it leads to road traffic crashes?
Pillar 4	How can the driver licensing system be made more effective in order to ensure safer vehicle drivers?

For peer review only

For peer review only - http://bmjopen.bmj.com/site/about/guidelines.xhtml

	What are the main factors increasing the risk of public vehicle crashes? What interventions would improve the safety of travel on public vehicles?
Pillar 5	What factors influence the ability of the post-crash emergency response service to get to the patient and then get them to the right hospital in the best possible time?

DISCUSSION

This study is the first to our knowledge that has engaged such a wide group of participants to identify the research priorities relevant to the improvement of road safety in Nepal. The research team was able to identify and approached 133 potential participants and 70% (93 people) agreed to take part. Respondents included stakeholders from a range of organisational and professional backgrounds as well as geographical areas and included; officials in government institutions (Secretaries, Government Officers, Police, Political representatives), clinicians, nurses, physiotherapists, engineers, academics, first responders, transport workers, automobile dealers, road users, members of the media and city planners. The number of participants that should take part in a Delphi study is not prescribed and it can be anywhere above 10 persons; the number is guided by the scope of the problem and existing resources. [19 20] Overall, the retention of the participants until the third round of ranking was excellent however, rates varied between different pillars. The overall retention rate of 70% and 50% attendance at the final consensus workshop indicated the high level of interest in road safety research in Nepal. This rate is higher than that reported by Marchau and Van der Heijden [22] in a multi-country road safety study. Marchau and Van der Heijden [22] applied the Delphi technique to explore the policy aspects of implementing driver support systems. The authors used a questionnaire with specified answer options sent to international experts from USA, Japan and Europe. In this study, 56% (65 out of 117) responded in the first round while only 40 responded in the third round. Compared to these rates of participation our study was well attended.

Road safety research is a neglected issue in low- and middle- income countries [23] and lack of research capacity may be one of the reasons. In Nepal, a policy review identified that institutional arrangements and resource allocation for road safety were inadequate. [24] The lack of coordination of road safety sectors is a challenge globally [25 26] as well as in Nepal. Many of the participants in this study had the opportunity to meet and discuss road safety with those working in other sectors, for the first time.

For peer review only

Other studies exploring aspects of road safety through the use of the Delphi technique have mostly come from high income countries, with the exception of a few, such as Vietnam and Uganda. Studies have explored specific risk factors such as cell phone use and sleep deprivation in the USA, [27 28] and public bus safety in Italy. [29] Some studies focussed on the need to improve post-crash care such as; strengthening trauma management in Vietnam, [30] pre-hospital emergency care in Iran, [31] post-recovery rehabilitation in Australia, [32] and emergency medical services capacity in Uganda. [33] In Iran, Delphi studies have been conducted to inform the development of minimum datasets to study road crashes, [34] and developing a national road safety education programme. [35] We have not identified any previously published Delphi studies that have included all five pillars of road safety in a single study.

Zhu, et al. [27] recruited road safety experts and young drivers in the USA to study the risks of mobile phone use while driving. Expert participants identified texting, sending email or picking up phone as particularly high risk behaviours for crashes, but not playing music on a handheld mobile which was prioritised by young drivers. Participants identified 20 behavioural practices related to mobile use which can result in a collision. Our study participants (Pillar 4) also identified the importance of studying causes of driver distraction, but did not identify mobile phone use in particular.

Cafiso, et al. [29] engaged the managers of large public bus companies in Italy in a Delphi study to explore bus safety. Participants rated safety solutions for issues relating to driver behaviour, traffic conflicts and vehicle maintenance and technology. Driver inattention and fatigue were considered major causes of bus crashes. Technology to control when the bus can start, automatic door closing and the materials used inside the bus were priorities for keeping passengers safe. An expert panel on sleep deprivation in a study by Czeisler, et al. [28], agreed that a driver was not fit to drive if they had less than 2 hours sleep in the previous 24 hours. These studies illustrate how previous Delphi studies have tended to focus on specific road safety issues, and how the results are specific to the context or participants. Neither would be directly generalizable to Nepal, nor do they cover the breadth of safety issues in our study.

Several Delphi studies have reported post-crash trauma management and prehospital care. In Vietnam, Schmucker, et al. [30] used online meetings followed by a questionnaire survey of 1000 road users to generate responses that were ranked and outcomes were used to inform the development of a trauma care course. Recently, Azami-Aghdash, et al. [31] used the Delphi technique to achieve a consensus about 37 indicators to measure and improve the performance of prehospital care following road crashes in Iran. This is similar to the topic prioritised for post-crash response (Pillar 5)

For peer review only - http://bmjopen.bmj.com/site/about/guidelines.xhtml

in our study. However, the differences in Iranian and Nepali country contexts and pre-hospital care infrastructure may mean that performance indicators in Iran are not generalizable to Nepal. Balikuddembe, et al. [33] used the Delphi technique to identify and prioritise factors affecting the exposure, vulnerability and emergency medical service capacity for the victims of road traffic injuries in Kampala. They identified 23 of factors that affect Emergency Medical Service capacity which were relevant to Pillar 5 findings of our study because these factors were addressed the entire system rather than victim-specific needs for trauma management.

In the course of our study, shifts in the opinions of participants were observed during Rounds 2 and 3. In relation to the rankings completed in Round 2, a high degree of consensus was observed and the process of creating a reduced list for Round 3 was relatively straightforward. The Delphi method dictates that the results of a first round be re-presented to participants in subsequent Rounds, giving participants the opportunity to reconsider their views in the light of discussion, additional thought and/or the results obtained from other participants. [20 36] Cafiso, et al. [29] in their study, similarly reported that after the second Round, the Delphi panellists' opinions were influenced by those of their colleagues. The participants in the current study also exhibited further changes of opinion in Round 3. Although participants were asked to rank the research questions for importance in both ranking rounds, the changed ranks of the questions in each Round illustrate the value and influence of discussion between Rounds in reaching a consensus view.

High numbers of research questions were rated 'important' or 'very important' in our study, illustrating that many participants recognised the need for road safety research in Nepal. The Government of Nepal plans to enact a Road Safety Bill [37] that will include issues relating to planning, resourcing, implementation, and evaluation of national road safety activities. Provincial Governments, which were established only 4 years ago, through the promulgation of the constitution of Nepal, [38] have started to enact Provincial Transport Management Acts. However, the institutional structures necessary to implement these laws are still in development. [24] The research questions prioritised in this study emphasise the need for evidence to support both national development plans [8] and safer roads and transport in Nepal. [39] Existing road safety policies are mostly only partially implemented. [24] Policy gaps include policies to separate traffic and road users and those to address speed management.

Strengths and limitations

For peer review only - http://bmjopen.bmj.com/site/about/guidelines.xhtml

Representation and involvement of most of the individuals and experts currently active in the fields of road construction, vehicle management, transport management and post-crash response is a major strength of this study. The Delphi method for achieving consensus is a research technique with the potential for biases; [20] Hallowell [17] outlined common biases in implementation and here we describe the measures applied to minimise these biases in the current study. To minimize factors that might influence the quality of the conclusions due to the level of expertise of the panel members, [40] only experienced and recognised authorities working for road safety in Nepal were invited to participate. The results produced by Delphi studies are often thought to be limited due to poor quality of the facilitator's survey instruments, [16] therefore, the tools developed for this study were informed by the international literature and advice was available from an experienced Delphi expert. Bias can occur if questions are poorly worded [17] therefore our researchers were trained in interviewing skills prior to commencing Round 1 and conducted the interview in Nepali. Some critics believe that convergence of opinion in Delphi studies is conformity. [18] To counter this risk, we synthesised best global road safety practice as reported in published literature and presented this to participants during the workshops between Rounds 2 and 3. Although the Delphi approach has been reported to be overly time intensive, [41] we found the time taken to participate did not inhibit a high proportion of participants to remain in the study to its conclusion.

CONCLUSIONS

This study identified research priorities for road safety in Nepal across all the WHO's five pillars of road safety. It can provide a guide for researchers when designing future studies and has provided opportunities for stakeholders across sectors in Nepal to meet and debate issues together. Future research has the potential to lead to evidence-informed policy development and implementation, and improved practices relating to road construction and management, vehicle standards, and post-crash care, making the roads safer for all road users in Nepal.

Acknowledgements: We would like to acknowledge the support of Professor Nichola Rumsey who provided training in Delphi study methodology to the study team, advised on the study protocol and this manuscript. We are grateful to all 93 expert participants without whom this study would not have been possible.

Contributors: Puspa Raj Pant: Data curation; Project administration; Original draft. Pranita Rana: Data collection; Investigation; Project administration; Validation; review & editing. Kriti Pradhan:

For peer review only - http://bmjopen.bmj.com/site/about/guidelines.xhtml

Data collection; Investigation; Project administration; Validation; review & editing. Sunil Kumar Joshi: Project administration; Resources; Supervision; review & editing. Julie Mytton: Conceptualization; Data curation; Funding acquisition; Methodology; Supervision; review & editing.

Funding: This research was commissioned by the National Institute for Health Research (NIHR) Global Health Policy and Systems Research Development Award using UK aid from the UK Government (NIHR129877). The views expressed in this publication are those of the author(s) and not necessarily those of the NIHR or the Department of Health and Social Care.

Competing interest: the authors have no competing interests to declare

Data availability statement: There are no additional unpublished data

References:

1. World Health Organization. Global status report on road safety 2018: World Health Organization 2018.
2. World Bank. Guide for Road Safety Opportunities and Challenges: Low- and Middle-Income Countries Country Profiles. . Washington DC, USA: World Bank, 2019.
3. Peden M, Scurfield R, Sleet D, et al. World report on road traffic injury prevention: World Health Organization Geneva, 2004.
4. WHO. Save LIVES - A road safety technical package. Geneva: World Health Organization, 2017.
5. World Road Association. The Safe System Approach. In: Kienreich R, Milton J, eds. Road Safety Manual: A manual for practitioners and decision makers on implementing safe system infrastructure: World Road Association (PIARC), 2019.
6. WHO. Global plan for the decade of action for road safety 2011-2020. Geneva: World Health Organization, 2011.
7. Ministry of Finance. Economic Survey 2020/21. Ministry of Finance, Kathmandu, 2021.
8. National Planning Commission. The fifteenth plan (FY 2019/20 to 2023/24) unofficial English translation. Kathmandu: Government of Nepal, 2020.
9. McAdoo BG, Quak M, Gnyawali KR, et al. Roads and landslides in Nepal: how development affects environmental risk. *Natural Hazards and Earth System Sciences* 2018;18(12):3203-10.
10. Department of Transport Management. Statistical details of vehicles registered in Nepal up to FY 2017/18 [Online]. Kathmandu2020 [Statstics of registered vehicles in Nepal]. Available from:

For peer review only - http://bmjopen.bmj.com/site/about/guidelines.xhtml

https://www.dotm.gov.np/Files/NoticePDF/vehiclesdatatill2074-752020-01-17_04-49-13-732.pdf accessed 24 May 2021.

11. Ministry of Health and Population. HMIS Database 2074/75 by Local Government Kathmandu: Department of Health Services; 2021 [updated 25 March 2020. Available from: https://dohs.gov.np/ihims-raw-data/ accessed 1 April 2021.

12. Ojha KN. Road safety status and some initiatives in Nepal. *ITEGAM-JETIA* 2021;7(27):20-40.

13. Pant PR, Mytton J, Dharel MR, et al. The prevention of–and first response to–injuries in Nepal: a review of policies and legislation. *Health research policy and systems* 2021;19(1):1-20.

14. Ministry of Health and Population. Nepal Health Sector Strategy 2015-2020 Kathmandu: Ministry of Health and Population, Government of Nepal, 2015.

15. Goverment of Nepal. Nepal Road Safety Action Plan (2013 - 2020): Draft. Kathmandu, Nepal: Department of Road, Goverment of Nepal, 2011.

16. Gupta UG, Clarke RE. Theory and applications of the Delphi technique: A bibliography (1975–1994). *Technological Forecasting and Social Change* 1996;53(2):185-211. doi: https://doi.org/10.1016/S0040-1625(96)00094-7

17. Techniques to minimize bias when using the Delphi method to quantify construction safety and health risks. Construction Research Congress 2009: Building a Sustainable Future; 2009.

18. Rowe G, Wright G. The Delphi technique: Past, present, and future prospects—Introduction to the special issue. *Technological forecasting and social change* 2011;78(9):1487-90.

19. Hasson F, Keeney S, McKenna H. Research guidelines for the Delphi survey technique. *Journal of advanced nursing* 2000;32(4):1008-15.

20. Powell C. The Delphi technique: myths and realities. *Journal of Advanced Nursing* 2003;41(4):376-82. doi: https://doi.org/10.1046/j.1365-2648.2003.02537.x

21. Hamlet C, Rumsey N, Williamson H, et al. Consensus research priorities for facial palsy: A Delphi survey of patients, carers, clinicians and researchers. *Journal of Plastic, Reconstructive & Aesthetic Surgery* 2018;71(12):1777-84.

22. Marchau V, Van der Heijden R. Policy aspects of driver support systems implementation: results of an international Delphi study. *Transport Policy* 1998;5(4):249-58.

23. Perel P, Ker K, Ivers R, et al. Road safety in low-and middle-income countries: a neglected research area. *Injury Prevention* 2007;13(4):227-27.

24. Pant PR, Mytton J, Dharel MR, et al. The prevention of – and first response to – injuries in Nepal: a review of policies and legislation. *Health Research Policy and Systems* 2021;19(1):65. doi: 10.1186/s12961-021-00686-1

For peer review only - http://bmjopen.bmj.com/site/about/guidelines.xhtml

25. Bliss T, Breen J. Meeting the management challenges of the Decade of Action for Road Safety. *IATSS research* 2012;35(2):48-55.

26. Torbaghan ME, Sasidharan M, Burrow M, et al. Lessons from road safety research and policy engagement in Pakistan, Nepal, Tanzania. 2019

27. Zhu M, Rudisill TM, Rauscher KJ, et al. Risk perceptions of cellphone use while driving: Results from a delphi survey. *International journal of environmental research and public health* 2018;15(6):1074.

28. Czeisler CA, Wickwire EM, Barger LK, et al. Sleep-deprived motor vehicle operators are unfit to drive: a multidisciplinary expert consensus statement on drowsy driving. *Sleep health* 2016;2(2):94-99.

29. Cafiso S, Di Graziano A, Pappalardo G. Using the Delphi method to evaluate opinions of public transport managers on bus safety. *Safety science* 2013;57:254-63.

30. Schmucker U, Ottersbach C, Frank M, et al. A new approach and first steps to strengthen trauma management and road safety in North Vietnam. *Journal of trauma management & outcomes* 2008;2(1):1-7.

31. Azami-Aghdash S, Moosavi A, Gharaee H, et al. Development of quality indicators to measure pre-hospital emergency medical services for road traffic injury. *BMC health services research* 2021;21(1):1-12.

32. Smits EJ, Gane EM, Brakenridge CL, et al. Expert consensus and perspectives on recovery following road traffic crashes: a Delphi study. *Disability and rehabilitation* 2020:1-10.

33. Balikuddembe JK, Ardalan A, Khorasani-Zavareh D, et al. Factors affecting the exposure, vulnerability and emergency medical service capacity for victims of road traffic incidents in Kampala Metropolitan Area: a Delphi study. *BMC emergency medicine* 2016;17(1):1-8.

34. Mohammadi A, Ahmadi M, Gharagozlu A. Developing a minimum data set for an information management system to study traffic accidents in Iran. *Iranian Red Crescent Medical Journal* 2016;18(3)

35. Bakhtari-Aghdam F, Sadeghi-Bazargani H, Azami-Aghdash S, et al. Developing a national road traffic safety education program in Iran. *BMC public health* 2020;20(1):1-13.

36. Williamson K. The Delphi Method. Research methods for students, academics and professionals: Information management and systems. Second ed. New South Wales: Kirsty Williamson 2002.

37. RSS Nepal. Call to pass bill related to road safety. *The Himalayan Times* 2019 3 Feb 2019.

38. Government of Nepal. The Constitution of Nepal. Kathmandu, 2015.

For peer review only

For peer review only - http://bmjopen.bmj.com/site/about/guidelines.xhtml

39. Government of Nepal. Vehicle and Transportation Management Act 1993 (Ammendment 2019). Kathmandu, 1993.

40. Veltri AT. Expected use of management principles for safety function management. West Virginia University, 1985.

41. Rajendran S. Sustainable construction safety and health rating system. 2006

For peer review only

Legends

Figure 1. Flow chart of the Delphi process

Figure 2. Study participants retained in subsequent Delphi rounds, by pillar.

For peer review only - http://bmjopen.bmj.com/site/about/guidelines.xhtml

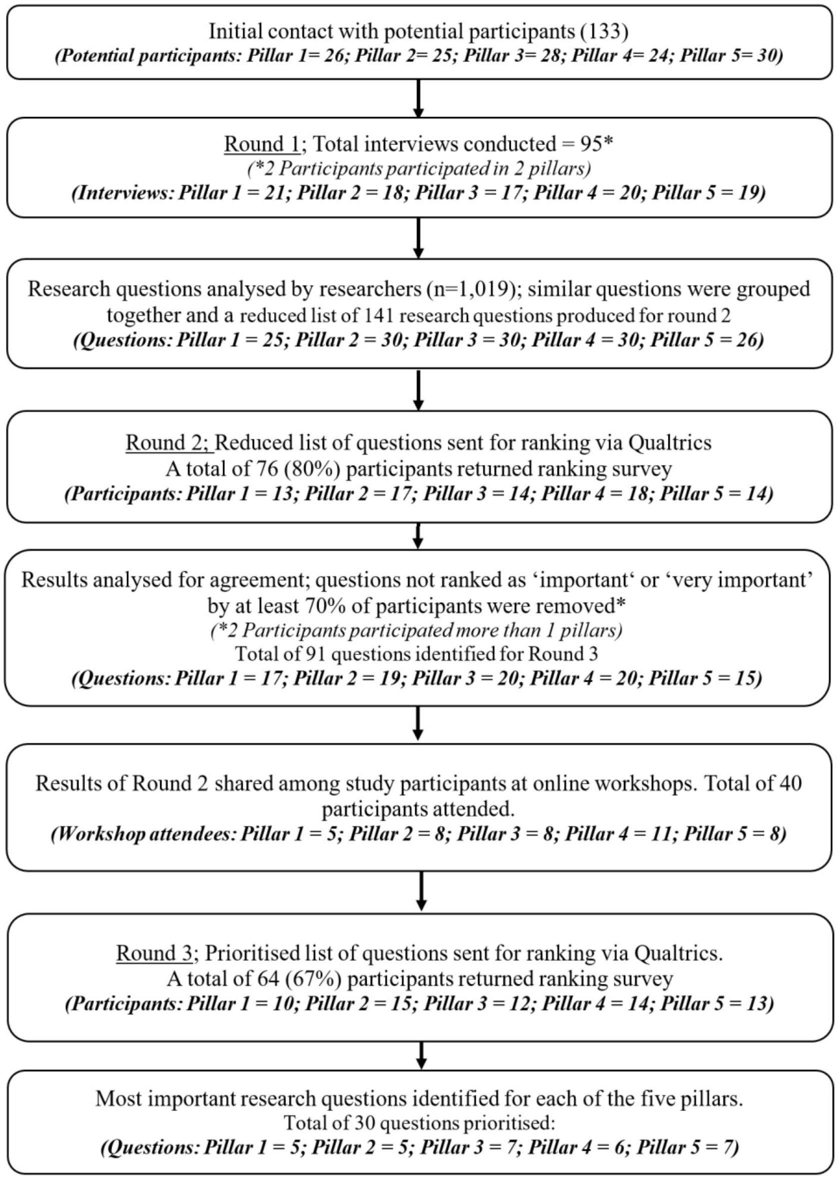

Figure 1. Flow chart of the Delphi process

376x535mm (96 x 96 DPI)

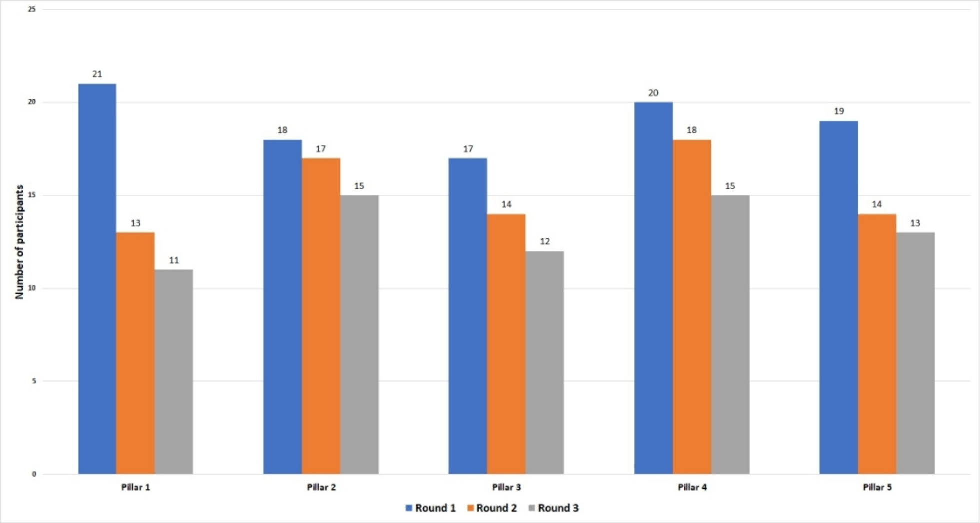

Figure 2. Study participants retained in subsequent Delphi rounds, by pillar.

420x226mm (96 x 96 DPI)

Interview guide

Road Safety Research Prioritisation study

Pillar 1: Road safety management

(To be read to each participant for this pillar) *This pillar focuses on strengthening multi-agency capacity for road safety. It includes activities such as putting into practice major UN road safety conventions, establishing a multi-sectoral national agency to lead road safety activities, developing a national road safety strategy and setting realistic and long-term targets for related activities with sufficient funding for implementation. It also calls for the development of data systems to effectively monitor and evaluate activities.*

Questions and prompts

- What is your job title and what is the focus of your responsibilities for this position?
- How long have you been in this role?
  - Prompt: How these responsibilities are determined?
- From the description of Pillar 1, what is the status of activities for this pillar in Nepal?
- From your experience, what is going well?
  - *Prompt: Why do you think it is going well?*
- From you experience what has not yet happened or is not working well?
  - *Prompt: Why do you think it is not working well?*
- What challenges are you facing to achieve your desired objectives for road safety?
  - *Prompts:*
    - *challenges regarding to have a lead agency?*
    - *challenges regarding national strategy?*
    - *challenges regarding data generation?*
    - *challenges regarding monitoring and evaluation?*
- What information or evidence do you think would help you achieve these objectives?
- Can you think of any gaps in the research available to you, related to these activities in pillar 1?
- Would you like to add anything which we have not covered during this conversation?

At the end of the interview

- Explain that this is the end of the interview.
- Thank the participant for their time.
- Explain that the information they have given will be used to create a list of possible research ideas to improve road safety in Nepal.
- Explain that they will be invited to the next stage of the study where they will hear all of the research questions we have identified, and they will be invited to tell us which ones they think are the most important.
- Ask if they have any questions before you go.

For peer review only - http://bmjopen.bmj.com/site/about/guidelines.xhtml

Pillar 2. Safer roads and mobility

(To be read to each participant for this pillar) *This pillar highlights the need to improve the safety of road networks and infrastructure for the benefit of all road users, including the pedestrians, bicyclists and motorcyclists. Activities include considering safety during the planning, design, construction and operation of roads; making sure that roads are regularly assessed for safety; and encouraging the relevant authorities to consider all forms of transport and types of safe infrastructure when they respond to the mobility needs of road users.*

Suggested questions and prompts

- What is your job title and what is the focus of your responsibilities for this position?
- How long have you been in this role?
  - Prompt: How these responsibilities are determined?
- From the description of Pillar 2, what is the status of activities for this pillar in Nepal?
- From your experience, what is going well?
  - *Prompt: Why do you think it is going well?*
- From you experience what has not yet happened or is not working well?
  - *Prompt: Why do you think it is not working well?*
- What challenges you are facing to achieve your desired objectives for road safety?
  - *Prompts*
    - *challenges to promote road safety ownership and accountability?*
    - *challenges promoting (addressing) the needs of all road users?*
    - *challenges relating to designing, building or maintaining roads?*
- What information or evidence do you think would help you achieve these objectives?
- Can you think of any gaps in the research available to you, related to these activities?
- Would you like to add anything which we have not covered during this conversation?

At the end of the interview

- Explain that this is the end of the interview.
- Thank the participant for their time.
- Explain that the information they have given will be used to create a list of possible research ideas to improve road safety in Nepal.
- Explain that they will be invited to the next stage of the study where they will hear all of the research questions we have identified and they will be invited to tell us which ones they think are the most important.
- Ask if they have any questions before you go.

Pillar 3. Safer vehicles

(To be read to each participant for this pillar) *Poor vehicle standards contribute to a significant number of crashes and casualties. This pillar encourages use of best practice vehicle safety standards and technology to promote safety. Activities may include implementing new car assessment programmes (such as NCAP safety ratings) and vehicle safety checks on existing vehicles to ensure they are equipped with minimum safety features, such as seat-belts to minimise the impact of crashes to occupants, and working lights and brakes.*

Suggested questions and prompts

- What is your job title and what is the focus of your responsibilities for this position?

For peer review only - http://bmjopen.bmj.com/site/about/guidelines.xhtml

- How long have you been in this role?
  - Prompt: How these responsibilities are determined?
- From the description of Pillar 3, what is the status of activities for this pillar in Nepal?
- From your experience, what is going well?
  - *Prompt: Why do you think it is going well?*
- From you experience what has not happened or is not working well?
  - *Prompts*
    - *Why do you think it is not working well?*
    - *What are your views on the New Car Assessment Programme (NCAP)?*
    - *do we have good vehicle-related laws that could promote the import of safer vehicles or the maintenance of existing vehicles?*
- What challenges you are facing to achieve desired objectives for road safety?
    - *challenges to harmonise international motor vehicle safety regulations with national laws?*
    - *research about safety technologies designed to reduce risk to vulnerable road users.*
- What information or evidence do you think would help you achieve these objectives?
- Can you think of any gaps in the research available to you, related to these activities?
- Would you like to add something else which we have not covered during this conversation?

At the end of the interview

- Explain that this is the end of the interview.
- Thank the participant for their time.
- Explain that the information they have given will be used to create a list of possible research ideas to improve road safety in Nepal.
- Explain that they will be invited to the next stage of the study where they will hear all of the research questions we have identified and they will be invited to tell us which ones they think are the most important.
- Ask if they have any questions before you go.

Pillar 4. Safer road users

(To be read to each participant for this pillar) *Pillar 4 focuses on developing comprehensive programmes to improve the behaviour of all road users. Activities include the adoption of model road safety legislation and sustained or increased enforcement or road safety laws and standards. These efforts are combined with public awareness and education to increase uptake of behaviours that keep people safe (e.g. seat-belt and helmet wearing) and to reduce behaviours that cause harm (e.g. speeding, taking alcohol or drugs when driving) and other risks. It also calls for activities to reduce work-related road traffic injuries and promoted the establishment of graduated driver licensing programmes for novice drivers.*

<u>Suggested questions and prompts</u>

- What is your job title and what is the focus of your responsibilities for this position?
- How long have you been in this role?
  - Prompt: How these responsibilities are determined?
- From the description of Pillar 4, what is the status of activities for this pillar in Nepal?
- From your experience, what is going well?

For peer review only - http://bmjopen.bmj.com/site/about/guidelines.xhtml

- *Prompt: Why do you think it is going well?*
- From you experience what has not happened or is not working well?
  - *Prompt: Why do you think it is not working well?*
- What challenges you are facing to achieve desired objectives for road safety?
  - *Prompts*
    - *what is the status of law enforcement?*
    - *what could be done to strengthen road safety law enforcement?*
    - *How good is the uptake of safe driver / passenger behaviours (e.g. seatbelt / helmet use)?*
    - *What role do driving licences play in road safety?*
    - *Are there any gaps in what we know about road user behaviours and how to change them?*
    - *what about gaps in legislation or how it is enforced relating to road users behaviour?*
- What new information or evidence do you think would help you to improve the uptake of safe road user behaviours?
- Can you think of any gaps in the research available to you, related to these activities in Pillar 4?
- Would you like to add something else which we have not covered during this conversation?

At the end of the interview

- Explain that this is the end of the interview.
- Thank the participant for their time.
- Explain that the information they have given will be used to create a list of possible research ideas to improve road safety in Nepal.
- Explain that they will be invited to the next stage of the study where they will hear all of the research questions we have identified and they will be invited to tell us which ones they think are the most important.
- Ask if they have any questions before you go.

Pillar 5. Post-crash response

(To be read to each participant for this pillar) *Pillar 5 addresses the need to improve the response to post-crash emergencies and the ability of health and other systems to provide appropriate emergency treatment and long-term rehabilitation for crash victims. The development and improvement of pre-hospital care systems, hospital trauma care systems, and rehabilitation along with long-term medical support to victims and a single emergency response number, are the main elements of post-impact care.*

Suggested questions and prompts

- What is your job title and what is the focus of your responsibilities for this position?
- How long have you been in this role?
  - Prompt: How these responsibilities are determined?
- From the description of Pillar 5, what is the status of activities for this pillar in Nepal?
- From your experience, what is going well?
  - *Prompt: Why do you think it is going well?*
- From you experience what is left behind or not working well?
  - *Prompt: Why do you think it is not working well?*

For peer review only - http://bmjopen.bmj.com/site/about/guidelines.xhtml

- What challenges you are facing to achieve desired objectives for road safety?
  - *Prompts*
    - *why there is no single nationwide telephone number for emergency services i.e. ambulances?*
    - *How do you see the performance of hospital trauma services in Nepal?*
- What new information or evidence do you think would help improve the provision of good post-crash response and care in Nepal?
- Can you think of any gaps in the research or information available to you in regard to the recommendations in Pillar 5?
- Would you like to add something else which we have not covered during this conversation?

At the end of the interview

- Explain that this is the end of the interview.
- Thank the participant for their time.
- Explain that the information they have given will be used to create a list of possible research ideas to improve road safety in Nepal.
- Explain that they will be invited to the next stage of the study where they will hear all of the research questions we have identified and they will be invited to tell us which ones they think are the most important.
- Ask if they have any questions before you go.

Below is the Nepali translation of the Guide.

For peer review only

For peer review only - http://bmjopen.bmj.com/site/about/guidelines.xhtml

UWE Bristol University of the West of England

नेपाल इन्जरी अनुसन्धान केन्द्र NIRC Nepal Injury Research Centre

अन्तरवार्ता निर्देशिका

नेपालमा सडक सुरक्षाका लागि अनुसन्धान प्राथमिकताहरू पहिचान गर्ने अध्ययन

स्तम्भ १. सडक सुरक्षा व्यवस्थापन

(To be read to each participant for this pillar)

सडक सुरक्षाको यो स्तम्भ देशमा सडक सुरक्षाको लागि बहु-संस्थागत क्षमता सुदृढीकरणमा केन्द्रित छ। यस स्तम्भमा देशमा सडक सुरक्षाका लागि संयुक्त राष्ट्रसंघीय महासन्धीहरूलाई अभ्यास गर्ने, सडक सुरक्षा गतिविधिहरूको नेतृत्व गर्ने बहु-पक्षीय राष्ट्रिय निकायको स्थापना गर्ने, राष्ट्रिय सडक सुरक्षा रणनीति विकास गर्ने र सम्बन्धित गतिविधिहरूको कार्यान्वयनको लागि यथार्थवादी र दीर्घकालीन लक्ष्यहरूको निर्धारण गर्नाका साथै तिनको कार्यान्वयनका लागि पर्याप्त रकमको सुनिश्चतता गर्ने कृयाकलापहरू पर्दछन्। यस स्तम्भले उपर्युक्त कृयाकलापहरूको प्रभावकारी रूपमा अनुगमन र मूल्याङ्कन गर्नको लागि तथ्यांक प्रणालीको विकासको लागि पनि आह्वान गर्दछ।

For peer review only

<u>Questions and prompts</u>

- तपाई कुन पदमा कार्यरत हुनुहुन्छ र तपाईंको मुख्य जिम्मेवारीहरु के के हुन्?
- तपाई यस पदमा कति समयदेखि हुनुहुन्छ ?
  - प्रम्प्टः यस पदका लागि जिम्मेवारी कसरी निर्धारण भएका हुन्छन् ?
- स्तम्भ १ को वर्णनबाट तपाईं को विचारमा नेपालमा यस स्तम्भ अन्तर्गत गरिएका कृयाकलापहरूको अवस्था कस्तो छ ?
- तपाईको अनुभवबाट यस स्तम्भ अन्तरगतका कुन कुन कृयाकलापहरू राम्रोसँग अघि बढिरहेका छन् ?
  - प्रम्प्ट: तपाईंको विचारमा किन यो राम्रो हुँदैछ **?**
- तपाईको अनुभवमा यस स्तम्भ अन्तर्गतका कुन कामहरू राम्ररी भइरहेका छैन वा राम्रोसँग गर्न सकिएको छैन ?
  - प्रम्प्ट: तपाईंको विचारमा किन ती कृयाकलापहरू राम्रोसँग गर्न सकिएको छैन ?
- सडक सुरक्षाको लागि अपेक्षित उद्देश्यहरू प्राप्त गर्न तपाइँले के कस्ता चुनौतीहरू सामना गर्नु परेको छ ?
    - सडक सुरक्षाको नेतृत्व गर्ने संस्था वा निकायको स्थापनाबारे चुनौतिहरू?
    - राष्ट्रिय रणनीति बनाउने सम्बन्धी चुनौतिहरू?
    - तथ्यांक उत्पादन (Data generation) सम्बन्धी चुनौतिहरू?
    - अनुगमन र मूल्यांकन सम्बन्धी चुनौतिहरू?
- कस्ता जानकारी वा तथ्यहरू (Evidence) उपलब्ध भइदिएको भए तपाईंलाई यी उद्देश्यहरू प्राप्त गर्न मद्दत पुग्थ्यो जस्तो लाग्छ ?
- के तपाइँ स्तम्भ १ का यी गतिविधिहरूसँग सम्बन्धित अनुसन्धानमा कुनै कमी वा अन्तरहरू (Research Gaps) बारे सोचेर केही बताउन सक्नुहुन्छ ?
- हाम्रो यस कुराकानीको क्रममा केहि कुरा छुटेको पाउनु भए कृपया थप्नुहोस?

For peer review only - http://bmjopen.bmj.com/site/about/guidelines.xhtml

At the end of the interview

- हामी अन्तर्वार्ताको अन्त्यमा पुग्यौं
- तपाईंको सहभागिताको लागि धन्यवाद
- तपाईंले दिनुभएको जानकारीले नेपालमा सडक सुरक्षाको स्थितिमा सुधार ल्याउन सम्भावित अनुसन्धानको लागि सुची बनाउन मद्दत गर्नेछ।
- हामी तपाईलाई यस अध्ययनको दोस्रो चरणको लागि पनि सम्पर्क गर्नेछौं जसमा हामी अन्तरवार्ताहरूबाट पहिचान गरेका सबै अनुसन्धान प्रश्नहरु प्रस्तुत गर्नेछौं र त्यसमध्ये कुन सबैभन्दा महत्वपुर्ण छ भनेर तपाईंको प्रतिक्रिया लिनेछौं ।
- अन्तिममा तपाईंको केहि प्रश्न छ कि?

स्तम्भ २. सुरक्षित सडक र गतिशिलता

(To be read to each participant for this pillar)

यो स्तम्भले सबै पैदलयात्री, साइकल चालक, मोटरसाइकल चालक लगायत सबै सडक प्रयोगकर्ताको फाइदाको लागि आवश्यक सुरक्षित सडक संजाल एवं पूर्वाधार सुधारलाई जोड दिन्छ । यस अन्तरगतका कृयाकलापहरूमा सडकको योजना, निर्माण तथा संचालनका चरणहरूमा नैं सडक सुरक्षालाई ध्यानमा राख्नु पर्ने; सडकको सुरक्षाको नियमित मुल्यांकन गरिनेछ भनेर सुनिस्चित गर्ने; र सम्बद्ध अधिकारीलाई सडक प्रयोगकर्ताको गतिशिलता (mobility) आवश्यकतालाई सम्बोधन गर्दा सबै किसिम र प्रकारका यातायात तथा पूर्वाधारलाई सुरक्षित पार्न प्रोत्साहन गर्ने जस्ता क्रियाकलापहरु पर्छन् ।

<u>**Suggested questions and prompts**</u>

- तपाई कुन पदमा कार्यरत हुनुहुन्छ र तपाईंको मुख्य जिम्मेवारीहरु के के हुन्?
- तपाई यस पदमा कति समयदेखि हुनुहुन्छ ?
    - प्रम्प्टः यस पदका लागि जिम्मेवारी कसरी निर्धारण भएका हुन्छन् ?
- स्तम्भ २ को वर्णनबाट तपाईं को विचारमा नेपालमा यस स्तम्भ अन्तरगत गरिएका कृयाकलापहरूको अवस्था कस्तो छ ?
- तपाईको अनुभवबाट यस स्तम्भ अन्तरगतका कुन कुन कृयाकलापहरू राम्रोसँग अघि बढिरहेका छन् ?
    - प्रम्प्ट: तपाईंको विचारमा किन यो राम्रो हुँदैछ ?
- तपाईको अनुभवमा यस स्तम्भ अन्तरगतका कुन कामहरू राम्ररी भइरहेका छैन वा राम्रोसँग गर्न सकिएको छैन ?
    - प्रम्प्ट: तपाईंको विचारमा किन ती कृयाकलापहरू राम्रोसँग गर्न सकिएको छैन ?
- सडक सुरक्षाको लागि अपेक्षित उद्देश्यहरू प्राप्त गर्न तपाइँले के कस्ता चुनौतीहरू सामना गर्दै हुनुहुन्छ ?
        - सडक सुरक्षाको स्वामित्व र उत्तरदायित्व प्रवर्धनसम्बन्धी चुनौती?
        - सबै सडक प्रयोगकर्ताहरूको आवश्यकतालाई सम्बोधन गर्नेबारे चुनौतीहरू?
        - सडक डिजाइन, निर्माण वा मर्मतसँग सम्बन्धी चुनौतिहरू?
- कस्ता जानकारी वा तथ्यहरू (Evidence) उपलब्ध भइदिएको भए तपाईंलाई यी उद्देश्यहरू प्राप्त गर्न मद्दत पुग्थ्यो जस्तो लाग्छ ?
- के तपाइँ स्तम्भ २ का य्ी गतिविधिहरुसंग सम्बन्धित अनुसन्धानमा कुनै कमी वा अन्तरहरू (Gaps) बारे सोचेर केही बताउन सक्नुहुन्छ ?

For peer review only - http://bmjopen.bmj.com/site/about/guidelines.xhtml

- हाम्रो यस कुराकानीको क्रममा केहि कुरा छुटेको पाउनु भए कृपया थप्नुहोस?

At the end of the interview
- हामी अन्तर्वार्ताको अन्त्यमा पुग्यौं
- तपाईंको सहभागिताको लागि धन्यवाद
- तपाईंले दिनुभएको जानकारीले नेपालमा सडक सुरक्षाको स्थितिमा सुधार ल्याउन सम्भावित अनुसन्धानको लागि सुची बनाउन मद्दत गर्नेछ।
- हामी तपाईलाई यस अध्ययनको दोस्रो चरणको लागि पनि सम्पर्क गर्नेछौं जसमा हामी अन्तरवार्ताहरूबाट पहिचान गरेका सबै अनुसन्धान प्रश्नहरु प्रस्तुत गर्नेछौं र त्यसमध्ये कुन सबैभन्दा महत्वपुर्ण छ भनेर तपाईंको प्रतिक्रिया लिनेछौं ।
- अन्तिममा तपाईंको केहि प्रश्न छ कि?

स्तम्भ ३ सुरक्षित वाहनहरू

(To be read to each participant for this pillar)
कमसल मापदण्ड भएका वाहनहरूले उल्लेखनीय संख्यामा सडक दुर्घटना र हताहती गराइरहेका हुन्छन्। यस स्तम्भले सुरक्षा प्रबर्धन गर्न उत्तम अभ्यास, वाहन सुरक्षाका मानकहरू र प्रविधिको प्रयोग गर्न प्रोत्साहन गर्दछ। यस अन्तरगतका गतिविधिहरूमा नयाँ कारको सुरक्षा मुल्यांकन कार्यक्रम (NCAP सुरक्षा रेटिंगहरू) को कार्यान्वयन गर्ने, विद्यमान सवारी साधनहरूमा न्यूनतम सुरक्षा सुविधाहरू भएको सुनिश्चत गर्न वाहन सुरक्षा जाँचहरू समावेश गर्ने, जस्तै दुर्घटनामा परेका व्यक्तिमा दुर्घटनाको प्रभाव कम गर्न सीट बेल्ट जोडिएको र बत्ती एवम् ब्रेक ठीक अवस्थामा छ भन्ने सुनिश्चत गर्दछ ।

<u>Suggested questions and prompts</u>
- तपाई कुन पदमा कार्यरत हुनुहुन्छ र तपाईंको मुख्य जिम्मेवारीहरु के के हुन्?
- तपाई यस पदमा कति समयदेखि हुनुहुन्छ ?
  - प्रम्टः यस पदका लागि जिम्मेवारी कसरी निर्धारण भएका हुन्छन् ?
- स्तम्भ ३ को वर्णनबाट तपाईं को विचारमा नेपालमा यस स्तम्भ अन्तरगत गरिएका कृयाकलापहरूको अवस्था कस्तो छ ?
- तपाईको अनुभवबाट यस स्तम्भ अन्तरगतका कुन कुन कृयाकलापहरू राम्रोसँग अघि बढिरहेका छन् ?
  - <u>प्रम्ट</u>: तपाईंको विचारमा किन यो राम्रो हुँदैछ **?**
- तपाईको अनुभवमा यस स्तम्भ अन्तरगतका कुन कामहरू राम्ररी भइरहेका छैन वा राम्रोसँग गर्न सकिएको छैन ?
    - तपाईंको विचारमा किन ती कृयाकलापहरू राम्रोसँग गर्न सकिएको छैन ?
    - NCAP सुरक्षा रेटिंगहरू (अथवा नयाँ कारको सुरक्षा मुल्यांकन कार्यक्रम) सम्बन्धमा तपाईंको के विचार छ?
    - के हामीसँग सवारी साधन सम्बन्धी राम्रा कानूनहरू छन् जसले बढी सुरक्षित गाडीहरूको आयात बढाउने वा विद्यमान सवारी साधनहरूमा सुधार वा मर्मत गरी तिनलाइ सुरक्षित पार्न मद्दत गर्दछन्?
- सडक सुरक्षाको लागि अपेक्षित उद्देश्यहरू प्राप्त गर्न तपाइँले के कस्ता चुनौतीहरू सामना गर्दै हुनुहुन्छ ?
    - राष्ट्रिय कानूनहरूलाई अन्तर्राष्ट्रिय मोटर वाहन सुरक्षा नियमहरुसँग सामंजस्यता गर्दाका चुनौतिहरु?

For peer review only - http://bmjopen.bmj.com/site/about/guidelines.xhtml

  - जोखिममा रहेका सडक प्रयोगकर्ताहरूका जोखिम कम गर्न डिजाइन गरिएको सुरक्षा प्रविधिहरूका बारे अनुसन्धानसम्बन्धी चुनौतिहरुरू
- कस्ता जानकारी वा तथ्यहरू (Evidence) उपलब्ध भइदिएको भए तपाईंलाई यी उद्देश्यहरू प्राप्त गर्न मद्दत पुग्थ्यो जस्तो लाग्छ ?
- के तपाइँ स्तम्भ ३ का यी गतिविधिहरुसंग सम्बन्धित अनुसन्धानमा कुनै कमी वा अन्तरहरू (Gaps) बारे सोचेर केही बताउन सक्नुहुन्छ ?
- हाम्रो यस कुराकानीको क्रममा केहि कुरा छुटेको पाउनु भए कृपया थप्नुहोस?

At the end of the interview
- हामी अन्तर्वार्ताको अन्त्यमा पुग्यौं
- तपाईंको सहभागिताको लागि धन्यवाद
- तपाईंले दिनुभएको जानकारीले नेपालमा सडक सुरक्षाको स्थितिमा सुधार ल्याउन सम्भावित अनुसन्धानको लागि सुची बनाउन मद्दत गर्नेछ।
- हामी तपाईलाई यस अध्ययनको दोस्रो चरणको लागि पनि सम्पर्क गर्नेछाैं जसमा हामी अन्तरवार्ताहरूबाट पहिचान गरेका सबै अनुसन्धान प्रश्नहरु प्रस्तुत गर्नेछाैं र त्यसमध्ये कुन सबैभन्दा महत्वपुर्ण छ भनेर तपाईंको प्रतिक्रिया लिनेछौं ।
- अन्तिममा तपाईंको केहि प्रश्न छ कि?

स्तम्भ ४ सुरक्षित सडक प्रयोगकर्ताहरू

(To be read to each participant for this pillar)
स्तम्भ ४ सबै सडक प्रयोगकर्ताहरूको व्यवहार सुधार गर्न व्यापक कार्यक्रमहरू विकासमा केन्द्रित छ। यस अन्तरगत उदाहरणीय सडक सुरक्षा कानून र मापदण्ड अपनाउने र तिनको निरन्तर वा बढ्दो क्रममा पालनामा गराउने गतिविधिहरू समावेश छन। यस्ता प्रयासहरूमा जनचेतना र मानिसहरुलाई सुरक्षित राख्ने उपायहरू जस्तैः सीट बेल्ट र हेलमेट लगाउने बानी प्रवर्द्धन गर्न र तीव्र गतिमा वाहन चलाउने, रक्सी वा मादकपदार्थ सेवन गरी गाडी चलाउने र यस्तै अन्य जोखिमपूर्ण व्यवहारमा कमी ल्याउन व्यवहारिक शिक्षा दिने कृयाकलापहरू पनि समावेश छन्। यस स्तम्भले कामसँग सम्बन्धित (पेशागत) सडक दुर्घटनाहरू कम गर्ने गतिविधिहरूको लागि आह्वान गर्दछ र भरखरै चालक अनुमतिपत्र (लाइसेन्स) लिएका नयाँ चालकहरूको लागि उनीहरूको सवारी चालक अनुमतिपत्र (लाइसेन्स) लाई क्रमैसँग स्तरोन्नति गर्ने (graduated driver licensing) कार्यक्रमहरूलाई बढावा दिन अह्वान गरेको छ।

<u>Suggested questions and prompts</u>
- तपाई कुन पदमा कार्यरत हुनुहुन्छ र तपाईंको मुख्य जिम्मेवारीहरु के के हुन्?
- तपाई यस पदमा कति समयदेखि हुनुहुन्छ ?
  - प्रम्ट: यस पदका लागि जिम्मेवारी कसरी निर्धारण भएका हुन्छन् ?
- स्तम्भ ४ को वर्णनबाट तपाईं को विचारमा नेपालमा यस स्तम्भ अन्तरगत गरिएका कृयाकलापहरूको अवस्था कस्तो छ ?
- तपाईको अनुभवबाट यस स्तम्भ अन्तरगतका कुन कुन कृयाकलापहरू राम्रोसँग अघि बढिरहेका छन् ?
  - <u>प्रम्ट</u>: तपाईंको विचारमा किन यो राम्रो हुँदैछ **?**

For peer review only - http://bmjopen.bmj.com/site/about/guidelines.xhtml

- तपाईको अनुभवमा यस स्तम्भ अन्तरगतका कुन कामहरू राम्ररी भइरहेका छैन वा राम्रोसँग गर्न सकिएको छैन ?
    - तपाईंको विचारमा किन ती कृयाकलापहरू राम्रोसँग गर्न सकिएको छैन ?
- सडक सुरक्षाको लागि अपेक्षित उद्देश्यहरू प्राप्त गर्न तपाईँले के कस्ता चुनौतीहरू सामना गर्दै हुनुहुन्छ ?
        - कानूनको पालना र कार्यान्वयनको स्थिति कस्तो छ?
        - सडक सुरक्षा कानूनको पालनालाई सुदृढ पार्न के गर्न सकिन्छ?
        - सुरक्षित चालक / यात्री व्यवहारको अवलम्बन गर्ने चलन कस्तो छ (उदाहरणको लागि सीटबेल्ट / हेलमेट प्रयोग, मा.प.से.)
        - सडक सुरक्षाको लागि चालक अनुमतिपत्र (लाइसेन्स) के भूमिका खेल्छ?
        - सडक प्रयोगकर्ताका व्यवहारसँग सम्बन्धित कुनैं कमीकमजोरीहरू हामीलाई थाहा छन् ? तिनलाई कसरी परिवर्तन गर्न सकिन्छ ?
        - सडक सुरक्षा सम्बन्धी कानूनी प्रावधानमा के कस्ता कमी कमजोरी छन् ? तिनको पालनालाई कसरी सडक प्रयोगकर्ताहरूको व्यवहारसँग जोडिएकोछ ?
- कुन नयाँ जानकारी वा तथ्य उपलब्ध भइदिए सडक प्रयोगकर्ताहरूको सुरक्षित व्यवहार अवलम्बनलाई सुधार गर्न मद्दत पुग्नेछ?
- के तपाइँ स्तम्भ ४ का यी गतिविधिहरुसंग सम्बन्धित अनुसन्धानमा कुनै कमी वा अन्तरहरू (Gaps) बारे सोचेर केही बताउन सक्नुहुन्छ ?
- हाम्रो यस कुराकानीको क्रममा केहि कुरा छुटेको पाउनु भए कृपया थप्नुहोस?

At the end of the interview
- हामी अन्तर्वार्ताको अन्त्यमा पुग्यौं
- तपाईंको सहभागिताको लागि धन्यवाद
- तपाईंले दिनुभएको जानकारीले नेपालमा सडक सुरक्षाको स्थितिमा सुधार ल्याउन सम्भावित अनुसन्धानको लागि सुची बनाउन मद्दत गर्नेछ।
- हामी तपाईलाई यस अध्ययनको दोस्रो चरणको लागि पनि सम्पर्क गर्नेछौं जसमा हामी अन्तरवार्ताहरूबाट पहिचान गरेका सबै अनुसन्धान प्रश्नहरु प्रस्तुत गर्नेछौं र त्यसमध्ये कुन सबैभन्दा महत्वपुर्ण छ भनेर तपाईंको प्रतिक्रिया लिनेछौं ।
- अन्तिममा तपाईंको केहि प्रश्न छ कि?

स्तम्भ ५. दुर्घटनापश्चातको स्याहार

(To be read to each participant for this pillar)
स्तम्भ ५ ले दुर्घटना पछिको इमरजेन्सीकोलागि प्रतिक्रियामा सुधारको साथै दुर्घटनाका घाइतेलाई उपयुक्त इमरजेन्सी उपचार सेवा एवम् दीर्घकालीन पुनर्स्थापना सेवा दिने स्वास्थ्य र अन्य प्रणालीको क्षमताको सुधारको आवश्यकतालाई सम्बोधन गर्दछ। घाइतेलाई अस्पताल लैजानु अघि गर्नु पर्ने स्याहार प्रणाली, अस्पताल ट्रामा स्याहार प्रणाली, पीडितलाई पुनर्स्थापनाका साथै दीर्घकालीन चिकित्सा सहायता प्रणालीको विकास एवम् सुधार र इमरजेन्सी अवस्थामा सहयोग लिन प्रयोग गरिने एउटै नम्बरको बिकास र सुधारका कृयाकलापहरू दुर्घटना पश्चातको स्याहारका मुख्य बुँदाहरू हुन् ।

Suggested questions and prompts
- तपाई कुन पदमा कार्यरत हुनुहुन्छ र तपाईंको मुख्य जिम्मेवारीहरु के के हुन्?
- तपाई यस पदमा कति समयदेखि हुनुहुन्छ ?

For peer review only - http://bmjopen.bmj.com/site/about/guidelines.xhtml

  - प्रम्ट: यस पदका लागि जिम्मेवारी कसरी निर्धारण भएका हुन्छन् ?
- स्तम्भ १ को वर्णनबाट तपाईं को विचारमा नेपालमा यस स्तम्भ अन्तरगत गरिएका कृयाकलापहरूको अवस्था कस्तो छ ?
- तपाईको अनुभवबाट यस स्तम्भ अन्तरगतका कुन कुन कृयाकलापहरू राम्रोसँग अघि बढिरहेका छन् ?
  - प्रम्ट: तपाईंको विचारमा किन यो राम्रो हुँदैछ ?
- तपाईको अनुभवमा यस स्तम्भ अन्तरगतका कुन कामहरू राम्ररी भइरहेका छैन वा राम्रोसँग गर्न सकिएको छैन ?
  - तपाईंको विचारमा किन ती कृयाकलापहरू राम्रोसँग गर्न सकिएको छैन ?
- सडक सुरक्षाको लागि अपेक्षित उद्देश्यहरू प्राप्त गर्न तपाइँले के कस्ता चुनौतीहरू सामना गर्नु परेको छ ?
    - किन देशभरी नै आपत्कालिन सेवा जस्तै एम्बुलेन्स सेवाको लागी एउटै टेलिफोन नम्बर छैन ?
    - तपाईं नेपालका अस्पतालको ट्रमा सेवाहरूलाई कसरी हेर्नुहुन्छ ?
- कुन नयाँ जानकारी वा तथ्य उपलव्ध भइदिए सडक प्रयोगकर्ताहरूको सुरक्षित व्यवहार अवलम्बनलाई सुधार गर्न मद्दत पुग्नेछ?
- के तपाइँ स्तम्भ ५ का यी गतिविधिहरुसंग सम्बन्धित अनुसन्धानमा कुनै कमी वा अन्तरहरू (Gaps) बारे सोचेर केही बताउन सक्नुहुन्छ ?
- हाम्रो यस कुराकानीको क्रममा केहि कुरा छुटेको पाउनु भए कृपया थप्नुहोस?

At the end of the interview

- हामी अन्तर्वार्ताको अन्त्यमा पुग्यौं
- तपाईंको सहभागिताको लागि धन्यवाद
- तपाईंले दिनुभएको जानकारीले नेपालमा सडक सुरक्षाको स्थितिमा सुधार ल्याउन सम्भावित अनुसन्धानको लागि सुची बनाउन मद्दत गर्नेछ।
- हामी तपाईलाई यस अध्ययनको दोस्रो चरणको लागि पनि सम्पर्क गर्नेछौं जसमा हामी अन्तरवार्ताहरूबाट पहिचान गरेका सबै अनुसन्धान प्रश्नहरु प्रस्तुत गर्नेछौं र त्यसमध्ये कुन सबैभन्दा महत्वपुर्ण छ भनेर तपाईंको प्रतिक्रिया लिनेछौं ।
- अन्तिममा तपाईंको केहि प्रश्न छ कि?

For peer review only - http://bmjopen.bmj.com/site/about/guidelines.xhtml

CREDES checklist adapted from Saskia et al 2017. *Guidance on Conducting and REporting DElphi Studies (CREDES) in palliative care: Recommendations based on a methodological systematic review*. Palliative Medicine. available from https://journals.sagepub.com/doi/10.1177/0269216317690685

Box 3. Recommendations for the Conducting and REporting of DElphi Studies (CREDES).

Rationale for the choice of the Delphi technique

1. *Justification.* The choice of the Delphi technique as a method of systematically collating expert consultation and building consensus needs to be well justified. When selecting the method to answer a particular research question, it is important to keep in mind its constructivist nature

Planning and design

2. *Planning and process.* The Delphi technique is a flexible method and can be adjusted to the respective research aims and purposes. Any modifications should be justified by a rationale and be applied systematically and rigorously
3. *Definition of consensus.* Unless not reasonable due to the explorative nature of the study, an a priori criterion for consensus should be defined. This includes a clear and transparent guide for action on (a) how to proceed with certain items or topics in the next survey round, (b) the required threshold to terminate the Delphi process and (c) procedures to be followed when consensus is (not) reached after one or more iterations

Study conduct

4. *Informational input.* All material provided to the expert panel at the outset of the project and throughout the Delphi process should be carefully reviewed and piloted in advance in order to examine the effect on experts' judgements and to prevent bias
5. *Prevention of bias.* Researchers need to take measures to avoid directly or indirectly influencing the experts' judgements. If one or more members of the research team have a conflict of interest, entrusting an independent researcher with the main coordination of the Delphi study is advisable
6. *Interpretation and processing of results.* Consensus does not necessarily imply the 'correct' answer or judgement; (non)consensus and stable disagreement provide informative insights and highlight differences in perspectives concerning the topic in question
7. *External validation.* It is recommended to have the final draft of the resulting guidance on best practice in palliative care reviewed and approved by an external board or authority before publication and dissemination

Reporting

8. *Purpose and rationale.* The purpose of the study should be clearly defined and demonstrate the appropriateness of the use of the Delphi technique as a method to achieve the research aim. A rationale for the choice of the Delphi technique as the most suitable method needs to be provided
9. *Expert panel.* Criteria for the selection of experts and transparent information on recruitment of the expert panel, socio-demographic details including information on expertise regarding the topic in question, (non)response and response rates over the ongoing iterations should be reported
10. *Description of the methods.* The methods employed need to be comprehensible; this includes information on preparatory steps (How was available evidence on the topic in question synthesised?), piloting of material and survey instruments, design of the survey instrument(s), the number and design of survey rounds, methods of data analysis, processing and synthesis of experts' responses to inform the subsequent survey round and methodological decisions taken by the research team throughout the process
11. *Procedure.* Flow chart to illustrate the stages of the Delphi process, including a preparatory phase, the actual 'Delphi rounds', interim steps of data processing and analysis, and concluding steps
12. *Definition and attainment of consensus.* It needs to be comprehensible to the reader how consensus was achieved throughout the process, including strategies to deal with non-consensus
13. *Results.* Reporting of results for each round separately is highly advisable in order to make the evolving of consensus over the rounds transparent. This includes figures showing the average group response, changes between rounds, as well as any modifications of the survey instrument such as deletion, addition or modification of survey items based on previous rounds
14. *Discussion of limitations.* Reporting should include a critical reflection of potential limitations and their impact of the resulting guidance
15. *Adequacy of conclusions.* The conclusions should adequately reflect the outcomes of the Delphi study with a view to the scope and applicability of the resulting practice guidance
16. *Publication and dissemination.* The resulting guidance on good practice in palliative care should be clearly identifiable from the publication, including recommendations for transfer into practice and implementation. If the publication does not allow for a detailed presentation of either the resulting practice guidance or the methodological features of the applied Delphi technique, or both, reference to a more detailed presentation elsewhere should be made (e.g. availability of the full guideline from the authors or online; publication of a separate paper reporting on methodological details and particularities of the process (e.g. persistent disagreement and controversy on certain issues)). A dissemination plan should include endorsement of the guidance by professional associations and health care authorities to facilitate implementation

Item#	Description	Section/Page # reported in the manuscript
Rationale for the choice of the Delphi technique		
1	Justification.	Methods, first paragraph, page 4
Planning and design		
2	Planning and process	METHODS, pages 4-6

3	Definition of Consensus	Data collection and analysis, page 5, paragraph 2
Study conduct		
4	Informational input	Recruitment of participants, page 4
5	Prevention of bias	Strengths and limitations, page 14-15
6	Interpretation and processing results	Data collection, paragraphs 2-4, pages 5-6
7	External validations	Overarching consensus workshop, page 6.
Reporting		
8	Purpose and Rationale	INTRODUCTION, page 3
9	Expert panel	Description of participants, Results, pages 6-7 and Table 1
10	Description of the methods	METHODS, pages 4-6
11	Procedure	METHODS, pages 4-6 Flow chart, Figure 1, page 19
12	Definition and attainment of consensus	Paragraphs 2 and 3 of Data collection and analysis, pages 5-6 + overall consensus workshop paragraph, page 6
13	Results	Results pages 6-11, Table 3 (page 9-11) and Table 4 (page 11)
14	Discussion of limitations	Strengths and limitations, page 14
15	Adequacy of conclusions	CONCLUSIONS, page 15
16	Publication and dissemination	Not applicable as this is not a Delphi study supporting guidelines for clinical practice.

For peer review only

BMJ Open

Identifying research priorities for road safety in Nepal: A Delphi study

Journal:	*BMJ Open*
Manuscript ID	bmjopen-2021-059312.R1
Article Type:	Original research
Date Submitted by the Author:	24-Feb-2022
Complete List of Authors:	Pant, Puspa Raj; University of the West of England, Faculty of Health and Applied Sciences<br>Rana, Pranita; Kathmandu Medical College, Nepal Injury Research Centre<br>Pradhan, Kriti; Kathmandu Medical College, Nepal Injury Research Centre<br>Joshi, Sunil Kumar; Kathmandu Medical College and Teaching Hospital, Community Medicine<br>Mytton, Julie; University of the West of England, Faculty of Health and Applied Sciences
**Primary Subject Heading**:	Health policy
Secondary Subject Heading:	Public health
Keywords:	Health policy < HEALTH SERVICES ADMINISTRATION & MANAGEMENT, PUBLIC HEALTH, ACCIDENT & EMERGENCY MEDICINE

SCHOLARONE™ Manuscripts

I, the Submitting Author has the right to grant and does grant on behalf of all authors of the Work (as defined in the below author licence), an exclusive licence and/or a non-exclusive licence for contributions from authors who are: i) UK Crown employees; ii) where BMJ has agreed a CC-BY licence shall apply, and/or iii) in accordance with the terms applicable for US Federal Government officers or employees acting as part of their official duties; on a worldwide, perpetual, irrevocable, royalty-free basis to BMJ Publishing Group Ltd ("BMJ") its licensees and where the relevant Journal is co-owned by BMJ to the co-owners of the Journal, to publish the Work in this journal and any other BMJ products and to exploit all rights, as set out in our licence.

The Submitting Author accepts and understands that any supply made under these terms is made by BMJ to the Submitting Author unless you are acting as an employee on behalf of your employer or a postgraduate student of an affiliated institution which is paying any applicable article publishing charge ("APC") for Open Access articles. Where the Submitting Author wishes to make the Work available on an Open Access basis (and intends to pay the relevant APC), the terms of reuse of such Open Access shall be governed by a Creative Commons licence – details of these licences and which Creative Commons licence will apply to this Work are set out in our licence referred to above.

Other than as permitted in any relevant BMJ Author's Self Archiving Policies, I confirm this Work has not been accepted for publication elsewhere, is not being considered for publication elsewhere and does not duplicate material already published. I confirm all authors consent to publication of this Work and authorise the granting of this licence.

For peer review only

Identifying research priorities for road safety in Nepal: A Delphi study

Corresponding author: Dr Puspa Raj Pant[a*]

[a]Faculty of Health and Applied Sciences, University of the West of England, Bristol, BS16 1QY, United Kingdom, Email: pant.puspa@gmail.com ORCiD: https://orcid.org/0000-0002-8827-0018

Co-authors:

Ms Pranita Rana[b], [b]Nepal Injury Research Centre, Kathmandu Medical College Public Ltd, Kathmandu, Nepal.

Ms Kriti Pradhan[b], [b]Nepal Injury Research Centre, Kathmandu Medical College Public Ltd, Kathmandu, Nepal.

Prof Sunil Kumar Joshi[c], [c]Department of Community Medicine, Kathmandu Medical College Public Ltd, Kathmandu, Nepal. ORCiD: https://orcid.org/0000-0002-2704-5060

Prof Julie Mytton[a], [a]Faculty of Health and Applied Sciences, University of the West of England, Bristol, UK. ORCiD: https://orcid.org/0000-0002-0306-4750

Word count: 4,218(excluding title page, abstract, references, figures and tables.)

Abstract: (287 words)

Objective: To identify and prioritise the research needed to help Nepali agencies develop an improved road safety system.

Design: Delphi study

Setting: Nepal

Participants: Stakeholders from government institutions, academia, engineering, healthcare and civil society, were interviewed to identify knowledge gaps and research questions. Participants then completed two rounds of ranking and a workshop.

Results: 93 participants took part in a total of 95 interviews. Participants were grouped with others sharing expertise relating to each of the five World Health Organisation's 'pillars' of road safety: 1) Road Safety Management; 2) Safer Roads; 3) Safer Vehicles; 4) Safer Road Users and 5) effective post-crash response.Interviews yielded 1019 research suggestions across the five pillars. Two rounds of ranking within expert groups yielded consensus on the important questions for each pillar.. A

For peer review only - http://bmjopen.bmj.com/site/about/guidelines.xhtml

workshop involving all participants then led to the selection of 6 questions considered the most urgent: (1) How can implementing agencies be made more accountable? (2) How should different types of roads, and roads in different geographical locations, be designed to make them safer for all road users? (3) What vehicle fitness factors are leading to road traffic crashes? (4) How can the driver licensing system be improved to ensure safer drivers? (5) What factors lead to public vehicle crashes and how can they be addressed? (6) What factors affect emergency response services getting to the patient and then getting them to the right hospital in the best possible time?

Conclusions: The application of the Delphi approach is useful to enable participants representing a range of institutions and expertise to contribute to the identification of road safety research priorities. Outcomes from this study provide Nepali researchers with a greater understanding of the necessary focus for future road safety research.

For peer review only

Keywords:

Safer Road users, Road Traffic Injuries, Road Safety Pillars, Post-crash Response, Delphi.

Strengths and limitations

- Ninety-three Nepali experts (70% of 133 approached) participated; bring perspectives from road construction, vehicle management, transport management and post-crash response.
- Most participants had a remit for national road safety, however, 83/93 (89%) were from organisations based in Kathmandu valley, which may have risked a focus on urban and highway crashes.
- The research questions identified were ranked by the participants individually as well as discussed during group meetings to achieve consensus.
- The Delphi approach is at risk of high dropout of participants; we were able to retain a high proportion of participants through the study – 64/93 participants took part in Round 3 (69% retention).

INTRODUCTION

Globally road traffic injuries are increasing, with an estimated 1.35 million deaths and up to 50 million non-fatal injuries in 2016. [1] Despite having only 1% of the world's vehicles, low-income countries have 13% of fatal road traffic injuries. Road traffic injuries are the leading cause of death for children and young adults between 5-29 years globally, and are an important cause of disability

For peer review only - http://bmjopen.bmj.com/site/about/guidelines.xhtml

and poverty. RTIs have been estimated to generate losses of up to 6.5% of a low-income country's gross domestic product. [2]

The World Health Organization (WHO) World Report on Road Traffic Injury Prevention, [3] subsequent Road Safety Status Reports [1] and the WHO Save LIVES technical package of 22 evidence-based interventions [4] argue for a "safe systems approach" [5] to reduce road dangers and the numbers of people killed and seriously injured on the roads. This approach recognises the essential contribution of different sectors to create a system that keeps road users safe. The World Health Organization published the Global Plan of Action for Road Safety 2011-2020 [6] alongside the United Nations and this plan of action recommended five 'pillars'; road safety management (Pillar 1), safer roads and mobility (Pillar 2), safer vehicles (Pillar 3), safer road users (Pillar 4) and post-crash response (Pillar 5). Action across all five pillars can contribute to reduced road traffic injuries. Nepal has been a co-sponsor to these principles, but progress has been limited.

A huge road construction programme in Nepal has seen over 15,000 km of new blacktop, gravel and earthen roads built by federal, provincial and local governments in the last 5 years [7] and there are plans to have a total of 13,500 km blacktopped road by 2023/24. [8] Many new roads do not have proven safety features and are poorly maintained. The roads in the hills are considered to be dangerous because of landslides in addition to frequent road crashes due to poor engineering or poor safety infrastructure. [9] The Department of Transport Management in the Government of Nepal produces vehicle registration statistics that show more than half (53%) of the 3.22 million motorised vehicles in Nepal were registered between July 2013 and July 2018 and about 78% of total registered vehicles were motorcycles. [10]

Nepal lacks a funded road safety implementation plan, a national ambulance service or globally recognised vehicle standards. The national helmet wearing law is not enforced for motorcycle passengers and there is no legislation for passenger seatbelt use, child restraints or mobile phone use whilst driving. Data are limited and of poor quality; WHO estimates of road traffic fatalities in Nepal in 2016 (4,622) are more than double those recorded by the Traffic Police (2,006), and there are no routinely published estimates of deaths by road user category available. [1] Nepal's Health Management Information System recorded over 100,000 hospital visits for the treatment of orthopaedic problems secondary to road traffic events in the year 2017/18 indicating the significant burden of road traffic injuries on health systems. [11] Road traffic crashes and injuries in Nepal are rising despite existing legislation. [12 13] Tackling road traffic injuries was a priority in the government's Health Sector Strategy 2015-2020. [14] A National Road Safety Action Plan 2013-2020 [15] was

For peer review only - http://bmjopen.bmj.com/site/about/guidelines.xhtml

acknowledged but not ratified by Parliament. Neither document specified the research required to support the delivery of improved road safety.

In order to improve road safety, coordinated efforts are needed across the road transport system. Research is vital to optimise decision-making. Current initiatives in Nepal for the control and prevention of road traffic crashes and their consequences are not based on local evidence. Therefore, this study aimed to -- involve a wide range of experts and participants representing stakeholder organisations to identify the research needed to help agencies in Nepal develop a safe systems approach to road safety, and to achieve a consensus about which studies should be prioritised.

METHODS

This study used the Delphi approach [16-18] to develop a consensus on a prioritised list of road safety research questions. Five groups of stakeholders in Nepal were engaged. The roles and experience of participants were relevant to each of the five WHO pillars of road safety. The study was conducted in two stages: firstly, interviews were conducted with stakeholders to identify a range of possible research questions, and secondly, participants completed two rounds of ranking the research questions in order of importance. Each of the five road safety pillars was studied separately. Five interview topic guides were developed in the Nepali language, based on the activities recommended for each of the five WHO pillars of road safety (Supplementary file 1).

Recruitment of the participants

Potential study participants were identified through existing networks and multi-sector stakeholder groups on road safety and first response. Networks included third sector and advocacy organisations for road safety. Participants helped identify further potential participants through a snowballing approach. We aimed to recruit 20-25 participants for each of the five pillars. Potential participants were contacted by telephone and were provided with information about the study and their interest in our research was confirmed. For participants expressing an interest, written information regarding the study and a consent form were sent to the potential participants via email. All the recruitment took place during the Covid-19 pandemic and therefore most of the interviews were completed remotely, by phone or videocall. For these participants, consent was recorded verbally at the start of the interview, or was collected prior to participation via email. Later in the pandemic it became feasible to engage some participants face-to-face. For these participants consent was collected at this meeting.

For peer review only

Data collection and analysis

In Round 1, we conducted interviews with participants in which we asked what additional data or information would help them in their job and reduce road traffic injuries. We explored the barriers they faced when tackling road safety. Most of the interviews were conducted using online platforms such as MS Teams, Zoom, Google Meet or Viber, and some interviews were conducted over the telephone. Towards the end of the data collection period, and when Covid-19 pandemic restrictions allowed, we conducted a small number of face-to-face interviews where this was the preference of the participants. In these circumstances, mitigations against infection, such as social distancing and the wearing of face masks, helped protect both participants and researchers. Interviews were conducted in Nepali language and audio-recorded. Audio recordings were listened to several times. Information relating to perceived gaps in research or evidence was documented as potential research questions on a spreadsheet, in English. For each group of stakeholders, approximately 200 research suggestions were generated from the interviews. Many of the participants raised similar issues, therefore it was possible to cluster the questions into groups, and to formulate a single question to represent that area of research need. The grouping stage was completed collaboratively by the whole research team to ensure that questions were treated equally and the process consistently applied. A reduced list of about 30 questions was achieved, identifying the research and evidence needs relating to each pillar of road safety.

For Round 2, the research questions from the reduced list were uploaded to an online survey tool (Qualtrics) in both English and Nepali languages. The link to the survey was distributed to the participants via email or Viber message. Participants were asked to give their opinion on the importance of each research question using a 5-point Likert scale: *Not Important*, *Low Importance*, *Moderate Importance*, *Important*, and *Very Important*. Reminders to complete the survey were sent via email and individual phone calls after one week and followed up again 2-3 days later. Completed surveys were exported from Qualtrics and analysed in MS Excel. Survey results were collated to identify the number of participants who rated each question as "important" or "very important". Questions where a significant majority of participants had scored them 'important' or 'very important' were retained as prioritised questions. For Pillars 1, 3, 4 and 5 we retained questions where ≥70% of the participants rated the questions as "important" or "very important". For Pillar 2 we retained questions where ≥80% participants rated at these levels, since a greater proportion of the questions were considered important. We used these threshold values based on published Delphi studies. [19 20]

For peer review only - http://bmjopen.bmj.com/site/about/guidelines.xhtml

For Round 3, participants were invited to a real-time online workshop where the prioritised questions were presented and discussed. The workshop was designed to provide the participants the opportunity to share their views and listen to each other's opinions regarding which issues were the most important to research. These workshops were recorded and shared with those who were not able to join. Following the workshop, a Qualtrics survey was sent to all participants again, this time listing only those questions prioritised from Round 2. Participants were again asked to score each question as either *Not Important*, *Low Importance*, *Moderate Importance*, *Important*, or *Very Important*. Reminders were sent to the participants after one week and followed up again after 2-3 days. Completed surveys were exported to MS Excel and collated to identify the number of participants considering each question 'important' or 'very important'. This resulted in the final prioritised list of research questions for each pillar of road safety.

The research team completed Rounds 1, 2 and 3 for one pillar before moving on to the next pillar. The interviews started on 12 July 2020 and were completed on 14 February 2021. Due to the Covid-19 pandemic, where government officials and clinical staff were not easily available to participate, stakeholders in Pillars 1 and 5 were left until later in the study when the peak of the first wave of Covid-19 in Nepal had passed.

Overarching consensus workshop

A final online consensus workshop was organised where the top ranked research questions from all five pillars were shared with all the participants, stakeholders from our advisory groups and invited key decision makers. A facilitated discussion explored the understanding of what the different research options could provide and how that new evidence could potentially be used. Using online voting software (Mentimeter, https://www.menti.com), participants were encouraged to vote for one research question from each pillar that they considered needed to be addressed the most urgently. The questions considered most urgent were presented back to the group.

Ethical approval for conducting this study was obtained from the Kathmandu Medical College Institutional Review Committee (ref. 040620201) and the University of the West of England Bristol Faculty Research Ethics Committee (ref. HAS. 20.06.192).

Patient and public involvement

For peer review only - http://bmjopen.bmj.com/site/about/guidelines.xhtml

Through community engagement and involvement we engaged individuals with diverse views on road safety, ranging from road users through to those with decision making authority for road development, management and traffic regulation.

RESULTS

Description of study participants

Out Of 133 potential participants identified and contacted, 93 individuals were recruited and took part in 95 interviews covering all five road safety pillars (two participants had expertise relevant to more than one pillar, and therefore took part in two interviews each). Participants were from a range of organisational and professional backgrounds, including government institutions, academia, road safety engineers, clinicians, civil society organisations, and all had an interest or remit that addressed one or more of the five pillars of road safety. Some of the experts in our list, when contacted, suggested the name of other stakeholders. Out of 93 participants, 83 were from Kathmandu valley and represented organisations with the remit to work or influence road safety nationally. Ten participants were from outside Kathmandu and added value to the study by providing local contexts. The participants' background characteristics are summarised in Table 1.

Table 1. Organisational/professional background of the participants

Organisational / professional background	Total	Male	Female
Government organisation (Secretaries, Govt Officers, Police, Political representatives)	33	30	3
Clinician, Nurse, physiotherapist	10	8	2
Road safety Engineer	9	9	0
Road Safety Advocacy	8	5	3
Academics	7	6	1
First Aid/ emergency/ ambulance provider	6	6	0
Engineers' Association	4	4	0
Transport worker	4	4	0
Automobile dealer	3	3	0
Federation of transport	2	2	0
Schools' organisation	2	2	0

For peer review only - http://bmjopen.bmj.com/site/about/guidelines.xhtml

Sustainable Transport	2	2	0
Others (journalist and city planners)	3	3	0
**Total**	**93**	**84**	**9**

Across all five pillars we identified a total of 1019 research suggestions from the 95 interviews completed in Round 1. Collating similar questions reduced this to 141 questions across the five pillars. Seventy-six (80%) participants took part in Round 2, through which the list of questions was reduced to 91 questions. Forty (43%) participants took part in an online workshop prior to further ranking in Round 3 which was completed by 64 (69%) participants and resulted in a total of 30 prioritised questions. Figure 1 shows the stages of the Delphi study and the number of participants in each round. Attrition of participants was greatest for the group discussing Pillar 1 (road safety management), where 10/21 (48% participants) dropped out between Round 1 and Round 3. Attrition was least in the group discussing Pillar 2 (safer roads) where only 3/18 (17%) of participants were lost.

Figure 1. Flow chart of the Delphi process

The high attrition of participants in Pillar 1 was not unexpected since many of these participants worked in government positions and it was difficult for them to prioritise attendance during the Covid-19 pandemic. Figure 2 illustrates participant attrition throughout the study.

Figure 2. Study participants retained in subsequent Delphi rounds, by pillar.

Table 2 describes the number of research questions prioritised in each Round, split by the pillars of road safety. The retention rate in this study was equivalent to that in other published Delphi studies [21] despite the Covid-19 pandemic.

Table 2. Research questions prioritised at each Round, by pillar

**Pillar of road safety**	**Round 1**				**Round 2**	**Round 3**
	**Interview dates**	**Number of interviews (online or by phone)**	**Research questions generated ('long list')**	**Grouped research questions ('reduced list')**	**Number of 'Important' or 'very important' research**	**Number of questions considered most important**

For peer review only

					questions (above 70% consensus)	(Top 5 ranks)
Pillar 1 (Road safety management)	23 Nov to 22 Jan 2021	21 (21)	183	25	17	5
Pillar 2 (Safer roads and mobility)	13 July to 12 Aug 2020	18 (4)	211	30	19*	5
Pillar 3 (Safer vehicles)	16 Aug to 15 Sep 2020	17 (17)	217	30	20	7
Pillar 4 (Safer road users)	23 Sept to 19 Oct 2020	20 (20)	178	30	20	6
Pillar 5 (Post-crash response)	05 Jan to 14 Feb 2021	19 (13)	230	26	15	7
Total		95 (75)	1019	141	91	30

*80% consensus

The top ranked research questions for the five pillars of road safety are presented in Table 3. The research questions that were considered the most important cover a wide range of issues, including how to make existing processes more effective, how to assess the training needs of the road safety workforce, understanding the challenges of implementing existing road safety legislation, how to improve accountability for road safety, how to generate and disseminate better information to inform decisions, and how to generate evidence that supports the economic argument for road safety.

Table 3 List of top questions for Pillars 1 to 5 with scores in rounds 2 and 3

	Scores*	
**Pillar 1: Road Safety Management**	**R2**	**R3**

For peer review only - http://bmjopen.bmj.com/site/about/guidelines.xhtml

How can implementing agencies be made more accountable for road safety in urban and rural areas?	92	91
What are the barriers to conduct road safety audits at all stages of road construction and implementation of their recommendations?	85	91
How can urban and rural roads construction and management be governed to ensure improved road safety?	92	91
How can the traffic management system be improved to ensure it improves the safety of all road users?	85	91
What are the barriers to implementation of existing laws related to road safety in Nepal?	69	91
**Pillar 2: Safer Roads**		
What is the effectiveness of different safety features installed on roads in terms of crash reduction?	94	100
What are the barriers and facilitators for achieving safer roads in Nepal?	88	100
What kind of institutional setup is needed at central, provincial and local levels for the promotion of road safety ownership and accountability?	94	93
What are the economic benefits of installation of safety features during road construction, regular maintenance and upgrading of roads?	82	93
How should different types of roads, and roads in different geographical locations, be designed to make them safer for all road users?	82	93
**Pillar 3: Safer vehicles**		
What are the factors affecting fitness condition and road worthiness of vehicles to the extent that it leads to road traffic crashes?	86	100
What should be the minimum criteria for the establishment of standard vehicular maintenance workshops?	93	92
What are the capacity development and training needs for currently working human resources and additional jobs to improve the safety of vehicles in Nepal?	71	92
What improvements in policies and institutional setup is needed to ensure vehicle safety of all types and routes?	79	92
What is the role of motor parts used for vehicle maintenance for fitness condition of the vehicles and road crashes?	93	83
How does overloading impact safety of the vehicles?	71	83
What are the vehicle related factors causing road crashes in Nepal?	71	83
**Pillar 4: Safer Road users**		

For peer review only

How can the driver licencing system be made more effective in order to ensure safer vehicle drivers?	100	93
What are the main factors increasing the risk of public vehicle crashes? What interventions would improve the safety of travel on public vehicles?	94	93
How can licensing and crash data collection systems be improved?	94	93
What are the major causes of road crashes in Nepal? What percentage of road crash is due to unsafe road user behaviours?	94	87
What content should be included in awareness campaigns for different types of road user, and how are these campaigns best delivered?	83	87
What are the barriers in the implementation of laws regarding safer road user behaviour? Review of existing policies related to safer road users.	78	87
**Pillar 5: Post-crash response**		
What standards should be applied to ambulance services? (includes standards for personnel and training, equipment carried, and the vehicles)	100	100
What is the standard of care at health centres and hospitals for road traffic injury patients across the country, and how can they be improved?	79	92
What is the current average time taken for a road traffic injury patient to receive first response at the scene and the average time taken to arrive at a healthcare setting able to meet their care needs? How can any delays be reduced?	93	92
What factors influence the ability of the post-crash emergency response service to get to the patient and then get them to the right hospital in the best possible time?	86	92
What should be included in the training curriculum for the different levels of post-crash responders?	93	85
How should policies and legislation be further developed to support the post-crash response for road traffic injury victims?	71	85
What is the optimal model of insurance to minimise death & disability following a road traffic crash? What are the barriers & facilitators to implementing such an insurance system?	71	85

*Percent of participants ranked "very important" or "important"; R2= Round 2; R3= Round 3.

Note: The phrasing of questions presented in this table reflects the direct translation from Nepali to English of the research questions used in the ranking process.

A total of 56 people (47 participants and 9 key decision makers) attended the workshop conducted at the end of the study where the list of the top ranked research questions for each of the five pillars

For peer review only - http://bmjopen.bmj.com/site/about/guidelines.xhtml

were presented. Using electronic voting software to identify the question within each Pillar considered to be the most urgent, 6 questions were prioritised. Two questions in Pillar 4 were scored equally (Table 4).

Table 4. Top 6 most urgent research questions

Pillars	Research Questions
Pillar 1	How can implementing agencies be made more accountable for road safety in urban and rural areas?
Pillar 2	How should different types of roads, and roads in different geographical locations, be designed to make them safer for all road users?
Pillar 3	What are the factors affecting fitness condition and road worthiness of vehicles to the extent that it leads to road traffic crashes?
Pillar 4	How can the driver licensing system be made more effective in order to ensure safer vehicle drivers?
	What are the main factors increasing the risk of public vehicle crashes? What interventions would improve the safety of travel on public vehicles?
Pillar 5	What factors influence the ability of the post-crash emergency response service to get to the patient and then get them to the right hospital in the best possible time?

For peer review only

DISCUSSION

This study is the first to our knowledge that has engaged such a wide group of participants to identify the research priorities relevant to the improvement of road safety in Nepal. The research team was able to identify and approached 133 potential participants and 70% (93 people) agreed to take part. Respondents included stakeholders from a range of organisational and professional backgrounds as well as geographical areas and included; officials in government institutions (Ministerial Secretaries, Government Officers, Police, Political representatives), clinicians, nurses, physiotherapists, engineers, academics, first responders, transport workers, automobile dealers, road users, members of the media and city planners. The proportion of women working in roles related to road safety in Nepal is known to be low, and we were pleased to have been able to recruit 9/93 (10%) female participants,

For peer review only - http://bmjopen.bmj.com/site/about/guidelines.xhtml

which is in line with official data on the Nepali work force. The number of participants that should take part in a Delphi study is not prescribed and it can be anywhere above 10 persons; the number is guided by the scope of the problem and existing resources. [19 20] Overall, the retention of the participants until the third round of ranking was excellent however, rates varied between different pillars. The overall retention rate of 69% and 50% attendance at the final consensus workshop indicated the high level of interest in road safety research in Nepal. This rate is higher than that reported by Marchau and Van der Heijden [22] in a multi-country road safety study. Marchau and Van der Heijden [22] applied the Delphi technique to explore the policy aspects of implementing driver support systems. The authors used a questionnaire with specified answer options sent to international experts from USA, Japan and Europe. In this study, 56% (65 out of 117) of invitees responded in the first round while only 40 responded in the third round. Compared to these rates,participation in our study was good.

Road safety research is a neglected issue in low- and middle- income countries [23] and a lack of research capacity may be one reason for the limited progress to date. In Nepal, a policy review identified that institutional arrangements and resource allocation for road safety were inadequate. [24] The lack of coordination of road safety sectors is a challenge globally [25 26] as well as in Nepal. Many of the participants in this study had the opportunity to meet and discuss road safety with those working in other sectors, for the first time.

Other studies exploring aspects of road safety through the use of the Delphi technique have mostly come from high income countries, with the exception of a few, such as Vietnam and Uganda. Studies have explored specific risk factors such as cell phone use and sleep deprivation in the USA, [27 28] and public bus safety in Italy. [29] Some studies focussed on the need to improve post-crash care such as; strengthening trauma management in Vietnam, [30] pre-hospital emergency care in Iran, [31] post-recovery rehabilitation in Australia, [32] and emergency medical services capacity in Uganda. [33] In Iran, Delphi studies have been conducted to inform the development of minimum datasets to study road crashes, [34] and developing a national road safety education programme. [35] We have not identified any previously published Delphi studies that have included all five pillars of road safety in a single study.

Zhu, et al. [27] recruited road safety experts and young drivers in the USA to study the risks of mobile phone use while driving. Expert participants identified texting, sending email or picking up the phone as particularly high risk behaviours for crashes, but not playing music on a handheld mobile which was prioritised by young drivers. Participants identified 20 behavioural practices related to mobile

For peer review only - http://bmjopen.bmj.com/site/about/guidelines.xhtml

use which can result in a collision. Our study participants in Pillar 4 also identified the importance of studying causes of driver distraction, but did not identify mobile phone use in particular.

Cafiso, et al. [29] engaged the managers of large public bus companies in Italy in a Delphi study to explore bus safety. Participants rated safety solutions for issues relating to driver behaviour, traffic conflicts and vehicle maintenance and technology. Our study participants also raised concerns about the safety of public transport users and the safety of public passenger vehicles, and prioritised a study to investigate the factors contributing to public vehicle crashes. The technological solutions explored in the study by Cafisco (e.g. technology to control when the bus can start, automatic door closing etc.) are not applicable in the context of Nepal where public passenger vehicles are older and poorly equipped. An expert panel on sleep deprivation in a study by Czeisler, et al. [28], agreed that a driver was not fit to drive if they had less than 2 hours sleep in the previous 24 hours. In our study participants raised concerns regarding driver behaviour, including fatigue but prioritised a study to review the entire driver licensing system rather than focussing on tackling specific driver behaviours. These examples illustrate how previous Delphi studies have tended to focus on specific road safety issues, and how the results are specific to the context or participants. Neither of these studies would be directly generalizable to Nepal, nor do they cover the breadth of safety issues identified in our study.

Several Delphi studies have reported post-crash trauma management and prehospital care. In Vietnam, Schmucker, et al. [30] used online meetings followed by a questionnaire survey of 1000 road users to generate responses that were ranked and outcomes were used to inform the development of a trauma care course. Our study participants for Pillar 5 also prioritised the development of training curricula for different levels of post-crash trauma care when ranking (table 3). Recently, Azami-Aghdash, et al. [31] used the Delphi technique to achieve a consensus on 37 indicators to measure and improve the performance of prehospital care following road crashes in Iran. This is similar to the topic prioritised for post-crash response (Pillar 5) in our study. However, the differences in Iranian and Nepali country contexts and pre-hospital care infrastructure mean that performance indicators in Iran are not generalizable to Nepal. Balikuddembe, et al. [33] used the Delphi technique to identify and prioritise factors that could prevent and support victims of road traffic injuries in Kampala. They identified 23 factors across the entire Emergency Medical Service system that were similar to issues raised by participants in Pillar 5 of our study.

In the course of our study, shifts in the opinions of participants were observed during Rounds 2 and 3. In relation to the rankings completed in Round 2, a high degree of consensus was observed and

For peer review only - http://bmjopen.bmj.com/site/about/guidelines.xhtml

For peer review only

the process creating a reduced list for Round 3 was relatively straightforward. The Delphi method dictates that the results of a first round be re-presented to participants in subsequent Rounds, giving participants the opportunity to reconsider their views in the light of discussion, additional thought and/or the results obtained from other participants. [20 36] Cafiso, et al. [29] in their study, similarly reported that after the second Round, the Delphi panellists' opinions were influenced by those of their colleagues. In our study, the changed ranks of the questions between Round 2 and Round 3 illustrate the value and influence of discussion between Rounds in reaching a consensus view. High numbers of research questions were rated 'important' or 'very important' in our study, illustrating that many participants recognised the need for road safety research in Nepal. Issues relating to improving the safety of road users traditionally considered vulnerable (e.g. pedestrians, cyclists, drivers and passengers of powered two wheelers) were raised by participants in this study, however, during ranking, research questions that improved the safety of all road users were prioritised over questions relating to these specific groups.

The Government of Nepal plans to enact a Road Safety Bill [37] that will include issues relating to planning, resourcing, implementation, and evaluation of national road safety activities. Provincial Governments, which were established only 4 years ago, through the promulgation of the constitution of Nepal, [38] have started to enact Provincial Transport Management Acts. However, the institutional structures necessary to implement these laws are still in development. [24] The research questions prioritised in this study emphasise the need for evidence to support both national development plans [8] and safer roads and transport in Nepal. [39] Existing road safety policies are mostly only partially implemented. [24] Policy gaps include policies to separate traffic and road users and those to address speed management.

Strengths and limitations

High response rate (69%), representation and involvement of most of the individuals and experts currently active in the fields of road construction, vehicle management, transport management and post-crash response is a major strength of this study. The Delphi method for achieving consensus is a research technique with the potential for biases; [20] Hallowell [17] outlined common biases in implementation and here we describe the measures applied to minimise these biases in the current study. To minimize factors that might influence the quality of the conclusions due to the level of expertise of the panel members, [40] only experienced and recognised authorities working for road safety in Nepal were invited to participate. The results produced by Delphi studies are often thought to be limited due to poor quality of the facilitator's survey instruments, [16] therefore, the tools

For peer review only - http://bmjopen.bmj.com/site/about/guidelines.xhtml

developed for this study were informed by the international literature and advice was available from an experienced Delphi expert. Bias can occur if questions are poorly worded [17] therefore our researchers were trained in interviewing skills prior to commencing Round 1 and conducted the interview in Nepali. Some critics believe that convergence of opinion in Delphi studies is conformity. [18] To counter this risk, we synthesised best global road safety practice as reported in published literature and presented this to participants during the workshops between Rounds 2 and 3. Although the Delphi approach has been reported to be overly time intensive, [41] we found the time taken to participate did not inhibit a high proportion of participants to remain in the study to its conclusion.

CONCLUSIONS

This study identified research priorities for road safety in Nepal across all the WHO's five pillars of road safety. The most urgent and important research questions related to; improving the governance of road safety through greater accountability, improving road design across different topographies, establishing the contribution of poor vehicle fitness to crash occurrence, strengthening the driver licencing system, improving the safety of passengers on public buses, and understanding the barriers to the provision of effective post-crash care. These findings can guide researchers when designing future studies and the study provided opportunities for stakeholders across sectors in Nepal to meet and debate issues together. Future research has the potential to lead to evidence-informed policy development and implementation, and improved practices relating to road construction and management, vehicle standards, and post-crash care, making the roads safer for all road users in Nepal.

Acknowledgements: We would like to acknowledge the support of Professor Nichola Rumsey who provided training in Delphi study methodology to the study team, advised on the study protocol and this manuscript. We are grateful to all 93 expert participants without whom this study would not have been possible.

Contributors: Puspa Raj Pant: Data curation; Project administration; Original draft. Pranita Rana: Data collection; Investigation; Project administration; Validation; review & editing. Kriti Pradhan: Data collection; Investigation; Project administration; Validation; review & editing. Sunil Kumar Joshi: Project administration; Resources; Supervision; review & editing. Julie Mytton: Conceptualization; Data curation; Funding acquisition; Methodology; Supervision; review & editing.

For peer review only - http://bmjopen.bmj.com/site/about/guidelines.xhtml

Funding: This research was commissioned by the National Institute for Health Research (NIHR) Global Health Policy and Systems Research Development Award using UK aid from the UK Government (NIHR129877). The views expressed in this publication are those of the author(s) and not necessarily those of the NIHR or the Department of Health and Social Care.

Competing interest: the authors have no competing interests to declare

Data availability statement: Data are available upon reasonable request.

For peer review only

References:

1. World Health Organization. Global status report on road safety 2018: World Health Organization 2018.
2. World Bank. Guide for Road Safety Opportunities and Challenges: Low- and Middle-Income Countries Country Profiles. . Washington DC, USA: World Bank, 2019.
3. Peden M, Scurfield R, Sleet D, et al. World report on road traffic injury prevention: World Health Organization Geneva, 2004.
4. WHO. Save LIVES - A road safety technical package. Geneva: World Health Organization, 2017.
5. World Road Association. The Safe System Approach. In: Kienreich R, Milton J, eds. Road Safety Manual: A manual for practitioners and decision makers on implementing safe system infrastructure: World Road Association (PIARC), 2019.
6. WHO. Global plan for the decade of action for road safety 2011-2020. Geneva: World Health Organization, 2011.
7. Ministry of Finance. Economic Survey 2020/21. Ministry of Finance, Kathmandu, 2021.
8. National Planning Commission. The fifteenth plan (FY 2019/20 to 2023/24) unofficial English translation. Kathmandu: Government of Nepal, 2020.
9. McAdoo BG, Quak M, Gnyawali KR, et al. Roads and landslides in Nepal: how development affects environmental risk. *Natural Hazards and Earth System Sciences* 2018;18(12):3203-10.
10. Department of Transport Management. Statistical details of vehicles registered in Nepal up to FY 2017/18 [Online]. Kathmandu2020 [Statstics of registered vehicles in Nepal]. Available from: https://www.dotm.gov.np/Files/NoticePDF/vehiclesdatatill2074-752020-01-17_04-49-13-732.pdf accessed 24 May 2021.

For peer review only - http://bmjopen.bmj.com/site/about/guidelines.xhtml

11. Ministry of Health and Population. HMIS Database 2074/75 by Local Government Kathmandu: Department of Health Services; 2021 [updated 25 March 2020. Available from: https://dohs.gov.np/ihims-raw-data/ accessed 1 April 2021.

12. Ojha KN. Road safety status and some initiatives in Nepal. *ITEGAM-JETIA* 2021;7(27):20-40.

13. Pant PR, Mytton J, Dharel MR, et al. The prevention of–and first response to–injuries in Nepal: a review of policies and legislation. *Health research policy and systems* 2021;19(1):1-20.

14. Ministry of Health and Population. Nepal Health Sector Strategy 2015-2020 Kathmandu: Ministry of Health and Population, Government of Nepal, 2015.

15. Goverment of Nepal. Nepal Road Safety Action Plan (2013 - 2020): Draft. Kathmandu, Nepal: Department of Road, Goverment of Nepal, 2011.

16. Gupta UG, Clarke RE. Theory and applications of the Delphi technique: A bibliography (1975–1994). *Technological Forecasting and Social Change* 1996;53(2):185-211. doi: https://doi.org/10.1016/S0040-1625(96)00094-7

17. Techniques to minimize bias when using the Delphi method to quantify construction safety and health risks. Construction Research Congress 2009: Building a Sustainable Future; 2009.

18. Rowe G, Wright G. The Delphi technique: Past, present, and future prospects—Introduction to the special issue. *Technological forecasting and social change* 2011;78(9):1487-90.

19. Hasson F, Keeney S, McKenna H. Research guidelines for the Delphi survey technique. *Journal of advanced nursing* 2000;32(4):1008-15.

20. Powell C. The Delphi technique: myths and realities. *Journal of Advanced Nursing* 2003;41(4):376-82. doi: https://doi.org/10.1046/j.1365-2648.2003.02537.x

21. Hamlet C, Rumsey N, Williamson H, et al. Consensus research priorities for facial palsy: A Delphi survey of patients, carers, clinicians and researchers. *Journal of Plastic, Reconstructive & Aesthetic Surgery* 2018;71(12):1777-84.

22. Marchau V, Van der Heijden R. Policy aspects of driver support systems implementation: results of an international Delphi study. *Transport Policy* 1998;5(4):249-58.

23. Perel P, Ker K, Ivers R, et al. Road safety in low-and middle-income countries: a neglected research area. *Injury Prevention* 2007;13(4):227-27.

24. Pant PR, Mytton J, Dharel MR, et al. The prevention of – and first response to – injuries in Nepal: a review of policies and legislation. *Health Research Policy and Systems* 2021;19(1):65. doi: 10.1186/s12961-021-00686-1

25. Bliss T, Breen J. Meeting the management challenges of the Decade of Action for Road Safety. *IATSS research* 2012;35(2):48-55.

For peer review only

For peer review only - http://bmjopen.bmj.com/site/about/guidelines.xhtml

26. Torbaghan ME, Sasidharan M, Burrow M, et al. Lessons from road safety research and policy engagement in Pakistan, Nepal, Tanzania. 2019

27. Zhu M, Rudisill TM, Rauscher KJ, et al. Risk perceptions of cellphone use while driving: Results from a delphi survey. *International journal of environmental research and public health* 2018;15(6):1074.

28. Czeisler CA, Wickwire EM, Barger LK, et al. Sleep-deprived motor vehicle operators are unfit to drive: a multidisciplinary expert consensus statement on drowsy driving. *Sleep health* 2016;2(2):94-99.

29. Cafiso S, Di Graziano A, Pappalardo G. Using the Delphi method to evaluate opinions of public transport managers on bus safety. *Safety science* 2013;57:254-63.

30. Schmucker U, Ottersbach C, Frank M, et al. A new approach and first steps to strengthen trauma management and road safety in North Vietnam. *Journal of trauma management & outcomes* 2008;2(1):1-7.

31. Azami-Aghdash S, Moosavi A, Gharaee H, et al. Development of quality indicators to measure pre-hospital emergency medical services for road traffic injury. *BMC health services research* 2021;21(1):1-12.

32. Smits EJ, Gane EM, Brakenridge CL, et al. Expert consensus and perspectives on recovery following road traffic crashes: a Delphi study. *Disability and rehabilitation* 2020:1-10.

33. Balikuddembe JK, Ardalan A, Khorasani-Zavareh D, et al. Factors affecting the exposure, vulnerability and emergency medical service capacity for victims of road traffic incidents in Kampala Metropolitan Area: a Delphi study. *BMC emergency medicine* 2016;17(1):1-8.

34. Mohammadi A, Ahmadi M, Gharagozlu A. Developing a minimum data set for an information management system to study traffic accidents in Iran. *Iranian Red Crescent Medical Journal* 2016;18(3)

35. Bakhtari-Aghdam F, Sadeghi-Bazargani H, Azami-Aghdash S, et al. Developing a national road traffic safety education program in Iran. *BMC public health* 2020;20(1):1-13.

36. Williamson K. The Delphi Method. Research methods for students, academics and professionals: Information management and systems. Second ed. New South Wales: Kirsty Williamson 2002.

37. RSS Nepal. Call to pass bill related to road safety. *The Himalayan Times* 2019 3 Feb 2019.

38. Government of Nepal. The Constitution of Nepal. Kathmandu, 2015.

39. Government of Nepal. Vehicle and Transportation Management Act 1993 (Ammendment 2019). Kathmandu, 1993.

For peer review only - http://bmjopen.bmj.com/site/about/guidelines.xhtml

40. Veltri AT. Expected use of management principles for safety function management. West Virginia University, 1985.

41. Rajendran S. Sustainable construction safety and health rating system. 2006

Legends

Figure 1. Flow chart of the Delphi process

Figure 2. Study participants retained in subsequent Delphi rounds, by pillar.

For peer review only

For peer review only - http://bmjopen.bmj.com/site/about/guidelines.xhtml

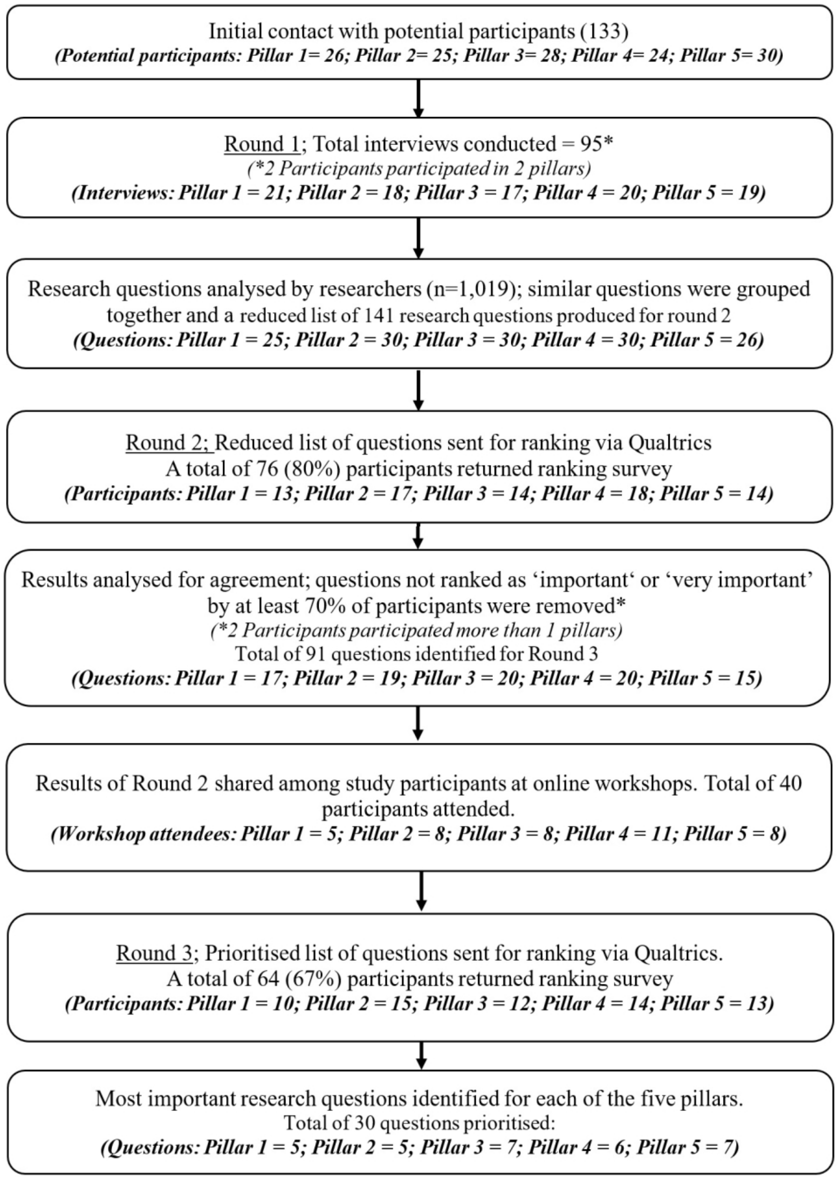

Figure 1. Flow chart of the Delphi process

376x535mm (96 x 96 DPI)

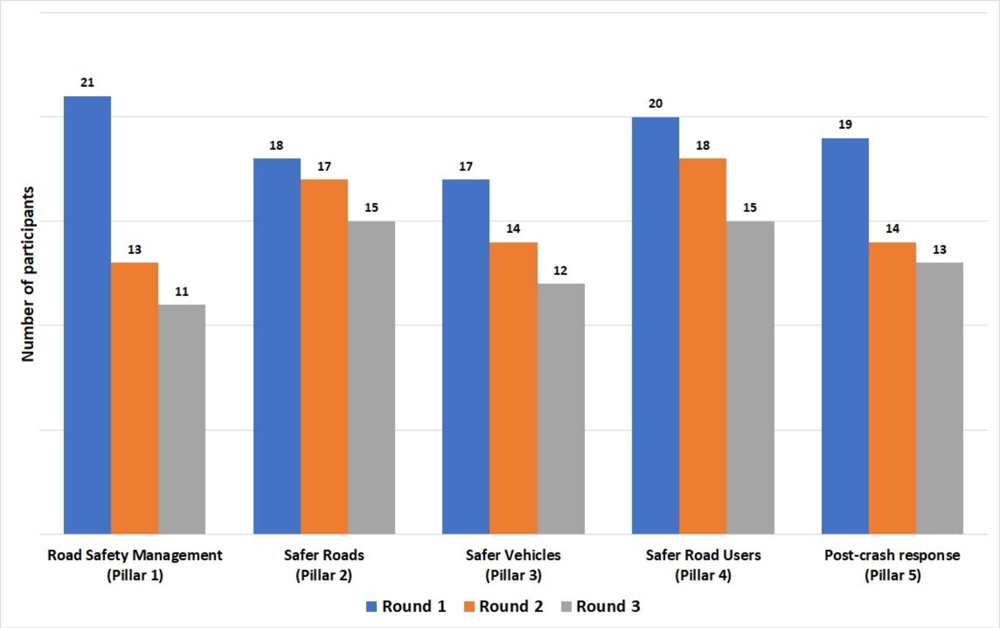

Figure 2. Study participants retained in subsequent Delphi rounds, by pillar.

302x189mm (96 x 96 DPI)

Interview guide

Road Safety Research Prioritisation study

Pillar 1: Road safety management

(To be read to each participant for this pillar) *This pillar focuses on strengthening multi-agency capacity for road safety. It includes activities such as putting into practice major UN road safety conventions, establishing a multi-sectoral national agency to lead road safety activities, developing a national road safety strategy and setting realistic and long-term targets for related activities with sufficient funding for implementation. It also calls for the development of data systems to effectively monitor and evaluate activities.*

For peer review only

Questions and prompts

- What is your job title and what is the focus of your responsibilities for this position?
- How long have you been in this role?
    - Prompt: How these responsibilities are determined?
- From the description of Pillar 1, what is the status of activities for this pillar in Nepal?
- From your experience, what is going well?
    - *Prompt: Why do you think it is going well?*
- From you experience what has not yet happened or is not working well?
    - *Prompt: Why do you think it is not working well?*
- What challenges are you facing to achieve your desired objectives for road safety?
    - *Prompts:*
        - *challenges regarding to have a lead agency?*
        - *challenges regarding national strategy?*
        - *challenges regarding data generation?*
        - *challenges regarding monitoring and evaluation?*
- What information or evidence do you think would help you achieve these objectives?
- Can you think of any gaps in the research available to you, related to these activities in pillar 1?
- Would you like to add anything which we have not covered during this conversation?

At the end of the interview

- Explain that this is the end of the interview.
- Thank the participant for their time.
- Explain that the information they have given will be used to create a list of possible research ideas to improve road safety in Nepal.
- Explain that they will be invited to the next stage of the study where they will hear all of the research questions we have identified, and they will be invited to tell us which ones they think are the most important.
- Ask if they have any questions before you go.

For peer review only - http://bmjopen.bmj.com/site/about/guidelines.xhtml

Pillar 2. Safer roads and mobility

(To be read to each participant for this pillar) *This pillar highlights the need to improve the safety of road networks and infrastructure for the benefit of all road users, including the pedestrians, bicyclists and motorcyclists. Activities include considering safety during the planning, design, construction and operation of roads; making sure that roads are regularly assessed for safety; and encouraging the relevant authorities to consider all forms of transport and types of safe infrastructure when they respond to the mobility needs of road users.*

Suggested questions and prompts

- What is your job title and what is the focus of your responsibilities for this position?
- How long have you been in this role?
  - Prompt: How these responsibilities are determined?
- From the description of Pillar 2, what is the status of activities for this pillar in Nepal?
- From your experience, what is going well?
  - *Prompt: Why do you think it is going well?*
- From you experience what has not yet happened or is not working well?
  - *Prompt: Why do you think it is not working well?*
- What challenges you are facing to achieve your desired objectives for road safety?
  - *Prompts*
    - *challenges to promote road safety ownership and accountability?*
    - *challenges promoting (addressing) the needs of all road users?*
    - *challenges relating to designing, building or maintaining roads?*
- What information or evidence do you think would help you achieve these objectives?
- Can you think of any gaps in the research available to you, related to these activities?
- Would you like to add anything which we have not covered during this conversation?

At the end of the interview

- Explain that this is the end of the interview.
- Thank the participant for their time.
- Explain that the information they have given will be used to create a list of possible research ideas to improve road safety in Nepal.
- Explain that they will be invited to the next stage of the study where they will hear all of the research questions we have identified and they will be invited to tell us which ones they think are the most important.
- Ask if they have any questions before you go.

Pillar 3. Safer vehicles

(To be read to each participant for this pillar) *Poor vehicle standards contribute to a significant number of crashes and casualties. This pillar encourages use of best practice vehicle safety standards and technology to promote safety. Activities may include implementing new car assessment programmes (such as NCAP safety ratings) and vehicle safety checks on existing vehicles to ensure they are equipped with minimum safety features, such as seat-belts to minimise the impact of crashes to occupants, and working lights and brakes.*

Suggested questions and prompts

- What is your job title and what is the focus of your responsibilities for this position?

For peer review only - http://bmjopen.bmj.com/site/about/guidelines.xhtml

- How long have you been in this role?
    - Prompt: How these responsibilities are determined?
- From the description of Pillar 3, what is the status of activities for this pillar in Nepal?
- From your experience, what is going well?
    - *Prompt: Why do you think it is going well?*
- From you experience what has not happened or is not working well?
    - *Prompts*
        - *Why do you think it is not working well?*
        - *What are your views on the New Car Assessment Programme (NCAP)?*
        - *do we have good vehicle-related laws that could promote the import of safer vehicles or the maintenance of existing vehicles?*
- What challenges you are facing to achieve desired objectives for road safety?
        - *challenges to harmonise international motor vehicle safety regulations with national laws?*
        - *research about safety technologies designed to reduce risk to vulnerable road users.*
- What information or evidence do you think would help you achieve these objectives?
- Can you think of any gaps in the research available to you, related to these activities?
- Would you like to add something else which we have not covered during this conversation?

At the end of the interview

- Explain that this is the end of the interview.
- Thank the participant for their time.
- Explain that the information they have given will be used to create a list of possible research ideas to improve road safety in Nepal.
- Explain that they will be invited to the next stage of the study where they will hear all of the research questions we have identified and they will be invited to tell us which ones they think are the most important.
- Ask if they have any questions before you go.

Pillar 4. Safer road users

(To be read to each participant for this pillar) *Pillar 4 focuses on developing comprehensive programmes to improve the behaviour of all road users. Activities include the adoption of model road safety legislation and sustained or increased enforcement or road safety laws and standards. These efforts are combined with public awareness and education to increase uptake of behaviours that keep people safe (e.g. seat-belt and helmet wearing) and to reduce behaviours that cause harm (e.g. speeding, taking alcohol or drugs when driving) and other risks. It also calls for activities to reduce work-related road traffic injuries and promoted the establishment of graduated driver licensing programmes for novice drivers.*

<u>Suggested questions and prompts</u>

- What is your job title and what is the focus of your responsibilities for this position?
- How long have you been in this role?
    - Prompt: How these responsibilities are determined?
- From the description of Pillar 4, what is the status of activities for this pillar in Nepal?
- From your experience, what is going well?

For peer review only - http://bmjopen.bmj.com/site/about/guidelines.xhtml

- *Prompt: Why do you think it is going well?*
- From you experience what has not happened or is not working well?
  - *Prompt: Why do you think it is not working well?*
- What challenges you are facing to achieve desired objectives for road safety?
  - *Prompts*
    - *what is the status of law enforcement?*
    - *what could be done to strengthen road safety law enforcement?*
    - *How good is the uptake of safe driver / passenger behaviours (e.g. seatbelt / helmet use)?*
    - *What role do driving licences play in road safety?*
    - *Are there any gaps in what we know about road user behaviours and how to change them?*
    - *what about gaps in legislation or how it is enforced relating to road users behaviour?*
- What new information or evidence do you think would help you to improve the uptake of safe road user behaviours?
- Can you think of any gaps in the research available to you, related to these activities in Pillar 4?
- Would you like to add something else which we have not covered during this conversation?

At the end of the interview

- Explain that this is the end of the interview.
- Thank the participant for their time.
- Explain that the information they have given will be used to create a list of possible research ideas to improve road safety in Nepal.
- Explain that they will be invited to the next stage of the study where they will hear all of the research questions we have identified and they will be invited to tell us which ones they think are the most important.
- Ask if they have any questions before you go.

Pillar 5. Post-crash response

(To be read to each participant for this pillar) *Pillar 5 addresses the need to improve the response to post-crash emergencies and the ability of health and other systems to provide appropriate emergency treatment and long-term rehabilitation for crash victims. The development and improvement of pre-hospital care systems, hospital trauma care systems, and rehabilitation along with long-term medical support to victims and a single emergency response number, are the main elements of post-impact care.*

Suggested questions and prompts

- What is your job title and what is the focus of your responsibilities for this position?
- How long have you been in this role?
  - Prompt: How these responsibilities are determined?
- From the description of Pillar 5, what is the status of activities for this pillar in Nepal?
- From your experience, what is going well?
  - *Prompt: Why do you think it is going well?*
- From you experience what is left behind or not working well?
  - *Prompt: Why do you think it is not working well?*

For peer review only - http://bmjopen.bmj.com/site/about/guidelines.xhtml

- What challenges you are facing to achieve desired objectives for road safety?
    - *Prompts*
        - *why there is no single nationwide telephone number for emergency services i.e. ambulances?*
        - *How do you see the performance of hospital trauma services in Nepal?*
- What new information or evidence do you think would help improve the provision of good post-crash response and care in Nepal?
- Can you think of any gaps in the research or information available to you in regard to the recommendations in Pillar 5?
- Would you like to add something else which we have not covered during this conversation?

At the end of the interview

- Explain that this is the end of the interview.
- Thank the participant for their time.
- Explain that the information they have given will be used to create a list of possible research ideas to improve road safety in Nepal.
- Explain that they will be invited to the next stage of the study where they will hear all of the research questions we have identified and they will be invited to tell us which ones they think are the most important.
- Ask if they have any questions before you go.

Below is the Nepali translation of the Guide.

For peer review only - http://bmjopen.bmj.com/site/about/guidelines.xhtml

अन्तरवार्ता निर्देशिका

नेपालमा सडक सुरक्षाका लागि अनुसन्धान प्राथमिकताहरू पहिचान गर्ने अध्ययन

स्तम्भ १. सडक सुरक्षा व्यवस्थापन

(To be read to each participant for this pillar)

सडक सुरक्षाको यो स्तम्भ देशमा सडक सुरक्षाको लागि बहु-संस्थागत क्षमता सुदृढीकरणमा केन्द्रित छ। यस स्तम्भमा देशमा सडक सुरक्षाका लागि संयुक्त राष्ट्रसंघीय महासन्धीहरूलाई अभ्यास गर्ने, सडक सुरक्षा गतिविधिहरूको नेतृत्व गर्ने बहु-पक्षीय राष्ट्रिय निकायको स्थापना गर्ने, राष्ट्रिय सडक सुरक्षा रणनीति विकास गर्ने र सम्बन्धित गतिविधिहरूको कार्यान्वयनको लागि यथार्थवादी र दीर्घकालीन लक्ष्यहरूको निर्धारण गर्नाका साथै तिनको कार्यान्वयनका लागि पर्याप्त रकमको सुनिश्चतता गर्ने कृयाकलापहरू पर्दछन्। यस स्तम्भले उपर्युक्त कृयाकलापहरूको प्रभावकारी रूपमा अनुगमन र मूल्याङ्कन गर्नको लागि तथ्यांक प्रणालीको विकासको लागि पनि आह्वान गर्दछ।

Questions and prompts

- तपाई कुन पदमा कार्यरत हुनुहुन्छ र तपाईंको मुख्य जिम्मेवारीहरु के के हुन्?
- तपाई यस पदमा कति समयदेखि हुनुहुन्छ ?
  - प्रम्प्ट: यस पदका लागि जिम्मेवारी कसरी निर्धारण भएका हुन्छन् ?
- स्तम्भ १ को वर्णनबाट तपाईं को विचारमा नेपालमा यस स्तम्भ अन्तर्गत गरिएका कृयाकलापहरूको अवस्था कस्तो छ ?
- तपाईको अनुभवबाट यस स्तम्भ अन्तरगतका कुन कुन कृयाकलापहरू राम्रोसँग अघि बढिरहेका छन् ?
  - प्रम्प्ट: तपाईंको विचारमा किन यो राम्रो हुँदैछ **?**
- तपाईको अनुभवमा यस स्तम्भ अन्तर्गतका कुन कामहरू राम्ररी भइरहेका छैन वा राम्रोसँग गर्न सकिएको छैन ?
  - प्रम्प्ट: तपाईंको विचारमा किन ती कृयाकलापहरू राम्रोसँग गर्न सकिएको छैन ?
- सडक सुरक्षाको लागि अपेक्षित उद्देश्यहरू प्राप्त गर्न तपाइँले के कस्ता चुनौतीहरू सामना गर्नु परेको छ ?
  - सडक सुरक्षाको नेतृत्व गर्ने संस्था वा निकायको स्थापनाबारे चुनौतिहरू?
  - राष्ट्रिय रणनीति बनाउने सम्बन्धी चुनौतिहरू?
  - तथ्यांक उत्पादन (Data generation) सम्बन्धी चुनौतिहरू?
  - अनुगमन र मूल्यांकन सम्बन्धी चुनौतिहरू?
- कस्ता जानकारी वा तथ्यहरू (Evidence) उपलब्ध भइदिएको भए तपाईंलाई यी उद्देश्यहरू प्राप्त गर्न मद्दत पुग्थ्यो जस्तो लाग्छ ?
- के तपाइँ स्तम्भ १ का यी गतिविधिहरूसँग सम्बन्धित अनुसन्धानमा कुनै कमी वा अन्तरहरू (Research Gaps) बारे सोचेर केही बताउन सक्नुहुन्छ ?
- हाम्रो यस कुराकानीको क्रममा केहि कुरा छुटेको पाउनु भए कृपया थप्नुहोस्?

For peer review only - http://bmjopen.bmj.com/site/about/guidelines.xhtml

At the end of the interview

- हामी अन्तर्वार्ताको अन्त्यमा पुग्यौं
- तपाईंको सहभागिताको लागि धन्यवाद
- तपाईंले दिनुभएको जानकारीले नेपालमा सडक सुरक्षाको स्थितिमा सुधार ल्याउन सम्भावित अनुसन्धानको लागि सुची बनाउन मद्दत गर्नेछ।
- हामी तपाईलाई यस अध्ययनको दोस्रो चरणको लागि पनि सम्पर्क गर्नेछौं जसमा हामी अन्तरवार्ताहरूबाट पहिचान गरेका सबै अनुसन्धान प्रश्नहरु प्रस्तुत गर्नेछौं र त्यसमध्ये कुन सबैभन्दा महत्वपुर्ण छ भनेर तपाईंको प्रतिक्रिया लिनेछौं ।
- अन्तिममा तपाईंको केहि प्रश्न छ कि?

स्तम्भ २. सुरक्षित सडक र गतिशिलता

(To be read to each participant for this pillar)
यो स्तम्भले सबै पैदलयात्री, साइकल चालक, मोटरसाइकल चालक लगायत सबै सडक प्रयोगकर्ताको फाइदाको लागि आवश्यक सुरक्षित सडक संजाल एवं पूर्वाधार सुधारलाई जोड दिन्छ । यस अन्तरगतका कृयाकलापहरूमा सडकको योजना, निर्माण तथा संचालनका चरणहरूमा नैं सडक सुरक्षालाई ध्यानमा राख्नु पर्ने; सडकको सुरक्षाको नियमित मुल्यांकन गरिनेछ भनेर सुनिस्चित गर्ने; र सम्बद्ध अधिकारीलाई सडक प्रयोगकर्ताको गतिशिलता (mobility) आवश्यकतालाई सम्बोधन गर्दा सबै किसिम र प्रकारका यातायात तथा पूर्वाधारलाई सुरक्षित पार्न प्रोत्साहन गर्ने जस्ता क्रियाकलापहरु पर्छन् ।

Suggested questions and prompts

- तपाई कुन पदमा कार्यरत हुनुहुन्छ र तपाईंको मुख्य जिम्मेवारीहरु के के हुन्?
- तपाई यस पदमा कति समयदेखि हुनुहुन्छ ?
  - प्रम्ट: यस पदका लागि जिम्मेवारी कसरी निर्धारण भएका हुन्छन् ?
- स्तम्भ २ को वर्णनबाट तपाईं को विचारमा नेपालमा यस स्तम्भ अन्तरगत गरिएका कृयाकलापहरूको अवस्था कस्तो छ ?
- तपाईको अनुभवबाट यस स्तम्भ अन्तरगतका कुन कुन कृयाकलापहरू राम्रोसँग अघि बढिरहेका छन् ?
  - प्रम्ट: तपाईंको विचारमा किन यो राम्रो हुँदैछ ?
- तपाईको अनुभवमा यस स्तम्भ अन्तरगतका कुन कामहरू राम्ररी भइरहेका छैन वा राम्रोसँग गर्न सकिएको छैन ?
  - प्रम्ट: तपाईंको विचारमा किन ती कृयाकलापहरू राम्रोसँग गर्न सकिएको छैन ?
- सडक सुरक्षाको लागि अपेक्षित उद्देश्यहरू प्राप्त गर्न तपाईँले के कस्ता चुनौतीहरू सामना गर्दै हुनुहुन्छ ?
    - सडक सुरक्षाको स्वामित्व र उत्तरदायित्व प्रवर्धनसम्बन्धी चुनौती?
    - सबै सडक प्रयोगकर्ताहरूको आवश्यकतालाई सम्बोधन गर्नेबारे चुनौतीहरू?
    - सडक डिजाइन, निर्माण वा मर्मतसँग सम्बन्धी चुनौतिहरू?
- कस्ता जानकारी वा तथ्यहरू (Evidence) उपलब्ध भइदिएको भए तपाईंलाई यी उद्देश्यहरू प्राप्त गर्न मद्दत पुग्थ्यो जस्तो लाग्छ ?
- के तपाइँ स्तम्भ २ का यी गतिविधिहरुसंग सम्बन्धित अनुसन्धानमा कुनै कमी वा अन्तरहरू (Gaps) बारे सोचेर केही बताउन सक्नुहुन्छ ?

For peer review only - http://bmjopen.bmj.com/site/about/guidelines.xhtml

- हाम्रो यस कुराकानीको क्रममा केहि कुरा छुटेको पाउनु भए कृपया थप्नुहोस?

At the end of the interview

- हामी अन्तर्वार्ताको अन्त्यमा पुग्यौं
- तपाईंको सहभागिताको लागि धन्यवाद
- तपाईंले दिनुभएको जानकारीले नेपालमा सडक सुरक्षाको स्थितिमा सुधार ल्याउन सम्भावित अनुसन्धानको लागि सुची बनाउन मद्दत गर्नेछ।
- हामी तपाईलाई यस अध्ययनको दोस्रो चरणको लागि पनि सम्पर्क गर्नेछौं जसमा हामी अन्तरवार्ताहरूबाट पहिचान गरेका सबै अनुसन्धान प्रश्नहरु प्रस्तुत गर्नेछौं र त्यसमध्ये कुन सबैभन्दा महत्वपुर्ण छ भनेर तपाईंको प्रतिक्रिया लिनेछौं ।
- अन्तिममा तपाईंको केहि प्रश्न छ कि?

स्तम्भ ३ सुरक्षित वाहनहरू

(To be read to each participant for this pillar)
कमसल मापदण्ड भएका वाहनहरूले उल्लेखनीय संख्यामा सडक दुर्घटना र हताहती गराइरहेका हुन्छन्। यस स्तम्भले सुरक्षा प्रबर्धन गर्न उत्तम अभ्यास, वाहन सुरक्षाका मानकहरू र प्रविधिको प्रयोग गर्न प्रोत्साहन गर्दछ। यस अन्तरगतका गतिविधिहरूमा नयाँ कारको सुरक्षा मुल्यांकन कार्यक्रम (NCAP सुरक्षा रेटिंगहरू) को कार्यान्वयन गर्ने, विद्यमान सवारी साधनहरूमा न्यूनतम सुरक्षा सुविधाहरू भएको सुनिश्चत गर्न वाहन सुरक्षा जाँचहरू समावेश गर्ने, जस्तै दुर्घटनामा परेका व्यक्तिमा दुर्घटनाको प्रभाव कम गर्न सीट बेल्ट जोडिएको र बत्ती एवम् ब्रेक ठीक अवस्थामा छ भन्ने सुनिश्चत गर्दछ ।

Suggested questions and prompts

- तपाई कुन पदमा कार्यरत हुनुहुन्छ र तपाईंको मुख्य जिम्मेवारीहरु के के हुन्?
- तपाई यस पदमा कति समयदेखि हुनुहुन्छ ?
    - प्रम्ट: यस पदका लागि जिम्मेवारी कसरी निर्धारण भएका हुन्छन् ?
- स्तम्भ ३ को वर्णनबाट तपाईं को विचारमा नेपालमा यस स्तम्भ अन्तरगत गरिएका कृयाकलापहरूको अवस्था कस्तो छ ?
- तपाईको अनुभवबाट यस स्तम्भ अन्तरगतका कुन कुन कृयाकलापहरू राम्रोसँग अघि बढिरहेका छन् ?
    - प्रम्ट: तपाईंको विचारमा किन यो राम्रो हुँदैछ **?**
- तपाईको अनुभवमा यस स्तम्भ अन्तरगतका कुन कामहरू राम्ररी भइरहेका छैन वा राम्रोसँग गर्न सकिएको छैन ?
    - तपाईंको विचारमा किन ती कृयाकलापहरू राम्रोसँग गर्न सकिएको छैन ?
    - NCAP सुरक्षा रेटिंगहरू (अथवा नयाँ कारको सुरक्षा मुल्यांकन कार्यक्रम) सम्बन्धमा तपाइँको के विचार छ?
    - के हामीसँग सवारी साधन सम्बन्धी राम्रा कानूनहरू छन् जसले बढी सुरक्षित गाडीहरूको आयात बढाउने वा विद्यमान सवारी साधनहरूमा सुधार वा मर्मत गरी तिनलाइ सुरक्षित पार्न मद्दत गर्दछन्?
- सडक सुरक्षाको लागि अपेक्षित उद्देश्यहरू प्राप्त गर्न तपाइँले के कस्ता चुनौतीहरू सामना गर्दै हुनुहुन्छ ?
    - राष्ट्रिय कानूनहरूलाई अन्तर्राष्ट्रिय मोटर वाहन सुरक्षा नियमहरुसँग सामंजस्यता गर्दाका चुनौतिहरु?

For peer review only - http://bmjopen.bmj.com/site/about/guidelines.xhtml

- जोखिममा रहेका सडक प्रयोगकर्ताहरूका जोखिम कम गर्न डिजाइन गरिएको सुरक्षा प्रविधिहरूका बारे अनुसन्धानसम्बन्धी चुनौतिहरुरू
- कस्ता जानकारी वा तथ्यहरू (Evidence) उपलव्ध भइदिएको भए तपाईंलाई यी उद्देश्यहरू प्राप्त गर्न मद्दत पुग्थ्यो जस्तो लाग्छ ?
- के तपाइँ स्तम्भ ३ का यी गतिविधिहरुसंग सम्बन्धित अनुसन्धानमा कुनै कमी वा अन्तरहरू (Gaps) बारे सोचेर केही बताउन सक्नुहुन्छ ?
- हाम्रो यस कुराकानीको क्रममा केहि कुरा छुटेको पाउनु भए कृपया थप्नुहोस?

At the end of the interview

- हामी अन्तर्वार्ताको अन्त्यमा पुग्यौं
- तपाईंको सहभागिताको लागि धन्यवाद
- तपाईंले दिनुभएको जानकारीले नेपालमा सडक सुरक्षाको स्थितिमा सुधार ल्याउन सम्भावित अनुसन्धानको लागि सुची बनाउन मद्दत गर्नेछ।
- हामी तपाईलाई यस अध्ययनको दोस्रो चरणको लागि पनि सम्पर्क गर्नेछाैं जसमा हामी अन्तरवार्ताहरूबाट पहिचान गरेका सबै अनुसन्धान प्रश्नहरु प्रस्तुत गर्नेछाैं र त्यसमध्ये कुन सबैभन्दा महत्वपुर्ण छ भनेर तपाईंको प्रतिक्रिया लिनेछौं ।
- अन्तिममा तपाईंको केहि प्रश्न छ कि?

स्तम्भ ४ सुरक्षित सडक प्रयोगकर्ताहरू

(To be read to each participant for this pillar)
स्तम्भ ४ सबै सडक प्रयोगकर्ताहरूको व्यवहार सुधार गर्न व्यापक कार्यक्रमहरू विकासमा केन्द्रित छ। यस अन्तरगत उदाहरणीय सडक सुरक्षा कानून र मापदण्ड अपनाउने र तिनको निरन्तर वा बढ्दो क्रममा पालनामा गराउने गतिविधिहरू समावेश छन। यस्ता प्रयासहरूमा जनचेतना र मानिसहरुलाई सुरक्षित राख्ने उपायहरू जस्तैः सीट बेल्ट र हेलमेट लगाउने बानी प्रवर्द्धन गर्न र तीव्र गतिमा वाहन चलाउने, रक्सी वा मादकपदार्थ सेवन गरी गाडी चलाउने र यस्तै अन्य जोखिमपूर्ण व्यवहारमा कमी ल्याउन व्यवहारिक शिक्षा दिने कृयाकलापहरू पनि समावेश छन्। यस स्तम्भले कामसँग सम्बन्धित (पेशागत) सडक दुर्घटनाहरू कम गर्ने गतिविधिहरूको लागि आह्वान गर्दछ र भरखरै चालक अनुमतिपत्र (लाइसेन्स) लिएका नयाँ चालकहरूको लागि उनीहरूको सवारी चालक अनुमतिपत्र (लाइसेन्स) लाई क्रमैसँग स्तरोन्नति गर्ने (graduated driver licensing) कार्यक्रमहरूलाई बढावा दिन अह्वान गरेको छ।

<u>Suggested questions and prompts</u>

- तपाई कुन पदमा कार्यरत हुनुहुन्छ र तपाईंको मुख्य जिम्मेवारीहरु के के हुन्?
- तपाई यस पदमा कति समयदेखि हुनुहुन्छ ?
  - प्रम्प्टः यस पदका लागि जिम्मेवारी कसरी निर्धारण भएका हुन्छन् ?
- स्तम्भ ४ को वर्णनबाट तपाईं को विचारमा नेपालमा यस स्तम्भ अन्तरगत गरिएका कृयाकलापहरूको अवस्था कस्तो छ ?
- तपाईको अनुभवबाट यस स्तम्भ अन्तरगतका कुन कुन कृयाकलापहरू राम्रोसँग अघि बढिरहेका छन् ?
  - <u>प्रम्प्ट</u>: तपाईंको विचारमा किन यो राम्रो हुँदैछ **?**

For peer review only - http://bmjopen.bmj.com/site/about/guidelines.xhtml

- तपाईंको अनुभवमा यस स्तम्भ अन्तरगतका कुन कामहरू राम्ररी भइरहेका छैन वा राम्रोसँग गर्न सकिएको छैन ?
    - तपाईंको विचारमा किन ती कृयाकलापहरू राम्रोसँग गर्न सकिएको छैन ?
- सडक सुरक्षाको लागि अपेक्षित उद्देश्यहरू प्राप्त गर्न तपाइँले के कस्ता चुनौतीहरू सामना गर्दै हुनुहुन्छ ?
        - कानूनको पालना र कार्यान्वयनको स्थिति कस्तो छ?
        - सडक सुरक्षा कानूनको पालनालाई सुदृढ पार्न के गर्न सकिन्छ?
        - सुरक्षित चालक / यात्री व्यवहारको अवलम्बन गर्ने चलन कस्तो छ (उदाहरणको लागि सीटबेल्ट / हेलमेट प्रयोग, मा.प.से.)
        - सडक सुरक्षाको लागि चालक अनुमतिपत्र (लाइसेन्स) के भूमिका खेल्छ?
        - सडक प्रयोगकर्ताका व्यवहारसँग सम्बन्धित कुनैं कमीकमजोरीहरू हामीलाई थाहा छन् ? तिनलाई कसरी परिवर्तन गर्न सकिन्छ ?
        - सडक सुरक्षा सम्बन्धी कानूनी प्रावधानमा के कस्ता कमी कमजोरी छन् ? तिनको पालनालाई कसरी सडक प्रयोगकर्ताहरूको व्यवहारसँग जोडिएकोछ ?
- कुन नयाँ जानकारी वा तथ्य उपलब्ध भइदिए सडक प्रयोगकर्ताहरूको सुरक्षित व्यवहार अवलम्बनलाई सुधार गर्न मद्दत पुग्नेछ?
- के तपाइँ स्तम्भ ४ का यी गतिविधिहरुसंग सम्बन्धित अनुसन्धानमा कुनै कमी वा अन्तरहरू (Gaps) बारे सोचेर केही बताउन सक्नुहुन्छ ?
- हाम्रो यस कुराकानीको क्रममा केहि कुरा छुटेको पाउनु भए कृपया थप्नुहोस?

At the end of the interview
- हामी अन्तर्वार्ताको अन्त्यमा पुग्यौं
- तपाईंको सहभागिताको लागि धन्यवाद
- तपाईंले दिनुभएको जानकारीले नेपालमा सडक सुरक्षाको स्थितिमा सुधार ल्याउन सम्भावित अनुसन्धानको लागि सुची बनाउन मद्दत गर्नेछ।
- हामी तपाईलाई यस अध्ययनको दोस्रो चरणको लागि पनि सम्पर्क गर्नेछौं जसमा हामी अन्तरवार्ताहरूबाट पहिचान गरेका सबै अनुसन्धान प्रश्नहरु प्रस्तुत गर्नेछौं र त्यसमध्ये कुन सबैभन्दा महत्वपुर्ण छ भनेर तपाईंको प्रतिक्रिया लिनेछौं ।
- अन्तिममा तपाईंको केहि प्रश्न छ कि?

स्तम्भ ५. दुर्घटनापश्चातको स्याहार

(To be read to each participant for this pillar)

स्तम्भ ५ ले दुर्घटना पछिको इमरजेन्सीकोलागि प्रतिक्रियामा सुधारको साथै दुर्घटनाका घाइतेलाई उपयुक्त इमरजेन्सी उपचार सेवा एवम् दीर्घकालीन पुनर्स्थापना सेवा दिने स्वास्थ्य र अन्य प्रणालीको क्षमताको सुधारको आवश्यकतालाई सम्बोधन गर्दछ। घाइतेलाई अस्पताल लैजानु अघि गर्नु पर्ने स्याहार प्रणाली, अस्पताल ट्रामा स्याहार प्रणाली, पीडितलाई पुनर्स्थापनाका साथै दीर्घकालीन चिकित्सा सहायता प्रणालीको विकास एवम् सुधार र इमरजेन्सी अवस्थामा सहयोग लिन प्रयोग गरिने एउटै नम्बरको बिकास र सुधारका कृयाकलापहरू दुर्घटना पश्चातको स्याहारका मुख्य बुँदाहरू हुन् ।

Suggested questions and prompts
- तपाई कुन पदमा कार्यरत हुनुहुन्छ र तपाईंको मुख्य जिम्मेवारीहरु के के हुन्?
- तपाई यस पदमा कति समयदेखि हुनुहुन्छ ?

For peer review only - http://bmjopen.bmj.com/site/about/guidelines.xhtml

  - प्रम्प्टः यस पदका लागि जिम्मेवारी कसरी निर्धारण भएका हुन्छन् ?
- स्तम्भ १ को वर्णनबाट तपाईं को विचारमा नेपालमा यस स्तम्भ अन्तरगत गरिएका कृयाकलापहरूको अवस्था कस्तो छ ?
- तपाईको अनुभवबाट यस स्तम्भ अन्तरगतका कुन कुन कृयाकलापहरू राम्रोसँग अघि बढिरहेका छन् ?
  - प्रम्प्ट: तपाईंको विचारमा किन यो राम्रो हुँदैछ ?
- तपाईको अनुभवमा यस स्तम्भ अन्तरगतका कुन कामहरू राम्ररी भइरहेका छैन वा राम्रोसँग गर्न सकिएको छैन ?
  - तपाईंको विचारमा किन ती कृयाकलापहरू राम्रोसँग गर्न सकिएको छैन ?
- सडक सुरक्षाको लागि अपेक्षित उद्देश्यहरू प्राप्त गर्न तपाइँले के कस्ता चुनौतीहरू सामना गर्नु परेको छ ?
    - किन देशभरी नै आपत्कालिन सेवा जस्तै एम्बुलेन्स सेवाको लागी एउटै टेलिफोन नम्बर छैन ?
    - तपाईं नेपालका अस्पतालको ट्रमा सेवाहरूलाई कसरी हेर्नुहुन्छ ?
- कुन नयाँ जानकारी वा तथ्य उपलब्ध भइदिए सडक प्रयोगकर्ताहरूको सुरक्षित व्यवहार अवलम्बनलाई सुधार गर्न मद्दत पुग्नेछ?
- के तपाइँ स्तम्भ ५ का यी गतिविधिहरुसंग सम्बन्धित अनुसन्धानमा कुनै कमी वा अन्तरहरू (Gaps) बारे सोचेर केही बताउन सक्नुहुन्छ ?
- हाम्रो यस कुराकानीको क्रममा केहि कुरा छुटेको पाउनु भए कृपया थप्नुहोस?

At the end of the interview

- हामी अन्तर्वार्ताको अन्त्यमा पुग्यौं
- तपाईंको सहभागिताको लागि धन्यवाद
- तपाईंले दिनुभएको जानकारीले नेपालमा सडक सुरक्षाको स्थितिमा सुधार ल्याउन सम्भावित अनुसन्धानको लागि सुची बनाउन मद्दत गर्नेछ।
- हामी तपाईलाई यस अध्ययनको दोस्रो चरणको लागि पनि सम्पर्क गर्नेछौं जसमा हामी अन्तरवार्ताहरूबाट पहिचान गरेका सबै अनुसन्धान प्रश्नहरु प्रस्तुत गर्नेछौं र त्यसमध्ये कुन सबैभन्दा महत्वपुर्ण छ भनेर तपाईंको प्रतिक्रिया लिनेछौं ।
- अन्तिममा तपाईंको केहि प्रश्न छ कि?

For peer review only - http://bmjopen.bmj.com/site/about/guidelines.xhtml

CREDES checklist adapted from Saskia et al 2017. *Guidance on Conducting and REporting DElphi Studies (CREDES) in palliative care: Recommendations based on a methodological systematic review*. Palliative Medicine. available from https://journals.sagepub.com/doi/10.1177/0269216317690685

Box 3. Recommendations for the Conducting and REporting of DElphi Studies (CREDES).

Rationale for the choice of the Delphi technique

1. *Justification.* The choice of the Delphi technique as a method of systematically collating expert consultation and building consensus needs to be well justified. When selecting the method to answer a particular research question, it is important to keep in mind its constructivist nature

Planning and design

2. *Planning and process.* The Delphi technique is a flexible method and can be adjusted to the respective research aims and purposes. Any modifications should be justified by a rationale and be applied systematically and rigorously
3. *Definition of consensus.* Unless not reasonable due to the explorative nature of the study, an a priori criterion for consensus should be defined. This includes a clear and transparent guide for action on (a) how to proceed with certain items or topics in the next survey round, (b) the required threshold to terminate the Delphi process and (c) procedures to be followed when consensus is (not) reached after one or more iterations

Study conduct

4. *Informational input.* All material provided to the expert panel at the outset of the project and throughout the Delphi process should be carefully reviewed and piloted in advance in order to examine the effect on experts' judgements and to prevent bias
5. *Prevention of bias.* Researchers need to take measures to avoid directly or indirectly influencing the experts' judgements. If one or more members of the research team have a conflict of interest, entrusting an independent researcher with the main coordination of the Delphi study is advisable
6. *Interpretation and processing of results.* Consensus does not necessarily imply the 'correct' answer or judgement; (non)consensus and stable disagreement provide informative insights and highlight differences in perspectives concerning the topic in question
7. *External validation.* It is recommended to have the final draft of the resulting guidance on best practice in palliative care reviewed and approved by an external board or authority before publication and dissemination

Reporting

8. *Purpose and rationale.* The purpose of the study should be clearly defined and demonstrate the appropriateness of the use of the Delphi technique as a method to achieve the research aim. A rationale for the choice of the Delphi technique as the most suitable method needs to be provided
9. *Expert panel.* Criteria for the selection of experts and transparent information on recruitment of the expert panel, socio-demographic details including information on expertise regarding the topic in question, (non)response and response rates over the ongoing iterations should be reported
10. *Description of the methods.* The methods employed need to be comprehensible; this includes information on preparatory steps (How was available evidence on the topic in question synthesised?), piloting of material and survey instruments, design of the survey instrument(s), the number and design of survey rounds, methods of data analysis, processing and synthesis of experts' responses to inform the subsequent survey round and methodological decisions taken by the research team throughout the process
11. *Procedure.* Flow chart to illustrate the stages of the Delphi process, including a preparatory phase, the actual 'Delphi rounds', interim steps of data processing and analysis, and concluding steps
12. *Definition and attainment of consensus.* It needs to be comprehensible to the reader how consensus was achieved throughout the process, including strategies to deal with non-consensus
13. *Results.* Reporting of results for each round separately is highly advisable in order to make the evolving of consensus over the rounds transparent. This includes figures showing the average group response, changes between rounds, as well as any modifications of the survey instrument such as deletion, addition or modification of survey items based on previous rounds
14. *Discussion of limitations.* Reporting should include a critical reflection of potential limitations and their impact of the resulting guidance
15. *Adequacy of conclusions.* The conclusions should adequately reflect the outcomes of the Delphi study with a view to the scope and applicability of the resulting practice guidance
16. *Publication and dissemination.* The resulting guidance on good practice in palliative care should be clearly identifiable from the publication, including recommendations for transfer into practice and implementation. If the publication does not allow for a detailed presentation of either the resulting practice guidance or the methodological features of the applied Delphi technique, or both, reference to a more detailed presentation elsewhere should be made (e.g. availability of the full guideline from the authors or online; publication of a separate paper reporting on methodological details and particularities of the process (e.g. persistent disagreement and controversy on certain issues)). A dissemination plan should include endorsement of the guidance by professional associations and health care authorities to facilitate implementation

**Item#**	**Description**	**Section/Page # reported in the manuscript**
Rationale for the choice of the Delphi technique		
1	Justification.	Methods, first paragraph, page 4
Planning and design		
2	Planning and process	METHODS, pages 4-6

3	Definition of Consensus	Data collection and analysis, page 5, paragraph 2
Study conduct		
4	Informational input	Recruitment of participants, page 4
5	Prevention of bias	Strengths and limitations, page 14-15
6	Interpretation and processing results	Data collection, paragraphs 2-4, pages 5-6
7	External validations	Overarching consensus workshop, page 6.
Reporting		
8	Purpose and Rationale	INTRODUCTION, page 3
9	Expert panel	Description of participants, Results, pages 6-7 and Table 1
10	Description of the methods	METHODS, pages 4-6
11	Procedure	METHODS, pages 4-6 Flow chart, Figure 1, page 19
12	Definition and attainment of consensus	Paragraphs 2 and 3 of Data collection and analysis, pages 5-6 + overall consensus workshop paragraph, page 6
13	Results	Results pages 6-11, Table 3 (page 9-11) and Table 4 (page 11)
14	Discussion of limitations	Strengths and limitations, page 14
15	Adequacy of conclusions	CONCLUSIONS, page 15
16	Publication and dissemination	Not applicable as this is not a Delphi study supporting guidelines for clinical practice.

For peer review only

BMJ Open

Identifying research priorities for road safety in Nepal: a Delphi study

Journal:	*BMJ Open*
Manuscript ID	bmjopen-2021-059312.R2
Article Type:	Original research
Date Submitted by the Author:	23-Mar-2022
Complete List of Authors:	Pant, Puspa Raj; University of the West of England, Faculty of Health and Applied Sciences<br>Rana, Pranita; Kathmandu Medical College, Nepal Injury Research Centre<br>Pradhan, Kriti; Kathmandu Medical College, Nepal Injury Research Centre<br>Joshi, Sunil Kumar; Kathmandu Medical College and Teaching Hospital, Community Medicine<br>Mytton, Julie; University of the West of England, Faculty of Health and Applied Sciences
**Primary Subject Heading**:	Health policy
Secondary Subject Heading:	Public health
Keywords:	Health policy < HEALTH SERVICES ADMINISTRATION & MANAGEMENT, PUBLIC HEALTH, ACCIDENT & EMERGENCY MEDICINE

SCHOLARONE™
Manuscripts

I, the Submitting Author has the right to grant and does grant on behalf of all authors of the Work (as defined in the below author licence), an exclusive licence and/or a non-exclusive licence for contributions from authors who are: i) UK Crown employees; ii) where BMJ has agreed a CC-BY licence shall apply, and/or iii) in accordance with the terms applicable for US Federal Government officers or employees acting as part of their official duties; on a worldwide, perpetual, irrevocable, royalty-free basis to BMJ Publishing Group Ltd ("BMJ") its licensees and where the relevant Journal is co-owned by BMJ to the co-owners of the Journal, to publish the Work in this journal and any other BMJ products and to exploit all rights, as set out in our licence.

The Submitting Author accepts and understands that any supply made under these terms is made by BMJ to the Submitting Author unless you are acting as an employee on behalf of your employer or a postgraduate student of an affiliated institution which is paying any applicable article publishing charge ("APC") for Open Access articles. Where the Submitting Author wishes to make the Work available on an Open Access basis (and intends to pay the relevant APC), the terms of reuse of such Open Access shall be governed by a Creative Commons licence – details of these licences and which Creative Commons licence will apply to this Work are set out in our licence referred to above.

Other than as permitted in any relevant BMJ Author's Self Archiving Policies, I confirm this Work has not been accepted for publication elsewhere, is not being considered for publication elsewhere and does not duplicate material already published. I confirm all authors consent to publication of this Work and authorise the granting of this licence.

For peer review only

Identifying research priorities for road safety in Nepal: a Delphi study

Corresponding author: Dr. Puspa Raj Pant[a*]

[a]Faculty of Health and Applied Sciences, University of the West of England, Bristol, BS16 1QY, United Kingdom, Email: pant.puspa@gmail.com ORCiD: https://orcid.org/0000-0002-8827-0018

Co-authors:

Ms. Pranita Rana[b], [b]Nepal Injury Research Centre, Kathmandu Medical College Public Ltd, Kathmandu, Nepal.

Ms. Kriti Pradhan[b], [b]Nepal Injury Research Centre, Kathmandu Medical College Public Ltd, Kathmandu, Nepal.

Prof Sunil Kumar Joshi[c], [c]Department of Community Medicine, Kathmandu Medical College Public Ltd, Kathmandu, Nepal. ORCiD: https://orcid.org/0000-0002-2704-5060

Prof Julie Mytton[a], [a]Faculty of Health and Applied Sciences, University of the West of England, Bristol, UK. ORCiD: https://orcid.org/0000-0002-0306-4750

Word count: 4,211 (excluding title page, abstract, references, figures, and tables.)

For peer review only - http://bmjopen.bmj.com/site/about/guidelines.xhtml

For peer review only

Abstract: (291 words)

Objective: To identify and prioritise the research needed to help Nepali agencies develop an improved road safety system.

Design: Delphi study.

Setting: Nepal.

Participants: Stakeholders from government institutions, academia, engineering, healthcare and civil society were interviewed to identify knowledge gaps and research questions. Participants then completed two rounds of ranking and a workshop.

Results: A total of 93 participants took part in interviews and two rounds of ranking. Participants were grouped with others sharing expertise relating to each of the five World Health Organization 'pillars' of road safety: 1) road safety management; 2) safer roads; 3) safer vehicles; 4) safer road users; and 5) effective post-crash response. Interviews yielded 1019 research suggestions across the five pillars. Two rounds of ranking within expert groups yielded consensus on the important questions for each pillar. A workshop involving all participants then led to the selection of 6 questions considered the most urgent: (1) How can implementing agencies be made more accountable? (2) How should different types of roads, and roads in different geographical locations, be designed to make them safer for all road users? (3) What vehicle fitness factors lead to road traffic crashes? (4) How can the driver licensing system be improved to ensure safer drivers? (5) What factors lead to public vehicle crashes and how can they be addressed? (6) What factors affect emergency response services getting to the patient and then getting them to the right hospital in the best possible time?

Conclusions: The application of the Delphi approach is useful to enable participants representing a range of institutions and expertise to contribute to the identification of road safety research priorities. Outcomes from this study provide Nepali researchers with a greater understanding of the necessary focus for future road safety research.

Keywords: Safer Road Users, Road Traffic Injuries, Road Safety Pillars, Post-crash Response, Delphi.

Strengths and limitations of this study

For peer review only - http://bmjopen.bmj.com/site/about/guidelines.xhtml

- 93 Nepali experts (70% of 133 approached) participated, bringing perspectives from road construction, vehicle management, transport management, and post-crash response.
- Most participants had a remit for national road safety, however, 83/93 (89%) were from organisations based in Kathmandu valley, which may have risked a focus on urban and highway crashes.
- The research questions identified were ranked by the participants individually as well as discussed during group meetings to achieve consensus.
- We were able to retain a high proportion of participants through the study: 64/93 participants took part in Round 3 (69% retention).

For peer review only

For peer review only - http://bmjopen.bmj.com/site/about/guidelines.xhtml

INTRODUCTION

Globally road traffic injuries are increasing, with an estimated 1.35 million deaths and up to 50 million non-fatal injuries in 2016. [1] Despite having only 1% of the world's vehicles, low-income countries have 13% of fatal road traffic injuries. Road traffic injuries are the leading cause of death for children and young adults between 5-29 years globally and are an important cause of disability and poverty. RTIs have been estimated to generate losses of up to 6.5% of a low-income country's gross domestic product. [2]

The World Health Organization (WHO) World Report on Road Traffic Injury Prevention, [3] subsequent Road Safety Status Reports [1] and the WHO Save LIVES technical package of 22 evidence-based interventions [4] argue for a "safe systems approach" [5] to reduce road dangers and the numbers of people killed and seriously injured on the roads. This approach recognises the essential contribution of different sectors to create a system that keeps road users safe. The World Health Organization published the Global Plan of Action for Road Safety 2011-2020 [6] alongside the United Nations and this plan of action recommended five 'pillars'; road safety management (Pillar 1), safer roads and mobility (Pillar 2), safer vehicles (Pillar 3), safer road users (Pillar 4) and post-crash response (Pillar 5). Action across all five pillars can contribute to reduced road traffic injuries. Nepal has been a co-sponsor of these principles, but progress has been limited.

A large road construction programme in Nepal has seen over 15,000 km of new blacktop, gravel, and earthen roads built by federal, provincial, and local governments in the last 5 years [7] and there are plans to have a total of 13,500 km blacktopped road by 2023/24. [8] Many new roads do not have proven safety features and are poorly maintained. The roads in the hills are considered to be dangerous because of landslides in addition to frequent road crashes due to poor engineering or poor safety infrastructure. [9] The Department of Transport Management in the Government of Nepal produces vehicle registration statistics that show more than half (53%) of the 3.22 million motorised vehicles in Nepal were registered between July 2013 and July 2018 and about 78% of total registered vehicles were motorcycles. [10]

Nepal lacks a funded road safety implementation plan, a national ambulance service, or globally recognised vehicle standards. The national helmet-wearing law is not enforced for motorcycle passengers and there is no legislation for passenger seatbelt use, child restraints, or mobile phone use whilst driving. Data are limited and of poor quality; WHO estimates of road traffic fatalities in Nepal in 2016 (4,622) are more than double those recorded by the Traffic Police (2,006), and there are no routinely published estimates of deaths by road user category available. [1] Nepal's Health

For peer review only - http://bmjopen.bmj.com/site/about/guidelines.xhtml

Management Information System recorded over 100,000 hospital visits for the treatment of orthopaedic problems secondary to road traffic events in the year 2017/18 indicating the significant burden of road traffic injuries on health systems. [11] Road traffic crashes and injuries in Nepal are rising despite existing legislation. [12 13] Tackling road traffic injuries was a priority in the government's Health Sector Strategy 2015-2020. [14] A National Road Safety Action Plan 2013-2020 [15] was acknowledged but not ratified by Parliament. Neither document specified the research required to support the delivery of improved road safety.

To improve road safety, coordinated efforts are needed across the road transport system. Research is vital to optimise decision-making. Current initiatives in Nepal for the control and prevention of road traffic crashes and their consequences are not based on local evidence. Therefore, this study aimed to involve a wide range of experts and participants representing stakeholder organisations to identify the research needed to help agencies in Nepal develop a safe systems approach to road safety, and achieve a consensus about which studies should be prioritised.

METHODS

This study used the Delphi approach [16-18] to develop a consensus on a prioritised list of road safety research questions. Five groups of stakeholders in Nepal were engaged. The roles and experience of participants were relevant to each of the five WHO pillars of road safety. The study was conducted in two stages: firstly, interviews were conducted with stakeholders to identify a range of possible research questions, and secondly, participants completed two rounds of ranking the research questions in order of importance. Each of the five road safety pillars was studied separately. Five interview topic guides were developed in the Nepali language, based on the activities recommended for each of the five WHO pillars of road safety (Supplementary file 1).

Participant recruitment

Potential study participants were identified through existing networks and multi-sector stakeholder groups on road safety and first response convened by the Nepal Injury Research Centre. Networks included third sector and advocacy organisations for road safety. Participants helped identify further potential participants through a snowballing approach where they advised the research team of individuals who may be appropriate to invite to take part. We aimed to recruit 20-25 participants for each of the five pillars. Potential participants were contacted by telephone and were provided with information about the study and their interest in our research was confirmed. For participants expressing an interest, written information regarding the study and a consent form were sent to the

For peer review only - http://bmjopen.bmj.com/site/about/guidelines.xhtml

potential participants via email. All the recruitment took place during the novel coronavirus disease (COVID-19) pandemic and therefore most of the interviews were completed remotely, by phone or video call. For these participants, consent was recorded verbally at the start of the interview or was collected before participation via email. Later in the pandemic, it became feasible to engage some participants face-to-face. For these participants consent was collected at this meeting.

Data collection and analysis

In Round 1, we conducted interviews with participants in which we asked what additional data or information would help them in their job and reduce road traffic injuries. We explored the barriers they faced when tackling road safety. Most of the interviews were conducted using online platforms such as MS Teams, Zoom, Google Meet, or Viber, and some interviews were conducted over the telephone. Towards the end of the data collection period, and when COVID-19 pandemic restrictions allowed, we conducted a small number of face-to-face interviews where this was the preference of the participants. In these circumstances, mitigations against infection, such as social distancing and the wearing of face masks, helped protect both participants and researchers. Interviews were conducted in the Nepali language and audio-recorded. Audio recordings were listened to several times. Information relating to perceived gaps in research or evidence was documented as potential research questions on a spreadsheet, in English. For each group of stakeholders, approximately 200 research suggestions were generated from the interviews. Many of the participants raised similar issues, therefore it was possible to cluster the questions into groups, and to formulate a single question to represent that area of research need. The grouping stage was completed collaboratively by the whole research team to ensure that questions were treated equally and the process consistently applied. A reduced list of about 30 questions was achieved, identifying the research and evidence needs relating to each pillar of road safety.

For Round 2, the research questions from the reduced list were uploaded to an online survey tool (Qualtrics) in both English and Nepali languages. The link to the survey was distributed to the participants via email or Viber message. Participants were asked to give their opinion on the importance of each research question using a 5-point Likert scale: *Not Important*, *Slightly Important*, *Moderately Important*, *Important*, and *Very Important*. Reminders to complete the survey were sent via email and individual phone calls after one week and followed up again 2-3 days later. Completed surveys were exported from Qualtrics and analysed in MS Excel. Survey results were collated to identify the number of participants who rated each question as "important" or "very important". Questions where a significant majority of participants had scored them 'important' or 'very

For peer review only - http://bmjopen.bmj.com/site/about/guidelines.xhtml

important' were retained as prioritised questions. For Pillars 1, 3, 4, and 5 we retained questions where ≥70% of the participants rated the questions as "important" or "very important". For Pillar 2 we retained questions where ≥80% of participants rated at these levels, since a greater proportion of the questions were considered important. We used these threshold values based on published Delphi studies. [19 20]

For Round 3, participants were invited to a real-time online workshop where the prioritised questions were presented and discussed. The workshop was designed to allow the participants to share their views and listen to each other's opinions regarding which issues were the most important to research. These workshops were recorded and shared with those who were not able to join. Following the workshop, a Qualtrics survey was sent to all participants again, this time listing only those questions prioritised from Round 2. Participants were again asked to score each question as either *Not Important*, *Slightly Important*, *Moderately Important*, *Important*, or *Very Important*. Reminders were sent to the participants after one week and followed up again after 2-3 days. Completed surveys were exported to MS Excel and collated to identify the number of participants considering each question 'important' or 'very important'. This resulted in the final prioritised list of research questions for each pillar of road safety.

The research team completed Rounds 1, 2 and 3 for one pillar before moving on to the next pillar. The interviews started on 12 July 2020 and were completed on 14 February 2021. Due to the COVID-19 pandemic, where government officials and clinical staff were not easily available to participate, stakeholders in Pillars 1 and 5 were left until later in the study when the peak of the first wave of COVID-19 in Nepal had passed.

Overarching consensus workshop

A final online consensus workshop was organised where the top-ranked research questions from all five pillars were shared with all the participants, stakeholders from our advisory groups, and invited key decision-makers. A facilitated discussion explored the understanding of what the different research options could provide and how that new evidence could potentially be used. Using online voting software (Mentimeter, https://www.menti.com), participants were encouraged to vote for one research question from each pillar that they considered needed to be addressed the most urgently. The questions considered most urgent were presented back to the group.

For peer review only - http://bmjopen.bmj.com/site/about/guidelines.xhtml

Ethical approval for conducting this study was obtained from the Kathmandu Medical College Institutional Review Committee (ref. 040620201) and the University of the West of England Bristol Faculty Research Ethics Committee (ref. HAS. 20.06.192).

Patient and public involvement

Through community engagement and involvement, we engaged individuals with diverse views on road safety, ranging from road users to those with decision-making authority for road development, management, and traffic regulation.

RESULTS

Study participants

Out of a total of 133 potential participants identified and contacted, 93 individuals were recruited and took part in interviews covering all five road safety pillars. Two participants had expertise relevant to more than one pillar, and therefore took part in two interviews; one for each pillar. Participants were from a range of organisational and professional backgrounds, including government institutions, academia, road safety engineers, clinicians, civil society organisations, and all had an interest or remit that addressed one or more of the five pillars of road safety. Some of the experts in our list, when contacted, suggested the name of other stakeholders. Out of 93 participants, 83 were from Kathmandu valley and represented organisations with the remit to work or influence road safety nationally. Ten participants were from outside Kathmandu and added value to the study by providing local contexts. The participants' background characteristics are summarised in Table 1.

Table 1. Organisational/professional background of the participants

Organisational / professional background	Total	Male	Female
Government organisation (Secretaries, Govt Officers, Police, Political representatives)	33	30	3
Clinician, Nurse, physiotherapist	10	8	2
Road Safety Engineer	9	9	0
Road Safety Advocacy	8	5	3
Academics	7	6	1
First Aid/ emergency/ ambulance provider	6	6	0

For peer review only

Engineers' Association	4	4	0
Transport worker	4	4	0
Automobile dealer	3	3	0
Federation of transport	2	2	0
Schools' organisation	2	2	0
Sustainable Transport	2	2	0
Others (journalist and city planners)	3	3	0
**Total**	**93**	**84**	**9**

Across all five pillars, we identified a total of 1019 research suggestions from the 95 interviews completed in Round 1. Collating similar questions reduced this to 141 questions across the five pillars. Seventy-six (80%) participants took part in Round 2, through which the list of questions was reduced to 91 questions. Forty (43%) participants took part in an online workshop before further ranking in Round 3 which was completed by 64 (69%) participants and resulted in a total of 30 prioritised questions. Figure 1 shows the stages of the Delphi study and the number of participants in each round. Attrition of participants was greatest for the group discussing Pillar 1 (road safety management), where 10/21 (48% participants) dropped out between Round 1 and Round 3. Attrition was least in the group discussing Pillar 2 (safer roads) where only 3/18 (17%) of participants were lost.

Figure 1. Flowchart of the Delphi process

The high attrition of participants in Pillar 1 was not unexpected since many of these participants worked in government positions and it was difficult for them to prioritise attendance during the COVID-19 pandemic. Figure 2 illustrates participant attrition throughout the study.

Figure 2. Study participants retained in subsequent Delphi rounds, by pillar

Table 2 describes the number of research questions prioritised in each Round, split by the pillars of road safety. The retention rate in this study was equivalent to that in other published Delphi studies [21] despite the COVID-19 pandemic.

Table 2. Research questions prioritised at each Round, by pillar

**Pillar of road safety**	**Round 1**	**Round 2**	**Round 3**

For peer review only

For peer review only - http://bmjopen.bmj.com/site/about/guidelines.xhtml

	Interview dates	Number of interviews (online or by phone)	Research questions generated ('long list')	Grouped research questions ('reduced list')	Number of 'Important' or 'very important' research questions (above 70% consensus)	Number of questions considered most important (Top 5 ranks)
Pillar 1 (road safety management)	23 Nov to 22 Jan 2021	21 (21)	183	25	17	5
Pillar 2 (safer roads and mobility)	13 July to 12 Aug 2020	18 (4)	211	30	19*	5
Pillar 3 (safer vehicles)	16 Aug to 15 Sep 2020	17 (17)	217	30	20	7
Pillar 4 (safer road users)	23 Sept to 19 Oct 2020	20 (20)	178	30	20	6
Pillar 5 (post-crash response)	05 Jan to 14 Feb 2021	19 (13)	230	26	15	7
Total		95 (75)	1019	141	91	30

*80% consensus

The top-ranked research questions for the five pillars of road safety are presented in Table 3. The research questions that were considered the most important cover a wide range of issues, including how to make existing processes more effective, how to assess the training needs of the road safety workforce, understanding the challenges of implementing existing road safety legislation, how to improve accountability for road safety, how to generate and disseminate better information to inform decisions, and how to generate evidence that supports the economic argument for road safety.

For peer review only - http://bmjopen.bmj.com/site/about/guidelines.xhtml

1 **Table 3. List of top questions for Pillars 1 to 5 with scores in Rounds 2 and 3**

	**Scores***	
**Pillar 1: Road safety management**	**R2**	**R3**
How can implementing agencies be made more accountable for road safety in urban and rural areas?	92	91
What are the barriers to conducting road safety audits at all stages of road construction and implementation of their recommendations?	85	91
How can urban and rural roads construction and management be governed to ensure improved road safety?	92	91
How can the traffic management system be improved to ensure it improves the safety of all road users?	85	91
What are the barriers to the implementation of existing laws related to road safety in Nepal?	69	91
**Pillar 2: Safer roads**		
What is the effectiveness of different safety features installed on roads in terms of crash reduction?	94	100
What are the barriers and facilitators for achieving safer roads in Nepal?	88	100
What kind of institutional setup is needed at central, provincial, and local levels for the promotion of road safety ownership and accountability?	94	93
What are the economic benefits of the installation of safety features during road construction, regular maintenance, and upgrading of roads?	82	93
How should different types of roads, and roads in different geographical locations, be designed to make them safer for all road users?	82	93
**Pillar 3: Safer vehicles**		
What are the factors affecting fitness condition and roadworthiness of vehicles to the extent that it leads to road traffic crashes?	86	100
What should be the minimum criteria for the establishment of standard vehicular maintenance workshops?	93	92
What are the capacity development and training needs for currently working human resources and additional jobs to improve the safety of vehicles in Nepal?	71	92
What improvements in policies and institutional setup are needed to ensure vehicle safety of all types and routes?	79	92
What is the role of motor parts used for vehicle maintenance for fitness condition of the vehicles and road crashes?	93	83

For peer review only - http://bmjopen.bmj.com/site/about/guidelines.xhtml

For peer review only

How does overloading impact the safety of the vehicles?	71	83
What are the vehicle-related factors causing road crashes in Nepal?	71	83
**Pillar 4: Safer road users**		
How can the driver licensing system be made more effective to ensure safer vehicle drivers?	100	93
What are the main factors increasing the risk of public vehicle crashes? What interventions would improve the safety of travel on public vehicles?	94	93
How can licensing and crash data collection systems be improved?	94	93
What are the major causes of road crashes in Nepal? What percentage of road crashes are due to unsafe road user behaviours?	94	87
What content should be included in awareness campaigns for different types of road users, and how are these campaigns best delivered?	83	87
What are the barriers to the implementation of laws regarding safer road user behaviour? Review of existing policies related to safer road users.	78	87
**Pillar 5: Post-crash response**		
What standards should be applied to ambulance services? (includes standards for personnel and training, equipment carried, and the vehicles)	100	100
What is the standard of care at health centres and hospitals for road traffic injury patients across the country, and how can they be improved?	79	92
What is the current average time taken for a road traffic injury patient to receive first response at the scene and the average time taken to arrive at a healthcare setting able to meet their care needs? How can any delays be reduced?	93	92
What factors influence the ability of the post-crash emergency response service to get to the patient and then get them to the right hospital in the best possible time?	86	92
What should be included in the training curriculum for the different levels of post-crash responders?	93	85
How should policies and legislation be further developed to support the post-crash response for road traffic injury victims?	71	85
What is the optimal model of insurance to minimise death & disability following a road traffic crash? What are the barriers & facilitators to implementing such an insurance system?	71	85

*Percent of participants ranked "very important" or "important"; R2= Round 2; R3= Round 3.

Note: The phrasing of questions presented in this table reflects the direct translation from Nepali to English of the research questions used in the ranking process.

For peer review only - http://bmjopen.bmj.com/site/about/guidelines.xhtml

A total of 56 people (47 participants and 9 key decision-makers) attended the workshop conducted at the end of the study where the list of the top-ranked research questions for each of the five pillars were presented. Using electronic voting software to identify the question within each Pillar considered to be the most urgent, 6 questions were prioritised. Two questions in Pillar 4 were scored equally (Table 4).

Table 4. Top 6 most urgent research questions

Pillars	Research Questions
Pillar 1	How can implementing agencies be made more accountable for road safety in urban and rural areas?
Pillar 2	How should different types of roads, and roads in different geographical locations, be designed to make them safer for all road users?
Pillar 3	What are the factors affecting fitness condition and road worthiness of vehicles to the extent that it leads to road traffic crashes?
Pillar 4	How can the driver licensing system be made more effective to ensure safer vehicle drivers?
	What are the main factors increasing the risk of public vehicle crashes? What interventions would improve the safety of travel on public vehicles?
Pillar 5	What factors influence the ability of the post-crash emergency response service to get to the patient and then get them to the right hospital in the best possible time?

DISCUSSION

This study is the first to our knowledge that has engaged such a wide group of participants to identify the research priorities relevant to the improvement of road safety in Nepal. The research team identified and invited 133 potential participants to join the study, and 70% (n=93) agreed to take part. Respondents included stakeholders from a range of organisational and professional backgrounds as well as geographical areas and included; officials in government institutions (Ministerial Secretaries, Government Officers, Police, Political representatives), clinicians, nurses, physiotherapists, engineers, academics, first responders, transport workers, automobile dealers,

For peer review only - http://bmjopen.bmj.com/site/about/guidelines.xhtml

road users, members of the media and city planners. The proportion of women working in roles related to road safety in Nepal is low, and we were pleased to have been able to recruit 9/93 (10%) female participants, which is in line with official data on the Nepali workforce. The number of participants that should take part in a Delphi study is not prescribed and it can be anywhere above 10 persons; the number is guided by the scope of the problem and existing resources. [19 20] Overall, the retention of the participants until the third round of ranking was excellent however, rates varied between different pillars. The overall retention rate of 69% and 50% attendance at the final consensus workshop indicated the high level of interest in road safety research in Nepal. This response rate is higher than that reported by Marchau and Van der Heijden [22] in a multi-country road safety study. Marchau and Van der Heijden [22] applied the Delphi technique to explore the policy aspects of implementing driver support systems. The authors used a questionnaire with specified answer options sent to international experts from the USA, Japan, and Europe. In this study, 56% (65 out of 117) of invitees responded in the first round while only 40 responded in the third round.

Road safety research is a neglected issue in low- and middle-income countries [23] and a lack of research capacity may be one reason for the limited progress to date. In Nepal, a policy review identified that institutional arrangements and resource allocation for road safety were inadequate. [24] The lack of coordination of road safety sectors is a challenge globally [25 26] as well as in Nepal. Many of the participants in this study had the opportunity to meet and discuss road safety with those working in other sectors, for the first time.

Other studies exploring aspects of road safety through the use of the Delphi technique have mostly come from high-income countries, except a few, such as Vietnam and Uganda. Studies have explored specific risk factors such as cell phone use and sleep deprivation in the USA, [27 28] and public bus safety in Italy. [29] Some studies focussed on the need to improve post-crash care such as; strengthening trauma management in Vietnam, [30] pre-hospital emergency care in Iran, [31] post-recovery rehabilitation in Australia, [32] and emergency medical services capacity in Uganda. [33] In Iran, Delphi studies have been conducted to inform the development of minimum datasets to study road crashes, [34] and developing a national road safety education programme. [35] We have not identified any previously published Delphi studies that have included all five pillars of road safety in a single study.

Zhu, et al. [27] recruited road safety experts and young drivers in the USA to study the risks of mobile phone use while driving. Expert participants identified texting, sending emails, or picking up the phone as particularly high-risk behaviours for crashes, but not playing music on a handheld mobile which was prioritised by young drivers. Participants identified 20 behavioural practices related to

For peer review only - http://bmjopen.bmj.com/site/about/guidelines.xhtml

mobile phone use which can result in a collision. Our study participants in Pillar 4 also identified the importance of studying causes of driver distraction but did not identify mobile phone use in particular.

Cafiso, et al. [29] engaged the managers of large public bus companies in Italy in a Delphi study to explore bus safety. Participants rated safety solutions for issues relating to driver behaviour, traffic conflicts, and vehicle maintenance and technology. Our study participants also raised concerns about the safety of public transport users and the safety of public passenger vehicles and prioritised a study to investigate the factors contributing to public vehicle crashes. The technological solutions explored in the study by Cafisco (e.g. technology to control when the bus can start, automatic door closing, etc.) are not applicable in the context of Nepal where public passenger vehicles are older and poorly equipped. An expert panel on sleep deprivation in a study by Czeisler, et al. [28], agreed that a driver was not fit to drive if they had less than 2 hours of sleep in the previous 24 hours. In our study participants raised concerns regarding driver behaviour, including fatigue but prioritised a study to review the entire driver licensing system rather than focussing on tackling specific driver behaviours. These examples illustrate how previous Delphi studies have tended to focus on specific road safety issues, and how the results are specific to the context or participants. Neither of these studies would be directly generalizable to Nepal, nor do they cover the breadth of safety issues identified in our study.

Several Delphi studies have reported post-crash trauma management and prehospital care. In Vietnam, Schmucker, et al. [30] used online meetings followed by a questionnaire survey of 1000 road users to generate responses that were ranked, and outcomes were used to inform the development of a trauma care course. Our study participants for Pillar 5 also prioritised the development of training curricula for different levels of post-crash trauma care (table 3). Recently, Azami-Aghdash, et al. [31] used the Delphi technique to achieve a consensus on 37 indicators to measure and improve the performance of prehospital care following road crashes in Iran. This is similar to the topic prioritised for post-crash response (Pillar 5) in our study. However, the differences in Iranian and Nepali country contexts and pre-hospital care infrastructure mean that performance indicators in Iran are not generalizable to Nepal. Balikuddembe, et al. [33] used the Delphi technique to identify and prioritise factors that could prevent and support victims of road traffic injuries in Kampala. They identified 23 factors across the entire Emergency Medical Service system that were similar to issues raised by participants in Pillar 5 of our study.

For peer review only - http://bmjopen.bmj.com/site/about/guidelines.xhtml

In the course of our study, shifts in the opinions of participants were observed during Rounds 2 and 3. Concerning the rankings completed in Round 2, a high degree of consensus was observed and the process of creating a reduced list for Round 3 was relatively straightforward. The Delphi method dictates that the results of a first-round be re-presented to participants in subsequent Rounds, giving participants the opportunity to reconsider their views in the light of the discussion, additional thought, and/or the results obtained from other participants. [20 36] Cafiso, et al. [29] in their study, similarly reported that after the second Round, the Delphi panelists' opinions were influenced by those of their colleagues. In our study, the changed ranks of the questions between Round 2 and Round 3 illustrate the value and influence of discussion between Rounds in reaching a consensus. High numbers of research questions were rated 'important' or 'very important' in our study, illustrating that many participants recognised the need for road safety research in Nepal. Issues relating to improving the safety of road users traditionally considered vulnerable (e.g. pedestrians, cyclists, drivers, and passengers of powered two-wheelers) were raised by participants in this study, however, during ranking, research questions that improved the safety of all road users were prioritised over questions relating to these specific groups.

The Government of Nepal plans to enact a Road Safety Bill [37] that will include issues relating to planning, resourcing, implementation, and evaluation of national road safety activities. Provincial Governments, which were established only 4 years ago, through the promulgation of the constitution of Nepal, [38] have started to enact Provincial Transport Management Acts. However, the institutional structures necessary to implement these laws are still in development. [24] The research questions prioritised in this study emphasise the need for evidence to support both national development plans [8] and safer roads and transport in Nepal. [39] Existing road safety policies are mostly only partially implemented. [24] Policy gaps include policies to separate traffic and road users and those to address speed management.

Strengths and limitations

The high response rate (70%), and good representation and involvement of individuals and experts currently active in the fields of road construction, vehicle management, transport management, and post-crash response is a major strength of this study. The Delphi method for achieving consensus is a research technique with the potential for biases; [20] Hallowell [17] outlined common biases in implementation and here we describe the measures applied to minimise these biases in this study. To minimize factors that might influence the quality of the conclusions due to the level of expertise of the panel members, [40] only experienced and recognised authorities working for road safety in

For peer review only - http://bmjopen.bmj.com/site/about/guidelines.xhtml

Nepal were invited to participate. While most participants had a remit for national road safety, we acknowledge that 83/93 (89%) were from organisations based in Kathmandu valley which may have introduced a bias towards urban and highway crashes in the prioritised research questions. The results produced by Delphi studies may be considered limited due to the poor quality of the facilitator's survey instruments, [16] therefore, the tools developed for this study were informed by the international literature and advice was available from an experienced Delphi expert. Bias can occur if questions are poorly worded [17] therefore our researchers were trained in interviewing skills before commencing Round 1 and conducted the interview in Nepali. Some critics believe that convergence of opinion in Delphi studies is conformity. [18] To counter this risk, we synthesised best global road safety practice as reported in published literature and presented this to participants during the workshops between Rounds 2 and 3. This meant that participants ranked questions initially individually and then were allowed to change their minds after the group discussion. Although the Delphi approach has been reported to be time-intensive, [41] we found that the time taken to participate in this study did not significantly affect recruitment or retention. . We successfully retained participants, as demonstrated by the fact that 64/93 (69%) participants were retained to Round 3.

CONCLUSIONS

This study identified research priorities for road safety in Nepal across all of the WHO's five pillars of road safety. The most urgent and important research questions related to: improving the governance of road safety through greater accountability, improving road design across different topographies, establishing the contribution of poor vehicle fitness to crash occurrence, strengthening the driver licensing system, improving the safety of passengers on public buses, and understanding the barriers to the provision of effective post-crash care. These findings can guide researchers when designing future studies. In addition, the study provided opportunities for participants to meet stakeholders outside their sector and discuss the challenges identified. Future research has the potential to lead to evidence-informed policy development and implementation, and improve practices relating to road construction and management, vehicle standards, and post-crash care, making the roads safer for all road users in Nepal.

Acknowledgments: We would like to acknowledge the support of Professor Nichola Rumsey who provided training in Delphi study methodology to the study team, advised on the study protocol and

For peer review only - http://bmjopen.bmj.com/site/about/guidelines.xhtml

this manuscript. We are grateful to all 93 expert participants without whom this study would not have been possible.

Contributors: Puspa Raj Pant: data curation; project administration; original draft. Pranita Rana: data collection; investigation; project administration; validation; review & editing. Kriti Pradhan: data collection; investigation; project administration; validation; review & editing. Sunil Kumar Joshi: project administration; resources; supervision; review & editing. Julie Mytton: conceptualization; data curation; funding acquisition; methodology; supervision; review & editing.

Funding: This research was commissioned by the National Institute for Health Research (NIHR) Global Health Policy and Systems Research Development Award using UK aid from the UK Government (NIHR129877). The views expressed in this publication are those of the author(s) and not necessarily those of the NIHR or the Department of Health and Social Care.

Competing interest: The authors have no competing interests to declare.

Data availability statement: Data are available upon reasonable request.

References:

1. World Health Organization. Global status report on road safety 2018: World Health Organization 2018.
2. World Bank. Guide for Road Safety Opportunities and Challenges: Low- and Middle-Income Countries Country Profiles. . Washington DC, USA: World Bank, 2019.
3. Peden M, Scurfield R, Sleet D, et al. World report on road traffic injury prevention: World Health Organization Geneva, 2004.
4. WHO. Save LIVES - A road safety technical package. Geneva: World Health Organization, 2017.
5. World Road Association. The Safe System Approach. In: Kienreich R, Milton J, eds. Road Safety Manual: A manual for practitioners and decision makers on implementing safe system infrastructure: World Road Association (PIARC), 2019.
6. WHO. Global plan for the decade of action for road safety 2011-2020. Geneva: World Health Organization, 2011.
7. Ministry of Finance. Economic Survey 2020/21. Ministry of Finance, Kathmandu, 2021.

For peer review only - http://bmjopen.bmj.com/site/about/guidelines.xhtml

8. National Planning Commission. The fifteenth plan (FY 2019/20 to 2023/24) unofficial English translation. Kathmandu: Government of Nepal, 2020.

9. McAdoo BG, Quak M, Gnyawali KR, et al. Roads and landslides in Nepal: how development affects environmental risk. *Natural Hazards and Earth System Sciences* 2018;18(12):3203-10.

10. Department of Transport Management. Statistical details of vehicles registered in Nepal up to FY 2017/18 [Online]. Kathmandu2020 [Statstics of registered vehicles in Nepal]. Available from: https://www.dotm.gov.np/Files/NoticePDF/vehiclesdatatill2074-752020-01-17_04-49-13-732.pdf accessed 24 May 2021.

11. Ministry of Health and Population. HMIS Database 2074/75 by Local Government Kathmandu: Department of Health Services; 2021 [updated 25 March 2020. Available from: https://dohs.gov.np/ihims-raw-data/ accessed 1 April 2021.

12. Ojha KN. Road safety status and some initiatives in Nepal. *ITEGAM-JETIA* 2021;7(27):20-40.

13. Pant PR, Mytton J, Dharel MR, et al. The prevention of–and first response to–injuries in Nepal: a review of policies and legislation. *Health research policy and systems* 2021;19(1):1-20.

14. Ministry of Health and Population. Nepal Health Sector Strategy 2015-2020 Kathmandu: Ministry of Health and Population, Government of Nepal, 2015.

15. Goverment of Nepal. Nepal Road Safety Action Plan (2013 - 2020): Draft. Kathmandu, Nepal: Department of Road, Goverment of Nepal, 2011.

16. Gupta UG, Clarke RE. Theory and applications of the Delphi technique: A bibliography (1975–1994). *Technological Forecasting and Social Change* 1996;53(2):185-211. doi: https://doi.org/10.1016/S0040-1625(96)00094-7

17. Techniques to minimize bias when using the Delphi method to quantify construction safety and health risks. Construction Research Congress 2009: Building a Sustainable Future; 2009.

18. Rowe G, Wright G. The Delphi technique: Past, present, and future prospects—Introduction to the special issue. *Technological forecasting and social change* 2011;78(9):1487-90.

19. Hasson F, Keeney S, McKenna H. Research guidelines for the Delphi survey technique. *Journal of advanced nursing* 2000;32(4):1008-15.

20. Powell C. The Delphi technique: myths and realities. *Journal of Advanced Nursing* 2003;41(4):376-82. doi: https://doi.org/10.1046/j.1365-2648.2003.02537.x

21. Hamlet C, Rumsey N, Williamson H, et al. Consensus research priorities for facial palsy: A Delphi survey of patients, carers, clinicians and researchers. *Journal of Plastic, Reconstructive & Aesthetic Surgery* 2018;71(12):1777-84.

For peer review only - http://bmjopen.bmj.com/site/about/guidelines.xhtml

22. Marchau V, Van der Heijden R. Policy aspects of driver support systems implementation: results of an international Delphi study. *Transport Policy* 1998;5(4):249-58.

23. Perel P, Ker K, Ivers R, et al. Road safety in low-and middle-income countries: a neglected research area. *Injury Prevention* 2007;13(4):227-27.

24. Pant PR, Mytton J, Dharel MR, et al. The prevention of – and first response to – injuries in Nepal: a review of policies and legislation. *Health Research Policy and Systems* 2021;19(1):65. doi: 10.1186/s12961-021-00686-1

25. Bliss T, Breen J. Meeting the management challenges of the Decade of Action for Road Safety. *IATSS research* 2012;35(2):48-55.

26. Torbaghan ME, Sasidharan M, Burrow M, et al. Lessons from road safety research and policy engagement in Pakistan, Nepal, Tanzania. 2019

27. Zhu M, Rudisill TM, Rauscher KJ, et al. Risk perceptions of cellphone use while driving: Results from a delphi survey. *International journal of environmental research and public health* 2018;15(6):1074.

28. Czeisler CA, Wickwire EM, Barger LK, et al. Sleep-deprived motor vehicle operators are unfit to drive: a multidisciplinary expert consensus statement on drowsy driving. *Sleep health* 2016;2(2):94-99.

29. Cafiso S, Di Graziano A, Pappalardo G. Using the Delphi method to evaluate opinions of public transport managers on bus safety. *Safety science* 2013;57:254-63.

30. Schmucker U, Ottersbach C, Frank M, et al. A new approach and first steps to strengthen trauma management and road safety in North Vietnam. *Journal of trauma management & outcomes* 2008;2(1):1-7.

31. Azami-Aghdash S, Moosavi A, Gharaee H, et al. Development of quality indicators to measure pre-hospital emergency medical services for road traffic injury. *BMC health services research* 2021;21(1):1-12.

32. Smits EJ, Gane EM, Brakenridge CL, et al. Expert consensus and perspectives on recovery following road traffic crashes: a Delphi study. *Disability and rehabilitation* 2020:1-10.

33. Balikuddembe JK, Ardalan A, Khorasani-Zavareh D, et al. Factors affecting the exposure, vulnerability and emergency medical service capacity for victims of road traffic incidents in Kampala Metropolitan Area: a Delphi study. *BMC emergency medicine* 2016;17(1):1-8.

34. Mohammadi A, Ahmadi M, Gharagozlu A. Developing a minimum data set for an information management system to study traffic accidents in Iran. *Iranian Red Crescent Medical Journal* 2016;18(3)

For peer review only

35. Bakhtari-Aghdam F, Sadeghi-Bazargani H, Azami-Aghdash S, et al. Developing a national road traffic safety education program in Iran. *BMC public health* 2020;20(1):1-13.
36. Williamson K. The Delphi Method. Research methods for students, academics and professionals: Information management and systems. Second ed. New South Wales: Kirsty Williamson 2002.
37. RSS Nepal. Call to pass bill related to road safety. *The Himalayan Times* 2019 3 Feb 2019.
38. Government of Nepal. The Constitution of Nepal. Kathmandu, 2015.
39. Government of Nepal. Vehicle and Transportation Management Act 1993 (Ammendment 2019). Kathmandu, 1993.
40. Veltri AT. Expected use of management principles for safety function management. West Virginia University, 1985.
41. Rajendran S. Sustainable construction safety and health rating system. 2006

Figure Titles

Figure 1. Flowchart of the Delphi process

Figure 2. Study participants retained in subsequent Delphi rounds, by pillar

For peer review only

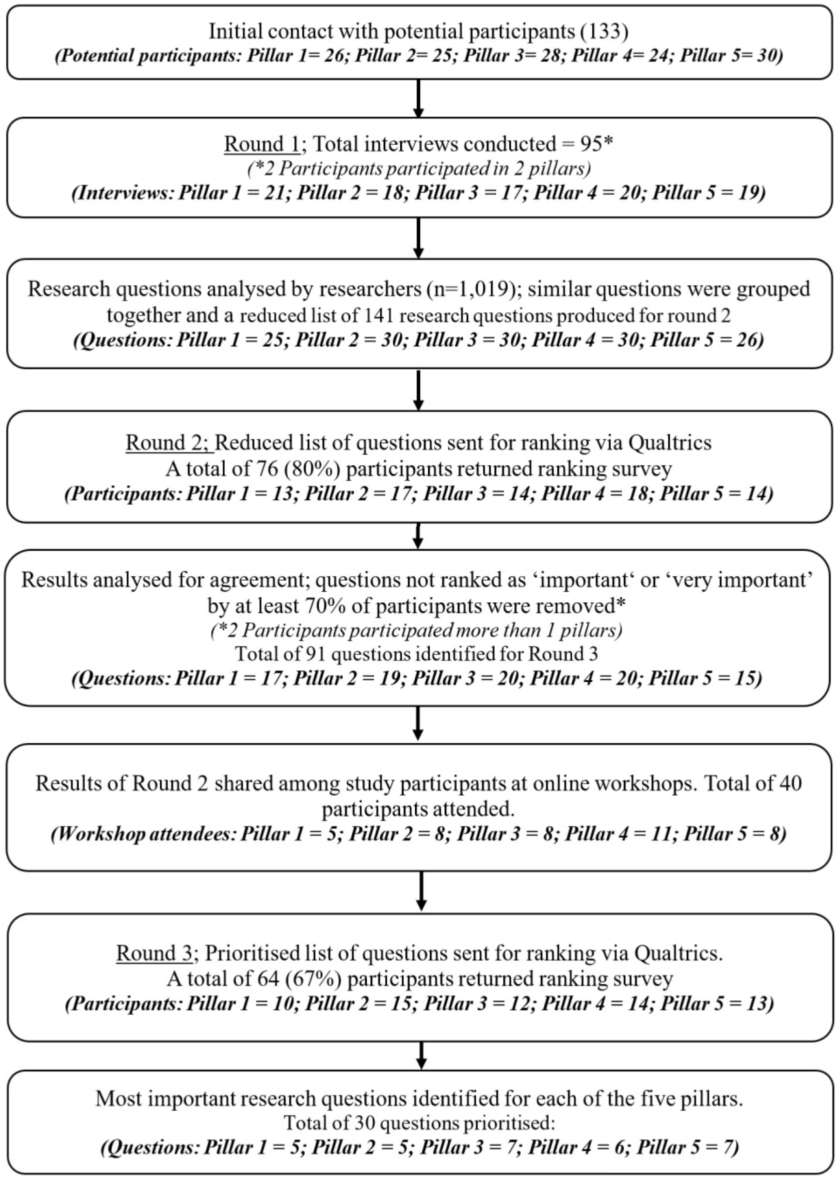

Figure 1. Flow chart of the Delphi process

376x535mm (96 x 96 DPI)

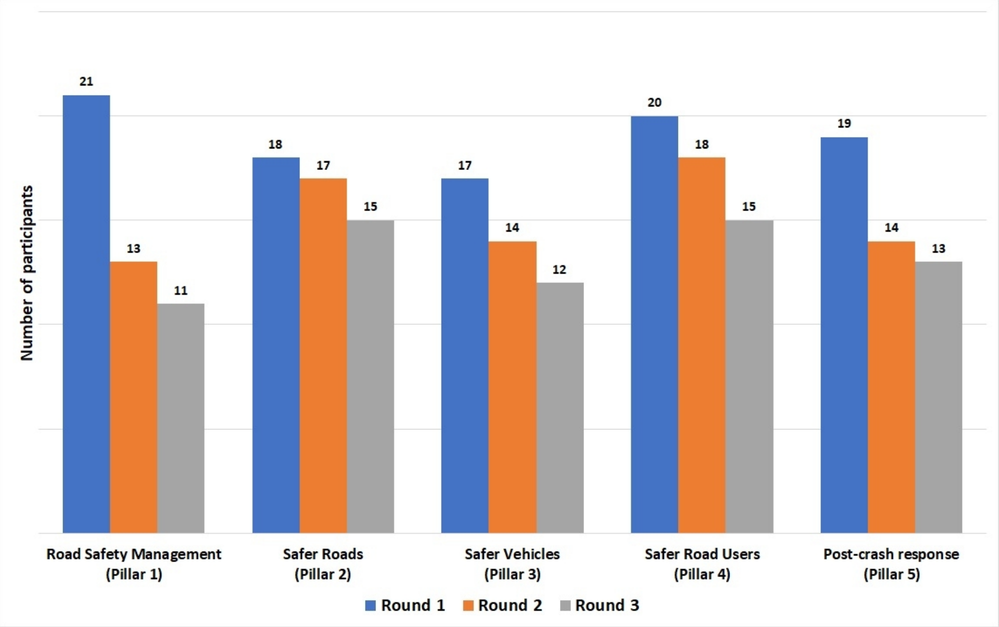

Figure 2. Study participants retained in subsequent Delphi rounds, by pillar.

302x189mm (96 x 96 DPI)

Interview guide

Road Safety Research Prioritisation study

Pillar 1: Road safety management

(To be read to each participant for this pillar) *This pillar focuses on strengthening multi-agency capacity for road safety. It includes activities such as putting into practice major UN road safety conventions, establishing a multi-sectoral national agency to lead road safety activities, developing a national road safety strategy and setting realistic and long-term targets for related activities with sufficient funding for implementation. It also calls for the development of data systems to effectively monitor and evaluate activities.*

Questions and prompts

- What is your job title and what is the focus of your responsibilities for this position?
- How long have you been in this role?
    - Prompt: How these responsibilities are determined?
- From the description of Pillar 1, what is the status of activities for this pillar in Nepal?
- From your experience, what is going well?
    - *Prompt: Why do you think it is going well?*
- From you experience what has not yet happened or is not working well?
    - *Prompt: Why do you think it is not working well?*
- What challenges are you facing to achieve your desired objectives for road safety?
    - *Prompts:*
        - *challenges regarding to have a lead agency?*
        - *challenges regarding national strategy?*
        - *challenges regarding data generation?*
        - *challenges regarding monitoring and evaluation?*
- What information or evidence do you think would help you achieve these objectives?
- Can you think of any gaps in the research available to you, related to these activities in pillar 1?
- Would you like to add anything which we have not covered during this conversation?

At the end of the interview

- Explain that this is the end of the interview.
- Thank the participant for their time.
- Explain that the information they have given will be used to create a list of possible research ideas to improve road safety in Nepal.
- Explain that they will be invited to the next stage of the study where they will hear all of the research questions we have identified, and they will be invited to tell us which ones they think are the most important.
- Ask if they have any questions before you go.

For peer review only - http://bmjopen.bmj.com/site/about/guidelines.xhtml

Pillar 2. Safer roads and mobility

(To be read to each participant for this pillar) *This pillar highlights the need to improve the safety of road networks and infrastructure for the benefit of all road users, including the pedestrians, bicyclists and motorcyclists. Activities include considering safety during the planning, design, construction and operation of roads; making sure that roads are regularly assessed for safety; and encouraging the relevant authorities to consider all forms of transport and types of safe infrastructure when they respond to the mobility needs of road users.*

Suggested questions and prompts

- What is your job title and what is the focus of your responsibilities for this position?
- How long have you been in this role?
  - Prompt: How these responsibilities are determined?
- From the description of Pillar 2, what is the status of activities for this pillar in Nepal?
- From your experience, what is going well?
  - *Prompt: Why do you think it is going well?*
- From you experience what has not yet happened or is not working well?
  - *Prompt: Why do you think it is not working well?*
- What challenges you are facing to achieve your desired objectives for road safety?
  - *Prompts*
    - *challenges to promote road safety ownership and accountability?*
    - *challenges promoting (addressing) the needs of all road users?*
    - *challenges relating to designing, building or maintaining roads?*
- What information or evidence do you think would help you achieve these objectives?
- Can you think of any gaps in the research available to you, related to these activities?
- Would you like to add anything which we have not covered during this conversation?

At the end of the interview

- Explain that this is the end of the interview.
- Thank the participant for their time.
- Explain that the information they have given will be used to create a list of possible research ideas to improve road safety in Nepal.
- Explain that they will be invited to the next stage of the study where they will hear all of the research questions we have identified and they will be invited to tell us which ones they think are the most important.
- Ask if they have any questions before you go.

Pillar 3. Safer vehicles

(To be read to each participant for this pillar) *Poor vehicle standards contribute to a significant number of crashes and casualties. This pillar encourages use of best practice vehicle safety standards and technology to promote safety. Activities may include implementing new car assessment programmes (such as NCAP safety ratings) and vehicle safety checks on existing vehicles to ensure they are equipped with minimum safety features, such as seat-belts to minimise the impact of crashes to occupants, and working lights and brakes.*

Suggested questions and prompts

- What is your job title and what is the focus of your responsibilities for this position?

For peer review only - http://bmjopen.bmj.com/site/about/guidelines.xhtml

- How long have you been in this role?
  - Prompt: How these responsibilities are determined?
- From the description of Pillar 3, what is the status of activities for this pillar in Nepal?
- From your experience, what is going well?
  - *Prompt: Why do you think it is going well?*
- From you experience what has not happened or is not working well?
  - *Prompts*
    - *Why do you think it is not working well?*
    - *What are your views on the New Car Assessment Programme (NCAP)?*
    - *do we have good vehicle-related laws that could promote the import of safer vehicles or the maintenance of existing vehicles?*
- What challenges you are facing to achieve desired objectives for road safety?
    - *challenges to harmonise international motor vehicle safety regulations with national laws?*
    - *research about safety technologies designed to reduce risk to vulnerable road users.*
- What information or evidence do you think would help you achieve these objectives?
- Can you think of any gaps in the research available to you, related to these activities?
- Would you like to add something else which we have not covered during this conversation?

At the end of the interview

- Explain that this is the end of the interview.
- Thank the participant for their time.
- Explain that the information they have given will be used to create a list of possible research ideas to improve road safety in Nepal.
- Explain that they will be invited to the next stage of the study where they will hear all of the research questions we have identified and they will be invited to tell us which ones they think are the most important.
- Ask if they have any questions before you go.

Pillar 4. Safer road users

(To be read to each participant for this pillar) *Pillar 4 focuses on developing comprehensive programmes to improve the behaviour of all road users. Activities include the adoption of model road safety legislation and sustained or increased enforcement or road safety laws and standards. These efforts are combined with public awareness and education to increase uptake of behaviours that keep people safe (e.g. seat-belt and helmet wearing) and to reduce behaviours that cause harm (e.g. speeding, taking alcohol or drugs when driving) and other risks. It also calls for activities to reduce work-related road traffic injuries and promoted the establishment of graduated driver licensing programmes for novice drivers.*

Suggested questions and prompts

- What is your job title and what is the focus of your responsibilities for this position?
- How long have you been in this role?
  - Prompt: How these responsibilities are determined?
- From the description of Pillar 4, what is the status of activities for this pillar in Nepal?
- From your experience, what is going well?

For peer review only - http://bmjopen.bmj.com/site/about/guidelines.xhtml

    - *Prompt: Why do you think it is going well?*
- From you experience what has not happened or is not working well?
    - *Prompt: Why do you think it is not working well?*
- What challenges you are facing to achieve desired objectives for road safety?
    - *Prompts*
        - *what is the status of law enforcement?*
        - *what could be done to strengthen road safety law enforcement?*
        - *How good is the uptake of safe driver / passenger behaviours (e.g. seatbelt / helmet use)?*
        - *What role do driving licences play in road safety?*
        - *Are there any gaps in what we know about road user behaviours and how to change them?*
        - *what about gaps in legislation or how it is enforced relating to road users behaviour?*
- What new information or evidence do you think would help you to improve the uptake of safe road user behaviours?
- Can you think of any gaps in the research available to you, related to these activities in Pillar 4?
- Would you like to add something else which we have not covered during this conversation?

At the end of the interview

- Explain that this is the end of the interview.
- Thank the participant for their time.
- Explain that the information they have given will be used to create a list of possible research ideas to improve road safety in Nepal.
- Explain that they will be invited to the next stage of the study where they will hear all of the research questions we have identified and they will be invited to tell us which ones they think are the most important.
- Ask if they have any questions before you go.

Pillar 5. Post-crash response

(To be read to each participant for this pillar) *Pillar 5 addresses the need to improve the response to post-crash emergencies and the ability of health and other systems to provide appropriate emergency treatment and long-term rehabilitation for crash victims. The development and improvement of pre-hospital care systems, hospital trauma care systems, and rehabilitation along with long-term medical support to victims and a single emergency response number, are the main elements of post-impact care.*

Suggested questions and prompts

- What is your job title and what is the focus of your responsibilities for this position?
- How long have you been in this role?
    - Prompt: How these responsibilities are determined?
- From the description of Pillar 5, what is the status of activities for this pillar in Nepal?
- From your experience, what is going well?
    - *Prompt: Why do you think it is going well?*
- From you experience what is left behind or not working well?
    - *Prompt: Why do you think it is not working well?*

For peer review only - http://bmjopen.bmj.com/site/about/guidelines.xhtml

- What challenges you are facing to achieve desired objectives for road safety?
    - *Prompts*
        - *why there is no single nationwide telephone number for emergency services i.e. ambulances?*
        - *How do you see the performance of hospital trauma services in Nepal?*
- What new information or evidence do you think would help improve the provision of good post-crash response and care in Nepal?
- Can you think of any gaps in the research or information available to you in regard to the recommendations in Pillar 5?
- Would you like to add something else which we have not covered during this conversation?

At the end of the interview

- Explain that this is the end of the interview.
- Thank the participant for their time.
- Explain that the information they have given will be used to create a list of possible research ideas to improve road safety in Nepal.
- Explain that they will be invited to the next stage of the study where they will hear all of the research questions we have identified and they will be invited to tell us which ones they think are the most important.
- Ask if they have any questions before you go.

Below is the Nepali translation of the Guide.

For peer review only - http://bmjopen.bmj.com/site/about/guidelines.xhtml

अन्तरवार्ता निर्देशिका

नेपालमा सडक सुरक्षाका लागि अनुसन्धान प्राथमिकताहरू पहिचान गर्ने अध्ययन

स्तम्भ १. सडक सुरक्षा व्यवस्थापन

(To be read to each participant for this pillar)

सडक सुरक्षाको यो स्तम्भ देशमा सडक सुरक्षाको लागि बहु-संस्थागत क्षमता सुदृढीकरणमा केन्द्रित छ। यस स्तम्भमा देशमा सडक सुरक्षाका लागि संयुक्त राष्ट्रसंघीय महासन्धीहरूलाई अभ्यास गर्ने, सडक सुरक्षा गतिविधिहरूको नेतृत्व गर्ने बहु-पक्षीय राष्ट्रिय निकायको स्थापना गर्ने, राष्ट्रिय सडक सुरक्षा रणनीति विकास गर्ने र सम्बन्धित गतिविधिहरूको कार्यान्वयनको लागि यथार्थवादी र दीर्घकालीन लक्ष्यहरूको निर्धारण गर्नाका साथै तिनको कार्यान्वयनका लागि पर्याप्त रकमको सुनिश्चतता गर्ने कृयाकलापहरू पर्दछन्। यस स्तम्भले उपर्युक्त कृयाकलापहरूको प्रभावकारी रूपमा अनुगमन र मूल्याङ्कन गर्नको लागि तथ्यांक प्रणालीको विकासको लागि पनि आह्वान गर्दछ।

Questions and prompts

- तपाई कुन पदमा कार्यरत हुनुहुन्छ र तपाईंको मुख्य जिम्मेवारीहरु के के हुन्?
- तपाई यस पदमा कति समयदेखि हुनुहुन्छ ?
    - प्रम्टः यस पदका लागि जिम्मेवारी कसरी निर्धारण भएका हुन्छन् ?
- स्तम्भ १ को वर्णनबाट तपाईं को विचारमा नेपालमा यस स्तम्भ अन्तर्गत गरिएका कृयाकलापहरूको अवस्था कस्तो छ ?
- तपाईको अनुभवबाट यस स्तम्भ अन्तरगतका कुन कुन कृयाकलापहरू राम्रोसँग अघि बढिरहेका छन् ?
    - प्रम्ट: तपाईंको विचारमा किन यो राम्रो हुँदैछ **?**
- तपाईको अनुभवमा यस स्तम्भ अन्तर्गतका कुन कामहरू राम्ररी भइरहेका छैन वा राम्रोसँग गर्न सकिएको छैन ?
    - प्रम्ट: तपाईंको विचारमा किन ती कृयाकलापहरू राम्रोसँग गर्न सकिएको छैन ?
- सडक सुरक्षाको लागि अपेक्षित उद्देश्यहरू प्राप्त गर्न तपाइँले के कस्ता चुनौतीहरू सामना गर्नु परेको छ ?
    - सडक सुरक्षाको नेतृत्व गर्ने संस्था वा निकायको स्थापनाबारे चुनौतिहरू?
    - राष्ट्रिय रणनीति बनाउने सम्बन्धी चुनौतिहरू?
    - तथ्यांक उत्पादन (Data generation) सम्बन्धी चुनौतिहरू?
    - अनुगमन र मूल्यांकन सम्बन्धी चुनौतिहरू?
- कस्ता जानकारी वा तथ्यहरू (Evidence) उपलब्ध भइदिएको भए तपाईंलाई यी उद्देश्यहरू प्राप्त गर्न मद्दत पुग्थ्यो जस्तो लाग्छ ?
- के तपाइँ स्तम्भ १ का यी गतिविधिहरूसँग सम्बन्धित अनुसन्धानमा कुनै कमी वा अन्तरहरू (Research Gaps) बारे सोचेर केही बताउन सक्नुहुन्छ ?
- हाम्रो यस कुराकानीको क्रममा केहि कुरा छुटेको पाउनु भए कृपया थप्नुहोस?

For peer review only - http://bmjopen.bmj.com/site/about/guidelines.xhtml

At the end of the interview

- हामी अन्तर्वार्ताको अन्त्यमा पुग्यौं
- तपाईंको सहभागिताको लागि धन्यवाद
- तपाईंले दिनुभएको जानकारीले नेपालमा सडक सुरक्षाको स्थितिमा सुधार ल्याउन सम्भावित अनुसन्धानको लागि सुची बनाउन मद्दत गर्नेछ।
- हामी तपाईलाई यस अध्ययनको दोस्रो चरणको लागि पनि सम्पर्क गर्नेछौं जसमा हामी अन्तरवार्ताहरूबाट पहिचान गरेका सबै अनुसन्धान प्रश्नहरु प्रस्तुत गर्नेछौं र त्यसमध्ये कुन सबैभन्दा महत्वपुर्ण छ भनेर तपाईंको प्रतिक्रिया लिनेछौं ।
- अन्तिममा तपाईंको केहि प्रश्न छ कि?

स्तम्भ २. सुरक्षित सडक र गतिशिलता

(To be read to each participant for this pillar)

यो स्तम्भले सबै पैदलयात्री, साइकल चालक, मोटरसाइकल चालक लगायत सबै सडक प्रयोगकर्ताको फाइदाको लागि आवश्यक सुरक्षित सडक संजाल एवं पूर्वाधार सुधारलाई जोड दिन्छ । यस अन्तरगतका कृयाकलापहरूमा सडकको योजना, निर्माण तथा संचालनका चरणहरूमा नैं सडक सुरक्षालाई ध्यानमा राख्नु पर्ने; सडकको सुरक्षाको नियमित मुल्यांकन गरिनेछ भनेर सुनिस्चित गर्ने; र सम्बद्ध अधिकारीलाई सडक प्रयोगकर्ताको गतिशिलता (mobility) आवश्यकतालाई सम्बोधन गर्दा सबै किसिम र प्रकारका यातायात तथा पूर्वाधारलाई सुरक्षित पार्न प्रोत्साहन गर्ने जस्ता क्रियाकलापहरु पर्छन् ।

Suggested questions and prompts

- तपाई कुन पदमा कार्यरत हुनुहुन्छ र तपाईंको मुख्य जिम्मेवारीहरु के के हुन्?
- तपाई यस पदमा कति समयदेखि हुनुहुन्छ ?
    - प्रम्प्ट: यस पदका लागि जिम्मेवारी कसरी निर्धारण भएका हुन्छन् ?
- स्तम्भ २ को वर्णनबाट तपाईं को विचारमा नेपालमा यस स्तम्भ अन्तरगत गरिएका कृयाकलापहरूको अवस्था कस्तो छ ?
- तपाईको अनुभवबाट यस स्तम्भ अन्तरगतका कुन कुन कृयाकलापहरू राम्रोसँग अघि बढिरहेका छन् ?
    - प्रम्प्ट: तपाईंको विचारमा किन यो राम्रो हुँदैछ ?
- तपाईको अनुभवमा यस स्तम्भ अन्तरगतका कुन कामहरू राम्ररी भइरहेका छैन वा राम्रोसँग गर्न सकिएको छैन ?
    - प्रम्प्ट: तपाईंको विचारमा किन ती कृयाकलापहरू राम्रोसँग गर्न सकिएको छैन ?
- सडक सुरक्षाको लागि अपेक्षित उद्देश्यहरू प्राप्त गर्न तपाइँले के कस्ता चुनौतीहरू सामना गर्दै हुनुहुन्छ ?
        - सडक सुरक्षाको स्वामित्व र उत्तरदायित्व प्रवर्धनसम्बन्धी चुनौती?
        - सबै सडक प्रयोगकर्ताहरूको आवश्यकतालाई सम्बोधन गर्नेबारे चुनौतीहरू?
        - सडक डिजाइन, निर्माण वा मर्मतसँग सम्बन्धी चुनौतिहरू?
- कस्ता जानकारी वा तथ्यहरू (Evidence) उपलब्ध भइदिएको भए तपाईंलाई यी उद्देश्यहरू प्राप्त गर्न मद्दत पुग्थ्यो जस्तो लाग्छ ?
- के तपाइँ स्तम्भ २ का यी गतिविधिहरुसंग सम्बन्धित अनुसन्धानमा कुनै कमी वा अन्तरहरू (Gaps) बारे सोचेर केही बताउन सक्नुहुन्छ ?

For peer review only - http://bmjopen.bmj.com/site/about/guidelines.xhtml

- हाम्रो यस कुराकानीको क्रममा केहि कुरा छुटेको पाउनु भए कृपया थप्नुहोस?

At the end of the interview
- हामी अन्तर्वार्ताको अन्त्यमा पुग्यौं
- तपाईंको सहभागिताको लागि धन्यवाद
- तपाईंले दिनुभएको जानकारीले नेपालमा सडक सुरक्षाको स्थितिमा सुधार ल्याउन सम्भावित अनुसन्धानको लागि सुची बनाउन मद्दत गर्नेछ।
- हामी तपाईलाई यस अध्ययनको दोस्रो चरणको लागि पनि सम्पर्क गर्नेछौं जसमा हामी अन्तरवार्ताहरूबाट पहिचान गरेका सबै अनुसन्धान प्रश्नहरु प्रस्तुत गर्नेछौं र त्यसमध्ये कुन सबैभन्दा महत्वपुर्ण छ भनेर तपाईंको प्रतिक्रिया लिनेछौं ।
- अन्तिममा तपाईंको केहि प्रश्न छ कि?

स्तम्भ ३ सुरक्षित वाहनहरू

(To be read to each participant for this pillar)
कमसल मापदण्ड भएका वाहनहरूले उल्लेखनीय संख्यामा सडक दुर्घटना र हताहती गराइरहेका हुन्छन्। यस स्तम्भले सुरक्षा प्रबर्धन गर्न उत्तम अभ्यास, वाहन सुरक्षाका मानकहरू र प्रविधिको प्रयोग गर्न प्रोत्साहन गर्दछ। यस अन्तरगतका गतिविधिहरूमा नयाँ कारको सुरक्षा मुल्यांकन कार्यक्रम (NCAP सुरक्षा रेटिंगहरू) को कार्यान्वयन गर्ने, विद्यमान सवारी साधनहरूमा न्यूनतम सुरक्षा सुविधाहरू भएको सुनिश्चत गर्न वाहन सुरक्षा जाँचहरू समावेश गर्ने, जस्तै दुर्घटनामा परेका व्यक्तिमा दुर्घटनाको प्रभाव कम गर्न सीट बेल्ट जोडिएको र बत्ती एवम् ब्रेक ठीक अवस्थामा छ भन्ने सुनिश्चत गर्दछ ।

<u>Suggested questions and prompts</u>
- तपाई कुन पदमा कार्यरत हुनुहुन्छ र तपाईंको मुख्य जिम्मेवारीहरु के के हुन्?
- तपाई यस पदमा कति समयदेखि हुनुहुन्छ ?
  - प्रम्ट: यस पदका लागि जिम्मेवारी कसरी निर्धारण भएका हुन्छन् ?
- स्तम्भ ३ को वर्णनबाट तपाईं को विचारमा नेपालमा यस स्तम्भ अन्तरगत गरिएका कृयाकलापहरूको अवस्था कस्तो छ ?
- तपाईको अनुभवबाट यस स्तम्भ अन्तरगतका कुन कुन कृयाकलापहरू राम्रोसँग अघि बढिरहेका छन् ?
  - <u>प्रम्ट</u>: तपाईंको विचारमा किन यो राम्रो हुँदैछ **?**
- तपाईको अनुभवमा यस स्तम्भ अन्तरगतका कुन कामहरू राम्ररी भइरहेका छैन वा राम्रोसँग गर्न सकिएको छैन ?
    - तपाईंको विचारमा किन ती कृयाकलापहरू राम्रोसँग गर्न सकिएको छैन ?
    - NCAP सुरक्षा रेटिंगहरू (अथवा नयाँ कारको सुरक्षा मुल्यांकन कार्यक्रम) सम्बन्धमा तपाइँको के विचार छ?
    - के हामीसँग सवारी साधन सम्बन्धी राम्रा कानूनहरू छन् जसले बढी सुरक्षित गाडीहरूको आयात बढाउने वा विद्यमान सवारी साधनहरूमा सुधार वा मर्मत गरी तिनलाइ सुरक्षित पार्न मद्दत गर्दछन्?
- सडक सुरक्षाको लागि अपेक्षित उद्देश्यहरू प्राप्त गर्न तपाइँले के कस्ता चुनौतीहरू सामना गर्दै हुनुहुन्छ ?
    - राष्ट्रिय कानूनहरूलाई अन्तर्राष्ट्रिय मोटर वाहन सुरक्षा नियमहरुसँग सामंजस्यता गर्दाका चुनौतिहरु?

For peer review only - http://bmjopen.bmj.com/site/about/guidelines.xhtml

- जोखिममा रहेका सडक प्रयोगकर्ताहरूका जोखिम कम गर्न डिजाइन गरिएको सुरक्षा प्रविधिहरूका बारे अनुसन्धानसम्बन्धी चुनौतिहरुरू
- कस्ता जानकारी वा तथ्यहरू (Evidence) उपलव्ध भइदिएको भए तपाईंलाई यी उद्देश्यहरू प्राप्त गर्न मद्दत पुग्थ्यो जस्तो लाग्छ ?
- के तपाइँ स्तम्भ ३ का यी गतिविधिहरुसंग सम्बन्धित अनुसन्धानमा कुनै कमी वा अन्तरहरू (Gaps) बारे सोचेर केही बताउन सक्नुहुन्छ ?
- हाम्रो यस कुराकानीको क्रममा केहि कुरा छुटेको पाउनु भए कृपया थप्नुहोस?

At the end of the interview

- हामी अन्तर्वार्ताको अन्त्यमा पुग्यौं
- तपाईंको सहभागिताको लागि धन्यवाद
- तपाईंले दिनुभएको जानकारीले नेपालमा सडक सुरक्षाको स्थितिमा सुधार ल्याउन सम्भावित अनुसन्धानको लागि सुची बनाउन मद्दत गर्नेछ।
- हामी तपाईलाई यस अध्ययनको दोस्रो चरणको लागि पनि सम्पर्क गर्नेछौं जसमा हामी अन्तरवार्ताहरूबाट पहिचान गरेका सबै अनुसन्धान प्रश्नहरु प्रस्तुत गर्नेछौं र त्यसमध्ये कुन सबैभन्दा महत्वपुर्ण छ भनेर तपाईंको प्रतिक्रिया लिनेछौं ।
- अन्तिममा तपाईंको केहि प्रश्न छ कि?

स्तम्भ ४ सुरक्षित सडक प्रयोगकर्ताहरू

(To be read to each participant for this pillar)
स्तम्भ ४ सबै सडक प्रयोगकर्ताहरूको व्यवहार सुधार गर्न व्यापक कार्यक्रमहरू विकासमा केन्द्रित छ। यस अन्तरगत उदाहरणीय सडक सुरक्षा कानून र मापदण्ड अपनाउने र तिनको निरन्तर वा बढ्दो क्रममा पालनामा गराउने गतिविधिहरू समावेश छन। यस्ता प्रयासहरूमा जनचेतना र मानिसहरुलाई सुरक्षित राख्ने उपायहरू जस्तैः सीट बेल्ट र हेलमेट लगाउने बानी प्रवर्द्धन गर्न र तीव्र गतिमा वाहन चलाउने, रक्सी वा मादकपदार्थ सेवन गरी गाडी चलाउने र यस्तै अन्य जोखिमपूर्ण व्यवहारमा कमी ल्याउन व्यवहारिक शिक्षा दिने कृयाकलापहरू पनि समावेश छन्। यस स्तम्भले कामसँग सम्बन्धित (पेशागत) सडक दुर्घटनाहरू कम गर्ने गतिविधिहरूको लागि आह्वान गर्दछ र भरखरै चालक अनुमतिपत्र (लाइसेन्स) लिएका नयाँ चालकहरूको लागि उनीहरूको सवारी चालक अनुमतिपत्र (लाइसेन्स) लाई क्रमैसँग स्तरोन्नति गर्ने (graduated driver licensing) कार्यक्रमहरूलाई बढावा दिन अह्वान गरेको छ।

Suggested questions and prompts

- तपाई कुन पदमा कार्यरत हुनुहुन्छ र तपाईंको मुख्य जिम्मेवारीहरु के के हुन्?
- तपाई यस पदमा कति समयदेखि हुनुहुन्छ ?
    - प्रम्प्ट: यस पदका लागि जिम्मेवारी कसरी निर्धारण भएका हुन्छन् ?
- स्तम्भ ४ को वर्णनबाट तपाईं को विचारमा नेपालमा यस स्तम्भ अन्तरगत गरिएका कृयाकलापहरूको अवस्था कस्तो छ ?
- तपाईको अनुभवबाट यस स्तम्भ अन्तरगतका कुन कुन कृयाकलापहरू राम्रोसँग अघि बढिरहेका छन् ?
    - प्रम्प्ट: तपाईंको विचारमा किन यो राम्रो हुँदैछ **?**

For peer review only - http://bmjopen.bmj.com/site/about/guidelines.xhtml

- तपाईको अनुभवमा यस स्तम्भ अन्तरगतका कुन कामहरू राम्ररी भइरहेका छैन वा राम्रोसँग गर्न सकिएको छैन ?
    - तपाईंको विचारमा किन ती कृयाकलापहरू राम्रोसँग गर्न सकिएको छैन ?
- सडक सुरक्षाको लागि अपेक्षित उद्देश्यहरू प्राप्त गर्न तपाइँले के कस्ता चुनौतीहरू सामना गर्दै हुनुहुन्छ ?
        - कानूनको पालना र कार्यान्वयनको स्थिति कस्तो छ?
        - सडक सुरक्षा कानूनको पालनालाई सुदृढ पार्न के गर्न सकिन्छ?
        - सुरक्षित चालक / यात्री व्यवहारको अवलम्बन गर्ने चलन कस्तो छ (उदाहरणको लागि सीटबेल्ट / हेलमेट प्रयोग, मा.प.से.)
        - सडक सुरक्षाको लागि चालक अनुमतिपत्र (लाइसेन्स) के भूमिका खेल्छ?
        - सडक प्रयोगकर्ताका व्यवहारसँग सम्बन्धित कुनैं कमीकमजोरीहरू हामीलाई थाहा छन् ? तिनलाई कसरी परिवर्तन गर्न सकिन्छ ?
        - सडक सुरक्षा सम्बन्धी कानूनी प्रावधानमा के कस्ता कमी कमजोरी छन् ? तिनको पालनालाई कसरी सडक प्रयोगकर्ताहरूको व्यवहारसँग जोडिएकोछ ?
- कुन नयाँ जानकारी वा तथ्य उपलब्ध भइदिए सडक प्रयोगकर्ताहरूको सुरक्षित व्यवहार अवलम्बनलाई सुधार गर्न मद्दत पुग्नेछ?
- के तपाइँ स्तम्भ ४ का यी गतिविधिहरुसंग सम्बन्धित अनुसन्धानमा कुनै कमी वा अन्तरहरू (Gaps) बारे सोचेर केही बताउन सक्नुहुन्छ ?
- हाम्रो यस कुराकानीको क्रममा केहि कुरा छुटेको पाउनु भए कृपया थप्नुहोस?

At the end of the interview

- हामी अन्तर्वार्ताको अन्त्यमा पुग्यौं
- तपाईंको सहभागिताको लागि धन्यवाद
- तपाईंले दिनुभएको जानकारीले नेपालमा सडक सुरक्षाको स्थितिमा सुधार ल्याउन सम्भावित अनुसन्धानको लागि सुची बनाउन मद्दत गर्नेछ।
- हामी तपाईलाई यस अध्ययनको दोस्रो चरणको लागि पनि सम्पर्क गर्नेछौं जसमा हामी अन्तरवार्ताहरूबाट पहिचान गरेका सबै अनुसन्धान प्रश्नहरु प्रस्तुत गर्नेछौं र त्यसमध्ये कुन सबैभन्दा महत्वपुर्ण छ भनेर तपाईंको प्रतिक्रिया लिनेछौं ।
- अन्तिममा तपाईंको केहि प्रश्न छ कि?

स्तम्भ ५. दुर्घटनापश्चातको स्याहार

(To be read to each participant for this pillar)

स्तम्भ ५ ले दुर्घटना पछिको इमरजेन्सीकोलागि प्रतिक्रियामा सुधारको साथै दुर्घटनाका घाइतेलाई उपयुक्त इमरजेन्सी उपचार सेवा एवम् दीर्घकालीन पुनर्स्थापना सेवा दिने स्वास्थ्य र अन्य प्रणालीको क्षमताको सुधारको आवश्यकतालाई सम्बोधन गर्दछ। घाइतेलाई अस्पताल लैजानु अघि गर्नु पर्ने स्याहार प्रणाली, अस्पताल ट्रामा स्याहार प्रणाली, पीडितलाई पुनर्स्थापनाका साथै दीर्घकालीन चिकित्सा सहायता प्रणालीको विकास एवम् सुधार र इमरजेन्सी अवस्थामा सहयोग लिन प्रयोग गरिने एउटै नम्बरको बिकास र सुधारका कृयाकलापहरू दुर्घटना पश्चातको स्याहारका मुख्य बुँदाहरू हुन् ।

Suggested questions and prompts

- तपाई कुन पदमा कार्यरत हुनुहुन्छ र तपाईंको मुख्य जिम्मेवारीहरु के के हुन्?
- तपाई यस पदमा कति समयदेखि हुनुहुन्छ ?

For peer review only - http://bmjopen.bmj.com/site/about/guidelines.xhtml

  - प्रम्ट: यस पदका लागि जिम्मेवारी कसरी निर्धारण भएका हुन्छन् ?
- स्तम्भ १ को वर्णनबाट तपाईं को विचारमा नेपालमा यस स्तम्भ अन्तरगत गरिएका कृयाकलापहरूको अवस्था कस्तो छ ?
- तपाईको अनुभवबाट यस स्तम्भ अन्तरगतका कुन कुन कृयाकलापहरू राम्रोसँग अघि बढिरहेका छन् ?
  - प्रम्ट: तपाईंको विचारमा किन यो राम्रो हुँदैछ ?
- तपाईको अनुभवमा यस स्तम्भ अन्तरगतका कुन कामहरू राम्ररी भइरहेका छैन वा राम्रोसँग गर्न सकिएको छैन ?
  - तपाईंको विचारमा किन ती कृयाकलापहरू राम्रोसँग गर्न सकिएको छैन ?
- सडक सुरक्षाको लागि अपेक्षित उद्देश्यहरू प्राप्त गर्न तपाइँले के कस्ता चुनौतीहरू सामना गर्नु परेको छ ?
    - किन देशभरी नै आपत्कालिन सेवा जस्तै एम्बुलेन्स सेवाको लागी एउटै टेलिफोन नम्बर छैन ?
    - तपाईं नेपालका अस्पतालको ट्रमा सेवाहरूलाई कसरी हेर्नुहुन्छ ?
- कुन नयाँ जानकारी वा तथ्य उपलब्ध भइदिए सडक प्रयोगकर्ताहरूको सुरक्षित व्यवहार अवलम्बनलाई सुधार गर्न मद्दत पुग्नेछ?
- के तपाइँ स्तम्भ ५ का यी गतिविधिहरुसंग सम्बन्धित अनुसन्धानमा कुनै कमी वा अन्तरहरू (Gaps) बारे सोचेर केही बताउन सक्नुहुन्छ ?
- हाम्रो यस कुराकानीको क्रममा केहि कुरा छुटेको पाउनु भए कृपया थप्नुहोस?

At the end of the interview

- हामी अन्तर्वार्ताको अन्त्यमा पुग्यौं
- तपाईंको सहभागिताको लागि धन्यवाद
- तपाईले दिनुभएको जानकारीले नेपालमा सडक सुरक्षाको स्थितिमा सुधार ल्याउन सम्भावित अनुसन्धानको लागि सुची बनाउन मद्दत गर्नेछ।
- हामी तपाईलाई यस अध्ययनको दोस्रो चरणको लागि पनि सम्पर्क गर्नेछौं जसमा हामी अन्तरवार्ताहरूबाट पहिचान गरेका सबै अनुसन्धान प्रश्नहरु प्रस्तुत गर्नेछौं र त्यसमध्ये कुन सबैभन्दा महत्वपुर्ण छ भनेर तपाईंको प्रतिक्रिया लिनेछौं ।
- अन्तिममा तपाईंको केहि प्रश्न छ कि?

For peer review only - http://bmjopen.bmj.com/site/about/guidelines.xhtml

CREDES checklist adapted from Saskia et al 2017. *Guidance on Conducting and REporting DElphi Studies (CREDES) in palliative care: Recommendations based on a methodological systematic review*. Palliative Medicine. available from https://journals.sagepub.com/doi/10.1177/0269216317690685

Box 3. Recommendations for the Conducting and REporting of DElphi Studies (CREDES).

Rationale for the choice of the Delphi technique

1. *Justification.* The choice of the Delphi technique as a method of systematically collating expert consultation and building consensus needs to be well justified. When selecting the method to answer a particular research question, it is important to keep in mind its constructivist nature

Planning and design

2. *Planning and process.* The Delphi technique is a flexible method and can be adjusted to the respective research aims and purposes. Any modifications should be justified by a rationale and be applied systematically and rigorously
3. *Definition of consensus.* Unless not reasonable due to the explorative nature of the study, an a priori criterion for consensus should be defined. This includes a clear and transparent guide for action on (a) how to proceed with certain items or topics in the next survey round, (b) the required threshold to terminate the Delphi process and (c) procedures to be followed when consensus is (not) reached after one or more iterations

Study conduct

4. *Informational input.* All material provided to the expert panel at the outset of the project and throughout the Delphi process should be carefully reviewed and piloted in advance in order to examine the effect on experts' judgements and to prevent bias
5. *Prevention of bias.* Researchers need to take measures to avoid directly or indirectly influencing the experts' judgements. If one or more members of the research team have a conflict of interest, entrusting an independent researcher with the main coordination of the Delphi study is advisable
6. *Interpretation and processing of results.* Consensus does not necessarily imply the 'correct' answer or judgement; (non)consensus and stable disagreement provide informative insights and highlight differences in perspectives concerning the topic in question
7. *External validation.* It is recommended to have the final draft of the resulting guidance on best practice in palliative care reviewed and approved by an external board or authority before publication and dissemination

Reporting

8. *Purpose and rationale.* The purpose of the study should be clearly defined and demonstrate the appropriateness of the use of the Delphi technique as a method to achieve the research aim. A rationale for the choice of the Delphi technique as the most suitable method needs to be provided
9. *Expert panel.* Criteria for the selection of experts and transparent information on recruitment of the expert panel, socio-demographic details including information on expertise regarding the topic in question, (non)response and response rates over the ongoing iterations should be reported
10. *Description of the methods.* The methods employed need to be comprehensible; this includes information on preparatory steps (How was available evidence on the topic in question synthesised?), piloting of material and survey instruments, design of the survey instrument(s), the number and design of survey rounds, methods of data analysis, processing and synthesis of experts' responses to inform the subsequent survey round and methodological decisions taken by the research team throughout the process
11. *Procedure.* Flow chart to illustrate the stages of the Delphi process, including a preparatory phase, the actual 'Delphi rounds', interim steps of data processing and analysis, and concluding steps
12. *Definition and attainment of consensus.* It needs to be comprehensible to the reader how consensus was achieved throughout the process, including strategies to deal with non-consensus
13. *Results.* Reporting of results for each round separately is highly advisable in order to make the evolving of consensus over the rounds transparent. This includes figures showing the average group response, changes between rounds, as well as any modifications of the survey instrument such as deletion, addition or modification of survey items based on previous rounds
14. *Discussion of limitations.* Reporting should include a critical reflection of potential limitations and their impact of the resulting guidance
15. *Adequacy of conclusions.* The conclusions should adequately reflect the outcomes of the Delphi study with a view to the scope and applicability of the resulting practice guidance
16. *Publication and dissemination.* The resulting guidance on good practice in palliative care should be clearly identifiable from the publication, including recommendations for transfer into practice and implementation. If the publication does not allow for a detailed presentation of either the resulting practice guidance or the methodological features of the applied Delphi technique, or both, reference to a more detailed presentation elsewhere should be made (e.g. availability of the full guideline from the authors or online; publication of a separate paper reporting on methodological details and particularities of the process (e.g. persistent disagreement and controversy on certain issues)). A dissemination plan should include endorsement of the guidance by professional associations and health care authorities to facilitate implementation

Item#	Description	Section/Page # reported in the manuscript
Rationale for the choice of the Delphi technique		
1	Justification.	Methods, first paragraph, page 4
Planning and design		
2	Planning and process	METHODS, pages 4-6

3	Definition of Consensus	Data collection and analysis, page 5, paragraph 2
Study conduct		
4	Informational input	Recruitment of participants, page 4
5	Prevention of bias	Strengths and limitations, page 14-15
6	Interpretation and processing results	Data collection, paragraphs 2-4, pages 5-6
7	External validations	Overarching consensus workshop, page 6.
Reporting		
8	Purpose and Rationale	INTRODUCTION, page 3
9	Expert panel	Description of participants, Results, pages 6-7 and Table 1
10	Description of the methods	METHODS, pages 4-6
11	Procedure	METHODS, pages 4-6 Flow chart, Figure 1, page 19
12	Definition and attainment of consensus	Paragraphs 2 and 3 of Data collection and analysis, pages 5-6 + overall consensus workshop paragraph, page 6
13	Results	Results pages 6-11, Table 3 (page 9-11) and Table 4 (page 11)
14	Discussion of limitations	Strengths and limitations, page 14
15	Adequacy of conclusions	CONCLUSIONS, page 15
16	Publication and dissemination	Not applicable as this is not a Delphi study supporting guidelines for clinical practice.

For peer review only
